# C@PA: Computer-Aided Pattern Analysis to Predict Multitarget ABC Transporter Inhibitors

**DOI:** 10.1021/acs.jmedchem.0c02199

**Published:** 2021-03-16

**Authors:** Vigneshwaran Namasivayam, Katja Silbermann, Michael Wiese, Jens Pahnke, Sven Marcel Stefan

**Affiliations:** † Department of Pharmaceutical and Cellbiological Chemistry, Pharmaceutical Institute, University of Bonn, An der Immenburg 4, 53121 Bonn, Germany; ‡ Department of Neuro-/Pathology, University of Oslo and Oslo University Hospital, Sognsvannsveien 20, 0372 Oslo, Norway; § LIED, University of Lübeck, Ratzenburger Allee 160, 23538 Lübeck, Germany; ∥ Department of Pharmacology, Faculty of Medicine, University of Latvia, Jelgavas iela 1, 1004 Riga, Latvia; ⊥ Department of Bioorganic Chemistry, Leibniz-Institute of Plant Biochemistry, Weinberg 3, 06120 Halle, Germany; ⊗ Cancer Drug Resistance and Stem Cell Program, University of Sydney, Kolling Building, 10 Westbourne Street, Sydney, New South Wales 2065, Australia

## Abstract

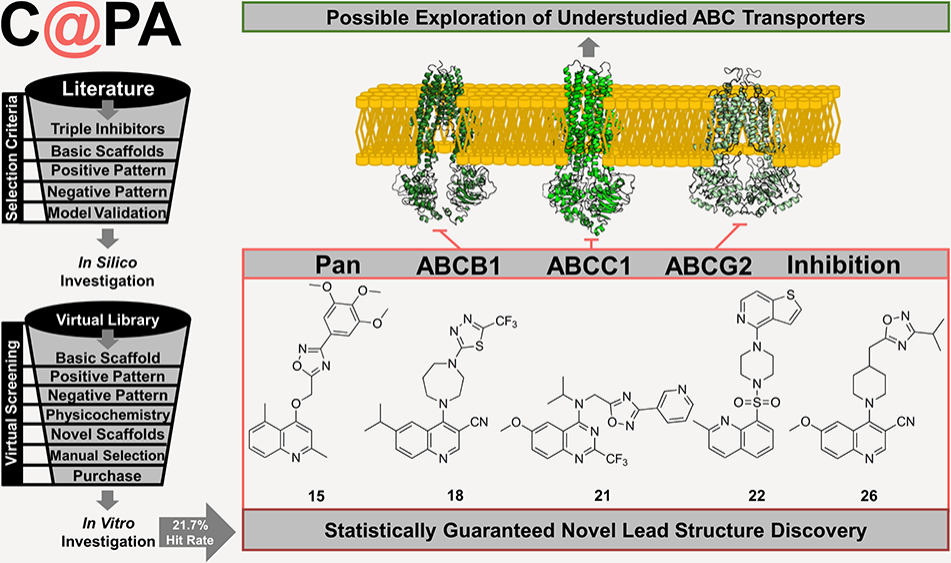

Based on literature reports of the last two decades, a computer-aided pattern analysis (C@PA) was implemented for the discovery of novel multitarget ABCB1 (P-gp), ABCC1 (MRP1), and ABCG2 (BCRP) inhibitors. C@PA included basic scaffold identification, substructure search and statistical distribution, as well as novel scaffold extraction to screen a large virtual compound library. Over 45,000 putative and novel broad-spectrum ABC transporter inhibitors were identified, from which 23 were purchased for biological evaluation. Our investigations revealed five novel lead molecules as triple ABCB1, ABCC1, and ABCG2 inhibitors. C@PA is the very first successful computational approach for the discovery of promiscuous ABC transporter inhibitors.

## Introduction

Expression of adenosine triphosphate-(ATP)-binding cassette (ABC) transporters in multidrug-resistant cancer remains a huge obstacle in cancer chemotherapy. Many of the 49 ABC transporters confer resistance to structurally and functionally diverse antineoplastic agents,^1^ leading to the multidrug resistance (MDR) phenotype. However, small-molecule inhibitors to target ABC transporters are only known for a fraction of these 49 transporters. Amongst these are the three well-studied transporters ABCB1 (P-glycoprotein, P-gp), ABCC1 (multidrug resistance-associated protein 1, MRP1), and ABCG2 (breast cancer resistance protein, BCRP), for which a bunch of potent (and mostly specific) small-molecule inhibitors has been generated over the last four decades.^2−4^ Unfortunately, clinical studies approaching one single transporter with selective and highly potent agents have mostly failed.^5−7^ Two concluding postulations emerged very recently: (i) ABC transporters have a differing (individual) substrate range, which increases cross-resistance in case of their co-expression.^6,8^ These individual substrate ranges combined cover almost the whole range of today’s applied antineoplastic agents;^5−8^ (ii) ABC transporters have also an overlapping (collective) substrate range, enabling them to compensate for the selective inhibition and/or downregulation of their functional counterpart(s). These collective substrate ranges account for a regulatory dependency of ABC transporter expression in terms of a triggered upregulation.^6,8^ Both simultaneous overexpression of ABC transporters^9,10^ and compensation^11−13^ have already been documented in the literature. This ultimately leads to maintaining, extending, and/or shifting of the resistance profile of multidrug-resistant cancer.^6^ Hence, multitarget ABC transporter inhibition might be a novel and promising approach to treat multidrug-resistant cancer. However, the simultaneous targeting of ABCB1, ABCC1, and ABCG2 has only very recently been emphasized.^6,14−17^ The term broad-spectrum inhibition itself goes back to mid-2000s.^18^ Since then, it was only infrequently acknowledged^19−22^ and has only been addressed properly within the last couple of years.^6,14−17,23,24^

**Figure 1:**
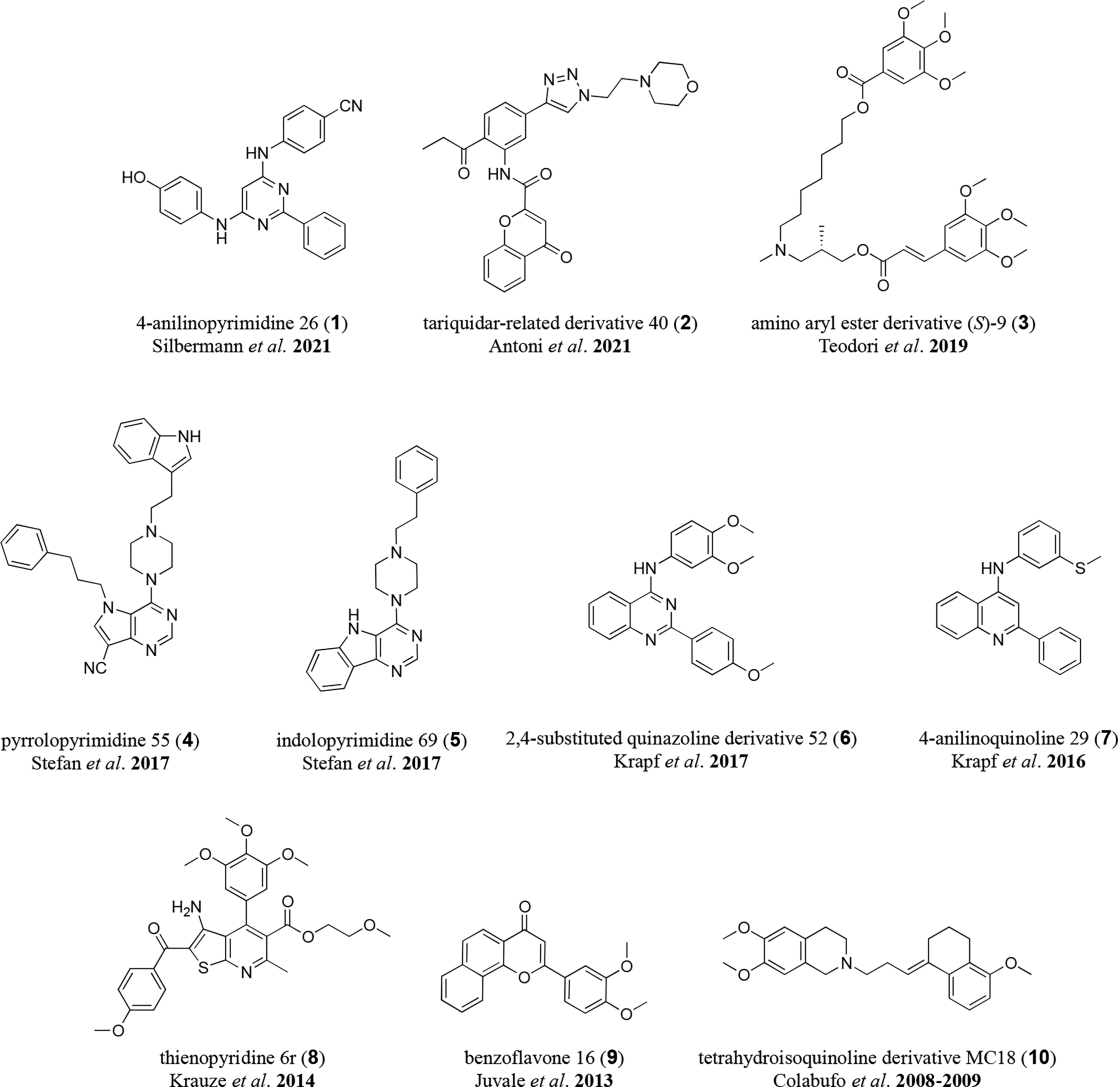
Depiction of the most potent triple ABCB1, ABCC1, and ABCG2 inhibitors derived by HTS and synthesis approaches: 4-anilinopyrimidine 26 (**1**) as reported by Silbermann *et al.* in 2021;^14^ the tariquidar-related derivative 40 (**2**) as reported by Antoni *et al*. in 2021;^23^ the amino aryl ester derivative (*S*)-9 (**3**) as reported by Teodori *et al*. in 2019;^26^ pyrrolopyrimidine 55 (**4**) and indolopyrimidine 71 (**5**) as reported by Stefan *et al*. in 2017;^17^ the 2,4-substituted quinazoline derivative 52 (**6**) as reported by Krapf *et al.* in 2017;^28^ 4-anilinoquinoline 29 (**7**) as reported by Krapf *et al*. in 2016;^29^ thienopyridine 6r (**8**) as reported by Krauze *et al*. in 2014;^32^ benzoflavone 16 (**9**) as reported by Juvale *et al*. in 2013;^34^ and the tetrahydroisoquinoline derivative MC18 (**10**) as reported by Colabufo *et al*. in 2008^39^ and 2009.^21^

Less than 1200 compounds have been evaluated *in vitro* for ABCB1, ABCC1, and ABCG2 inhibition, of which less than 140 can be considered as broad-spectrum inhibitors. While around 50 compounds exerted their ABC transporter inhibiting property below 10 μM for each transporter,^14−17,21,23,25−42^ only 22 compounds had activities below 5 μM.^14,15,21,23,25,26,28,32,34,37−39^ Amongst the most potent triple ABCB1, ABCC1, and ACBG2 inhibitors are 4-anilinopyrimidine 26 (**1**),^14^ the tariquidar-related derivative 40 (**2**),^23^ the amino aryl ester derivative (*S*)-9 (**3**),^26^ pyrrolopyrimidine 55 (**4**),^17^ indolopyrimidine 69 (**5**),^17^ the 2,4-substituted quinazoline derivative 52 (**6**),^28^ 4-anilinoquinoline 29 (**7**),^29^ thienopyridine 6r (**8**),^32^ benzoflavone 16 (**9**),^34^ and the tetrahydroisoquinoline derivative MC18 (**10**)^21,39^ (Figure 1).

Computational approaches with respect to ABC transporter inhibition have been undertaken^43,44^ mostly focusing on selective inhibition of ABCB1,^45,46^ ABCC1,^16^ or ABCG2^47^ individually. No approach took inhibitors of more than one ABC transport protein into account. However, this would revolutionize our understanding of ABC transporters as this could address two major aspects: (i) identification of structural requirements for a simultaneous targeting of ABCB1, ABCC1, and ABCG2 and, vice versa, identification of structural features for selective inhibition of one of these transporters; and (ii) potentially deciphering common structural features to address other ABC transporters that are not able to be targeted by small-molecules until now. In order to give way for the discovery and development of novel broad-spectrum ABC transporter modulators, we implemented C@PA, a computer-aided pattern analysis, which is presented in this work.

## Results

### Computational Analysis

#### Compilation of Data Set and Classification of Compounds

As a first step, 93 reports between 2004 and 2021 were collected in which the evaluation of small-molecule inhibitors of ABCB1, ABCC1, and ABCG2 was described. Reports that did not include biological investigations on all three transporters were not considered, as a subsequent classification of the compounds would fail due to missing activity value(s). The half-maximal inhibition concentration (IC_50_) values of the compounds were considered as the major indicator of direct inhibition. Other biological data that was not based on tracing of (immediate) ABC transporter-mediated transport (*e.g.*, by a fluorescence dye or a radionuclide) was not taken into account as these surrogates [*e.g.*, the half-maximal reversal concentrations (EC_50_) obtained in MDR reversal assays] and their observed effects (*e.g.*, the shift in toxicity of a co-administered antineoplastic agent) may not be (directly) linked to inhibition of transport activity alone but also to unspecific, non-ABC transporter-related targets. The IC_50_ values were either extracted from tables as reported in the respective publication or estimated from relative inhibition (I_rel_) values compared to the maximal inhibition exerted by a standard inhibitor (I_max_). In the latter case, the IC_50_ was categorized into <10 μM (“active”) or ≥10 μM (“inactive”). The dataset including compound names and SMILES codes, inhibitory activity values against ABCB1, ABCC1, and ABCG2, used cell lines and testing systems, as well as the links to the corresponding literature can be found in Supplementary Table 1.

In total, 1049 compounds were identified, which have been evaluated at least once regarding ABCB1, ABCC1, and ABCG2. In case a compound has been evaluated in more than one assay, the mean of the reported IC_50_ values was taken for further analysis. In case one compound was evaluated with a definite number (*e.g.*, 9.6 μM) and an estimation (*e.g.*, >25 μM), the definite number was always given priority, while the estimated value was not considered. The same accounts for a compound that was classified as “inactive” in one assay and associated with a definite IC_50_ value in another assay. If a range was given (*e.g.*, 4–5 μM), the mean has been taken for further analysis (*e.g.*, 4.5 μM). The dataset for ongoing analysis, including compound names and SMILES codes, can be found in Supplementary Table 2.

In a next step, the compounds of Supplementary Table 2 were categorized into “active” [1 (“one”); IC_50_ value <10 μM] and “inactive” [0 (“zero”); IC_50_ values ≥10 μM]. As a result, 256 compounds were found to be active against ABCB1, while 793 were inactive. Concerning ABCC1, 147 were active, while 902 were found to be inactive. Finally, regarding ABCG2, 629 representatives were found as active, and 420 were inactive. Considering their activity profile against ABCB1, ABCC1, and ABCG2, the 1049 compounds were classified into the following eight classes (0–7): (i) class 0 consisted of 276 molecules that had no effect on either ABCB1, ABCC1, or ABCG2 (0, 0, 0); (ii) class 1 comprised 69 selective ABCB1 inhibitors (1, 0, 0); (iii) class 2 contained 58 selective ABCC1 inhibitors (0, 1, 0); (iv) class 3 included 435 selective ABCG2 inhibitors (0, 0, 1); (v) class 4 consisted of 17 dual ABCB1 and ABCC1 inhibitors (1, 1, 0); (vi) class 5 comprised 122 dual ABCB1 and ABCG2 inhibitors (1, 0, 1); (vii) class 6 contained 24 dual ABCC1 and ABCG2 inhibitors (0, 1, 1); and (viii) class 7 included 48 multitarget ABCB1, ABCC1, and ABCG2 inhibitors (1, 1, 1). Supplementary Table 3 provides all 1049 classified compounds with names and SMILES codes.

#### Basic Scaffold Search and Statistical Substructure Analysis

**Figure 2:**
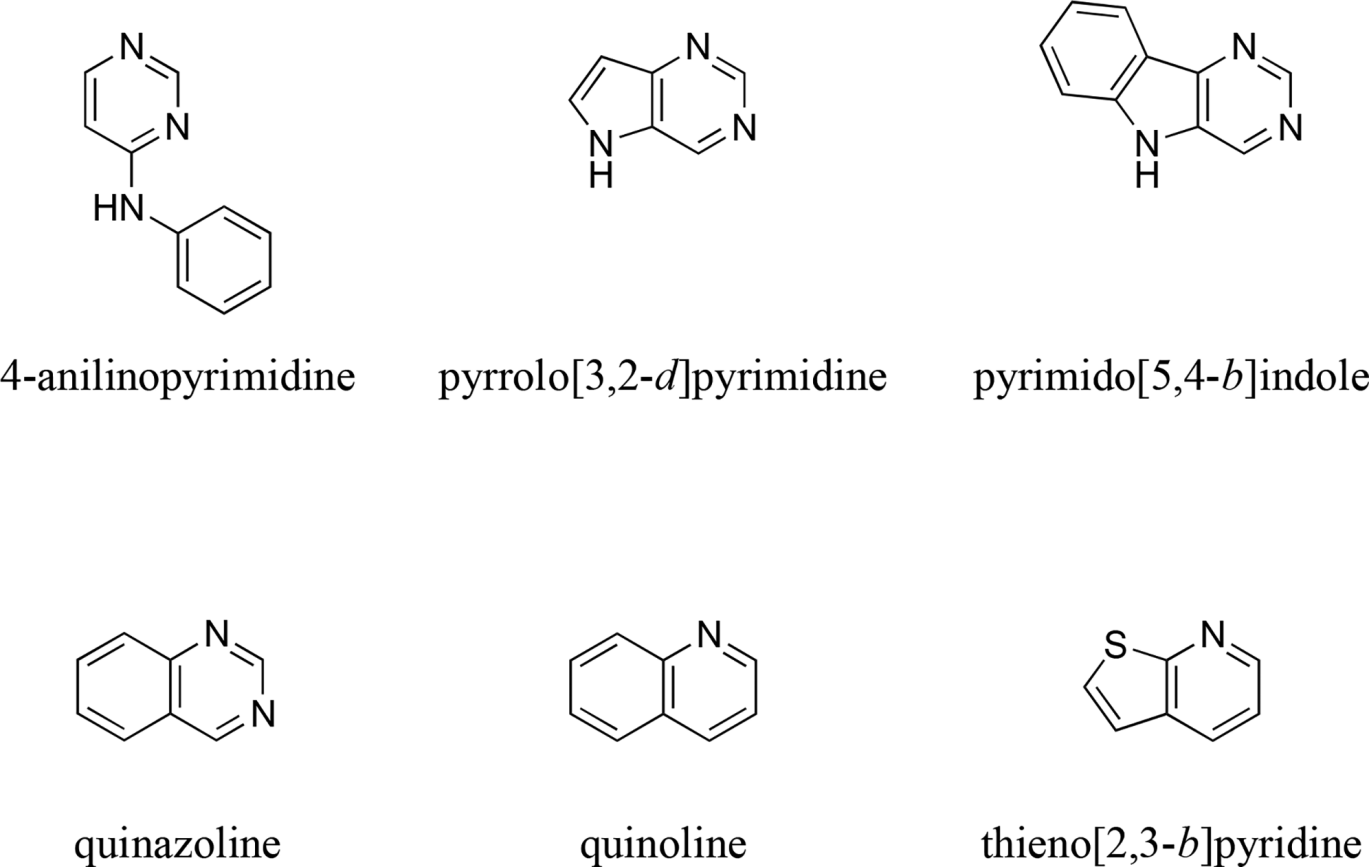
Basic scaffolds of the 48 triple ABCB1, ABCC1, and ABCG2 inhibitors using the Structure-Activity-Report (SAReport) tool^48^ implemented in Molecular Operating Environment (MOE).^49^

Two main questions should be addressed to identify the critical fingerprints for triple ABCB1, ABCC1, and ABCG2 inhibition (“multitarget fingerprints”): (i) which basic scaffolds do the 48 compounds of class 7 have and (ii) what structural features must be present for promiscuity toward ABCB1, ABCC1, and ABCG2? To address the first question, a scaffold analysis of class 7 compounds was conducted using the Structure-Activity-Report (SAReport) tool^48^ implemented in Molecular Operating Environment (MOE).^49^ From these 48 triple ABCB1, ABCC1, and ABCG2 inhibitors, 35 could be categorized into six different scaffolds: (i) 4-anilinopyrimidine, (ii) pyrrolo[3,2-*d*]pyrimidine, (iii) pyrimido[5,4-*b*]indole, (iv) quinazoline, (v) quinoline, and (vi) thieno[2,3-*b*]pyridine. Figure 2 visualizes these six basic scaffolds.

Vice versa, 13 inhibitors could not be categorized, from which 11 did not have a heteroaromatic core structure. Regarding the other 2, one compound was the only representative of its structural class (thieno[2,3-*b*]pyrimidine).^16^ The other compound (apatinib) contained a pyridine,^35^ which was only present in three molecules and therefore did not constitute a heteroaromatic (basic) scaffold on its own according to the SAReport.^48^ Nevertheless, two features of these 13 non-categorizable ABCB1, ABCC1, and ABCG2 inhibitors should be highlighted: (i) the thieno[2,3-*b*]pyrimidine and pyridine scaffolds could be sub-categories of the thieno[2,3-*b*]pyridine and quinoline scaffolds, respectively; and (ii) 9 of the 13 compounds had either dimethoxyphenyl (3 compounds) or trimethoxyphenyl (6 compounds) partial structures, which could be markers for multitarget inhibition.

To address the second question as indicated above, a list of in total 308 partial structures was compiled that are commonly present in organic molecules^50^ (names and SMILES codes can be found in Supplementary Table 4). The eight classes were screened against these 308 partial structures using the tool InstantJChem,^51^ and the absolute statistical distribution of each partial structure was collected. The relative statistical distribution was calculated, which represented the percentage of occurrence of the corresponding partial structure within the respective class (0–7). As a next step, structural markers were searched for that clearly favored triple ABCB1, ABCC1, and ABCG2 inhibition. For this purpose, the relative statistical distribution was reorganized in five different groups: (i) group A represented the percentage of class 0 (inactive molecules); (ii) group B represented the summed percentages of classes 1–3 (selective inhibitors); (iii) group C represented the summed percentages of classes 4–6 (dual inhibitors); (iv) group D represented the percentages of class 7 (triple inhibitors); and (v) group E was calculated from the sum of the percentages of classes 4–7 [dual and triple (= multitarget) inhibitors].

#### Identification of Multitarget Fingerprints

From Supplementary Table 4, “clear positive hits” (“Positive Pattern”) could be deduced. These were defined as the following: (i) the respective substructure must have occurred at least five times in the 1049 molecules of the dataset; (ii) group D must have had accounted for at least 15% of the respective hit molecules; and (iii) the percentage of group D should have been at least the same as the percentage of group B. If the second point was fulfilled but the third was not, (iv) the percentage of group E must have been at least the percentage of group B. Applying these rules, nine substructures could be found as potential markers for triple ABCB1, ABCC1, and ABCG2 inhibition: (i) isopropyl; (ii) amino; (iii) carboxylic acid ethyl ester; (iv) indole; (v) 3,4,5-trimethoxyphenyl; (vi) morpholine; (vii) thieno[2,3-*b*]pyridine; (viii) sulfoxide; and (ix) sulfone (Supplementary Table 4). A detailed analysis of the latter two partial substructures revealed that none of the 1049 compounds contained a sulfoxide residue but only sulfones, of which sulfoxide is a part of. Hence, we accepted only sulfone as clear positive hit for triple ABCB1, ABCC1, and ABCG2 inhibition. The thieno[2,3-*b*]pyridine substructure was for its part already found in the basic scaffold search.

**Figure 3:**
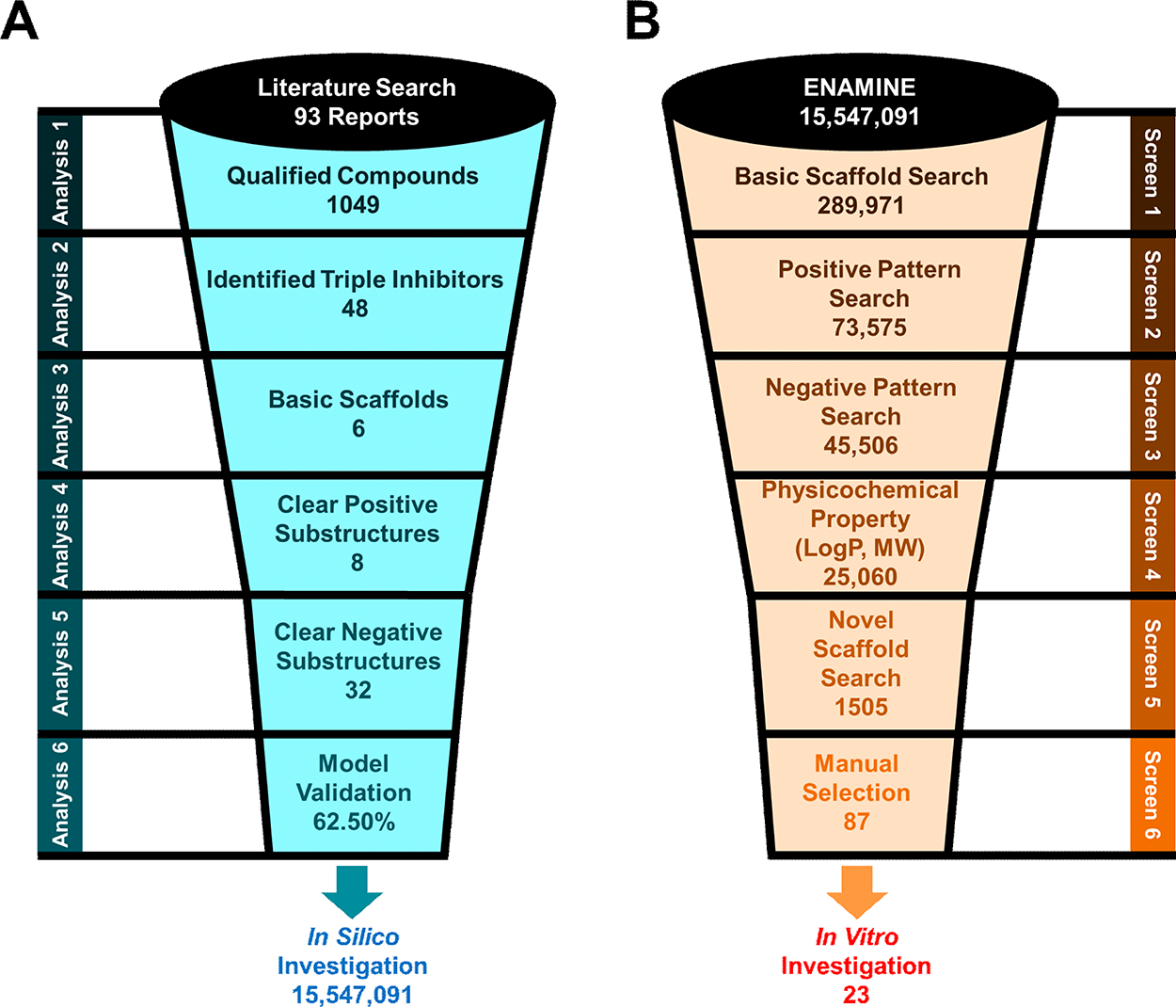
Schematic overview of the process of compound selection. (A) Literature search, data analysis, and development of the computer-aided pattern analysis (C@PA). (B) Screening of a virtual compound library for novel broad-spectrum ABCB1, ABCC1, and ABCG2 inhibitors.

Following the “clear positive hit” search, we defined “clear negative hits” (“Negative Pattern”) that did not account for multitarget inhibition: (i) the respective substructure must have occurred at least five times in the 1049 molecules of the dataset; (ii) the respective substructure did not account for one single triple inhibitor; (iii) the percentage of group B should have been at least the same as the percentage of group C. Respecting these rules, 33 substructures could be found as potential markers for non-triple ABCB1, ABCC1, and ABCG2 inhibition: (i) *tert*-butyl; (ii) vinyl; (iii) cyclopropyl; (iv) cyclohexyl; (v) anellated cyclopropyl; (vi) anellated cycloheptyl; (vii) dimethylamino; (viii) diethylamino; (ix) nitro; (x) pyrrolidine; (xi) methylene hydroxy; (xii) ethylene hydroxy; (xiii) oxolane; (xiv) carboxylic acid; (xv) carboxylic acid methyl ester; (xvi) biphenyl; (xvii) stilbene; (xviii) 1,2,3-triazole; (xix) 1,2,4-triazole; (xx) tetrazole; (xxi) pyrido[2,3-*d*]pyrimidine; (xxii) 1,3-dihydroisobenzofuran; (xxiii) chalcone; (xxiv) hydroquinone; (xxv) 2-methoxyphenyl; (xxvi) 3-methoxyphenyl; (xxvii) 2,5-dimethoxyphenyl; (xxviii) 3,5-dimethoxyphenyl; (xxix) unsubstituted thioamide; (xxx) substituted thioamide; (xxxi) oxazole; (xxxii); urea; and (xxxiii) thiourea. As no thioamide was substituted in the 12 representatives of the 1049 compounds, only the unsubstituted thioamide partial structure has been considered as clear negative hit. Figure 3A visualizes the conducted steps. In summary, the eight identified clear positive hits and 32 clear negative hits form the critical fingerprints for multitarget ABCB1, ABCC1, and ABCG2 inhibition.

#### Model Validation and Comparison to Classical Computational Approaches

**Figure 4:**
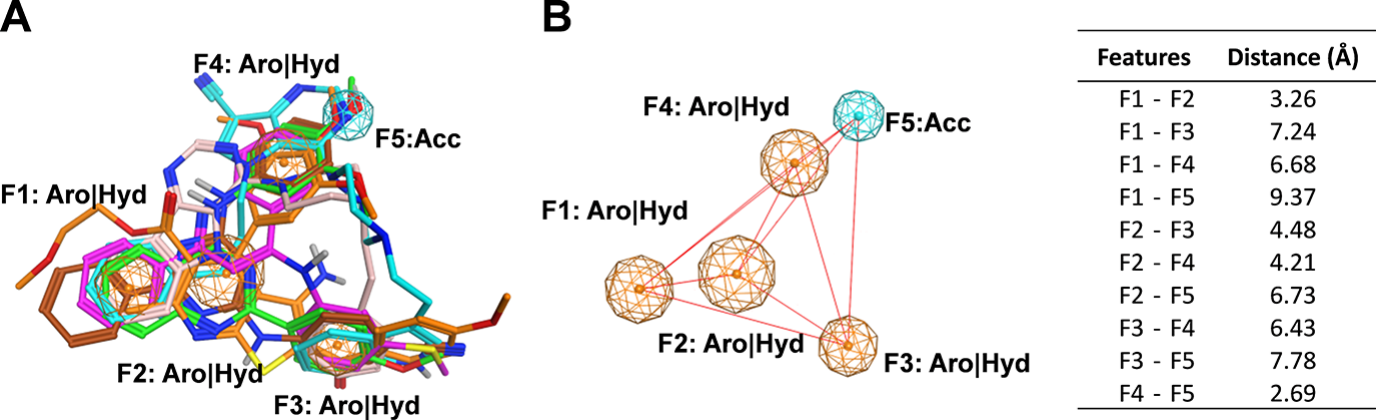
Flexible alignment of the six selected query molecules **1** and **4**–**8** with the five pharmacophore features F1–F5 (F1–F4: aromatic/hydrophobic; and F5: acceptor; A), and the distances between the pharmacophore features are shown in Å as red lines (B), and the distance values can be found in the table to the right.

Before screening of a large virtual compound library, the developed model for compound selection was validated by using a query search tool implemented in InstantJChem.^51^ The 1049 compounds served as a validation data set for “Positive Patterns” (Screen 2) and “Negative Patterns” (Screen 3), which were applied as multitarget fingerprints. Applying these two multitarget fingerprints, 30 of the 48 triple ABCB1, ABCC1, ABCG2 inhibitors could be predicted, while 18 represented false negative hits. This equals a virtual hit rate (“Sensitivity”) of 62.50%, while the prediction of true negatives (“Specificity”) reached 90.81%. To assess the quality and potential superiority of C@PA to classical computational approaches, these results were compared to (i) the 2D similarity search using MACCS fingerprints^16^ and (ii) pharmacophore modeling as already reported before.^16^ For both approaches, six query molecules of every basic scaffold have been chosen: (i) compound **1** as representative of the 4-anilinopyrimidines;^14^ (ii) compound **4** as representative of the pyrrolo[3,2-*d*]pyrimidines;^17^ (iii) compound **5** as representative of the pyrimido[5,4-*b*]indoles;^17^ (iv) compound **6** as representative of the quinazolines;^28^ (v) compound **7** as representative of the quinolines;^29^ and (vi) compound **8** as representative of the thieno[2,3-*b*]pyridines.^32^ The SMILES codes and inhibitory activities of compounds **1** and **4**–**8** can be found in Supplementary Tables 1 and 2. For similarity search, MACCS fingerprints were calculated and a Tanimoto coefficient (Tc) with a cutoff value of 0.8 has been applied. As a result, the sensitivity of this approach yielded in 43.75%, while the specificity reached 87.31%. Regarding the pharmacophore modeling, a flexible alignment of the stated compounds has been performed applying MOE (Figure 4A).^49^ Using the consensus methodology implemented in the Pharmacophore Query Editor, five pharmacophore features [(i–iv) F1–F4: aromatic/hydrophobic; and (v) F5: acceptor] were identified that were present in at least four of the six query molecules **1** and **4**–**8** (tolerance distance: 1.2 Å; threshold value: >50%; Figure 4B). The sensitivity of this approach reached 60.42%, while the specificity had a value of 44.46%. Table 1 gives the prediction values for each class and computational approach. As it turned out, C@PA combined the high sensitivity of the pharmacophore modeling with the high specificity of the similarity search and, moreover, slightly exceeded these values. As its superiority was proven in the process of model validation, we felt confident to continue with large-scale virtual screening.

**Table 1:**
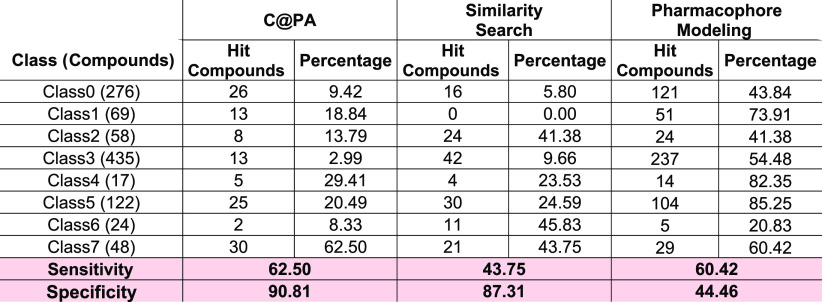
Absolute and Relative Hit Values As Obtained from C@PA Compared to Two Classical Computational Approaches, Similarity Search and Pharmacophore Modeling^a^

a The sensitivity (“virtual hit rate”; true positive hits) as well as the specificity (true negative hits) are highlighted at the very bottom of the table in a rose mark.

#### Virtual Screening, Selection Criteria, and Manual Candidate Selection

For the discovery of novel triple ABCB1, ABCC1, and ABCG2 inhibitors, the ENAMINE Diverse REAL drug-like compound library comprising 15,547,091 molecules was taken for virtual screening.^52^ Three initial selection criteria were formulated: (i) the compound must have contained at least one of the six identified basic scaffolds (Screen 1: “Scaffold Search”; Figure 2); (ii) the compound must have contained at least one of the defined clear positive hits (Screen 2: “Positive Pattern”); and (iii) the compounds must not have been equipped with any of the clear negative hits (Screen 3: “Negative Pattern”). In total, 289,971 compounds had at least one basic scaffold. Amongst these, 73,575 candidates included at least one clear positive hit substructure, while 45,506 of them did not have any clear negative hit substructure. Furthermore, compounds were excluded if they did not have a partition coefficient (LogP) as well as molecular weight (MW) that stretched inside the span of LogP and MW of class 7 compounds (Screen 4; LogP span: 2.4–6.9; MW span: 295–915). This downsized the compound library to 25,060 potential multitarget ABCB1, ABCC1, and ABCG2 inhibitors.

In order to obtain novel agents that had scaffolds not associated with simultaneous inhibition of ABCB1, ABCC1, and ABCG2 before, substructures of Supplementary Table 4 were emphasized that have not been part of any of the 1049 molecules, which was the case for 146 substructures. The focus of this work was to discover new heteroaromatic scaffolds as multitarget ABCB1, ABCC1, and ABCG2 inhibitors. Hence, out of the 146 novel substructures, 29 heteroaromatic scaffolds were chosen: (i) benzopyrazole; (ii) pyrrolo[3,2-*b*]pyridine; (iii) pyrrolo[3,2-*c*]pyridine; (iv) pyrrolo[2,3-*c*]pyridine; (v) carbazole; (vi) phthalazine; (vii) pyrido[3,2-*d*]pyrimidine; (viii) pyrido[4,3-*d*]pyrimidine; (ix) pyrimido[4,5-*d*]pyrimidine; (x) pteridine; (xi) 1,2,3-triazine; (xii) dibenzofuran; (xiii) dibenzothiophene; (xiv)1,2,3-oxadiazole; (xv) 1,2,4-oxadiazole; (xvi) 1,2,5-oxadiazole; (xvii) isothiazole; (xviii) 1,2,3-thiadiazole; (xix) 1,2,4-thiadiazole; (xx) 1,2,5-thiadiazole; (xxi) 1,3,4-thiadiazole; (xxii) furo[3,2-*b*]pyridine; (xxiii) furo[3,2-*c*]pyridine; (xxiv) furo[2,3-*c*]pyridine; (xxv) furo[2,3-*d*]pyrimidine; (xxvi) furo[3,2-*d*]pyrimidine; (xxvii) thieno[3,2-*b*]pyridine; (xviii) thieno[3,2-*c*]pyridine; and (xxix) thieno[2,3-*c*]pyridine. Screening of these 25,060 compounds resulted in 1505 novel heteroaromatic putative ABCB1, ABCC1, and ABCG2 inhibitors (Screen 5: “Novel Scaffold Search”; Supplementary Table 5).

Regarding the basics scaffolds, these 1505 molecules comprised (i) 35 4-anilinopyrimidines, (ii) 0 pyrrolo[3,2-*d*]pyrimidines, (iii) 0 pyrimido[5,4-*b*]indoles, (iv) 232 quinazolines, (v) 1007 quinolines, and (vi) 241 thieno[2,3-*b*]pyridines. With respect to the positive patterns, (i) 531 compounds had an isopropyl residue, (ii) 334 contained an amino group, (iii) 56 were carboxylic acid ethyl esters, (iv) 86 were indoles, (v) 2 possessed a 3,4,5-trimethoxyphenyl partial structure, (vi) 38 possessed a morpholine, (vii) 241 were thieno[2,3-*b*]pyridines, and (viii) 339 comprised a sulfone substructure. From this compilation of candidates, compounds **11**–**33** were assembled through a manual selection. In this manual selection, substituents were in focus that have shown in previous studies to strongly engage the ABC transporter inhibitor with their respective target,^27−29,53,54^*e.g.*, fluorine (**17**–**18**, **21**, **29**–**30**, and **32**), chlorine (**16**–**17**, **23**, **31**, and **33**), cyano (**18**, **26**), or methoxy (**15**, **21**, **24**, **26**, **28**, and **31**), if possible at the main scaffolds and in combination with one another (**17**–**18**, **21**, **26**, **31**). Furthermore, the molecules should be two-centered (**11**–**13**, **18**, **20**, **22**–**23**, **26**, **28**–**30**, and **33**), three-centered (**14**–**17**, **19**, **21**, **24**–**25**, and **31**–**32**), or four-centered (**27**) with linkers of different size connecting each of the (hetero)aromatic centers. Finally, piperazine (**11** and **22**) and piperazine-like linkers [homo-piperazine (**13** and **18**) and piperidine (**26**)] were emphasized since piperazine is often present as a linker in multitarget ABC transporter inhibitors.^16,17,53−55^ In essence, these experience-based decisions as well as availability and price of the compounds led to the selection of 87 candidates, from which 43 were ordered from ENAMINE. Amongst these 43 compounds, 23 were available for delivery within the purity requirement of 95% (compounds **11**–**33**; Supplementary Table 6) and were subject to subsequent biological evaluation. Figure 3B summarizes the virtual screening processes of C@PA.

### Biological Investigation

#### Assessment of Potential Triple ABCB1, ABCC1, and ABCG2 Inhibitors

Compounds **11**–**33** were screened at 5 and 10 μM in calcein AM (ABCB1 and ABCC1) and pheophorbide A (ABCG2) fluorescence accumulation assays. This was performed using either ABCB1-overexpressing A2780/ADR, ABCC1-overexpressing H69AR, or ABCG2-overexpressing MDCK II BCRP cells as reported earlier.^14−16,56^ In short, calcein AM and pheophorbide A are ABC transporter substrates that diffuse into the cells and get effluxed by the respective ABC transporter. In the case of ABC transporter inhibition, the corresponding substrate accumulates inside the cell. Unspecific esterases cleave calcein AM to the fluorescent calcein, which becomes trapped inside the cells because of its free acid groups. In this state, it is easily detectable using a microplate reader. On the other hand, pheophorbide A is already fluorescent and has been evaluated *via* flow cytometry. In both assays, the amount of measured intracellular fluorescence correlated with the degree of inhibition of the respective transporter. Cyclosporine A (10 μM) and Ko143 [(3*S*,6*S*,12a*S*)-1,2,3,4,6,7,12,12a-octahydro-9-methoxy-6-(2-methylpropyl)-1,4-dioxopyrazino[1′,2′:1,6]pyrido[3,4-*b*]indole-3-propanoic acid 1,1-dimethylethyl ester; compound **34**; 10 μM] have been chosen as positive controls for ABCB1 and ABCC1 as well as ABCG2, respectively, defining 100% inhibition.

**Figure 5:**
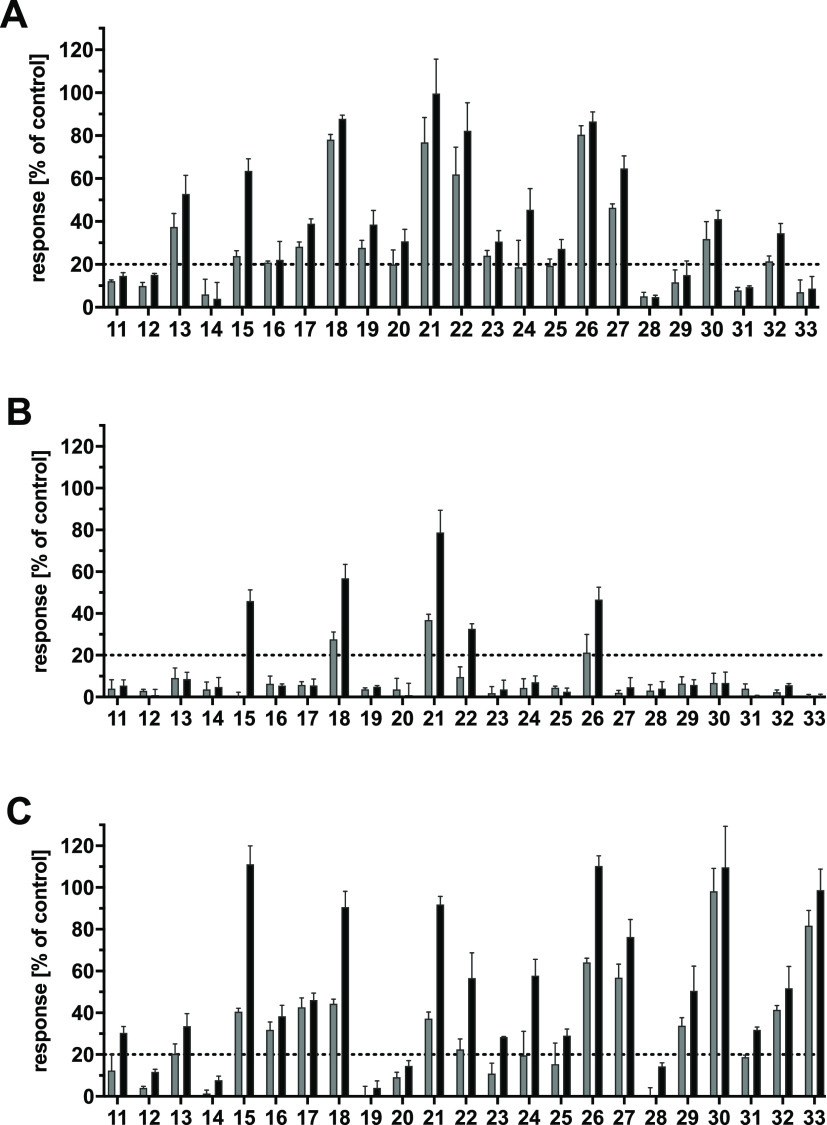
Inhibitory effect of compounds **11**–**33** at 5 μM (grey) and 10 μM (black) against ABCB1 (A), ABCC1 (B), and ABCG2 (C) using either ABCB1-overexpressing A2780/ADR cells (A), ABCC1-overexpressing H69AR cells (B), or ABCG2-overexpressing MDCK II BCRP cells (C) in either calcein AM (A and B) or pheophorbide A (C) assays. Data were normalized by defining the effect of 10 μM cyclosporine A (A and B) and compound **34** (C) as a positive control (100%) and buffer medium as a negative control (0%). Shown is the mean ± standard error of the mean (SEM) of at least three independent experiments.

**Figure 6:**
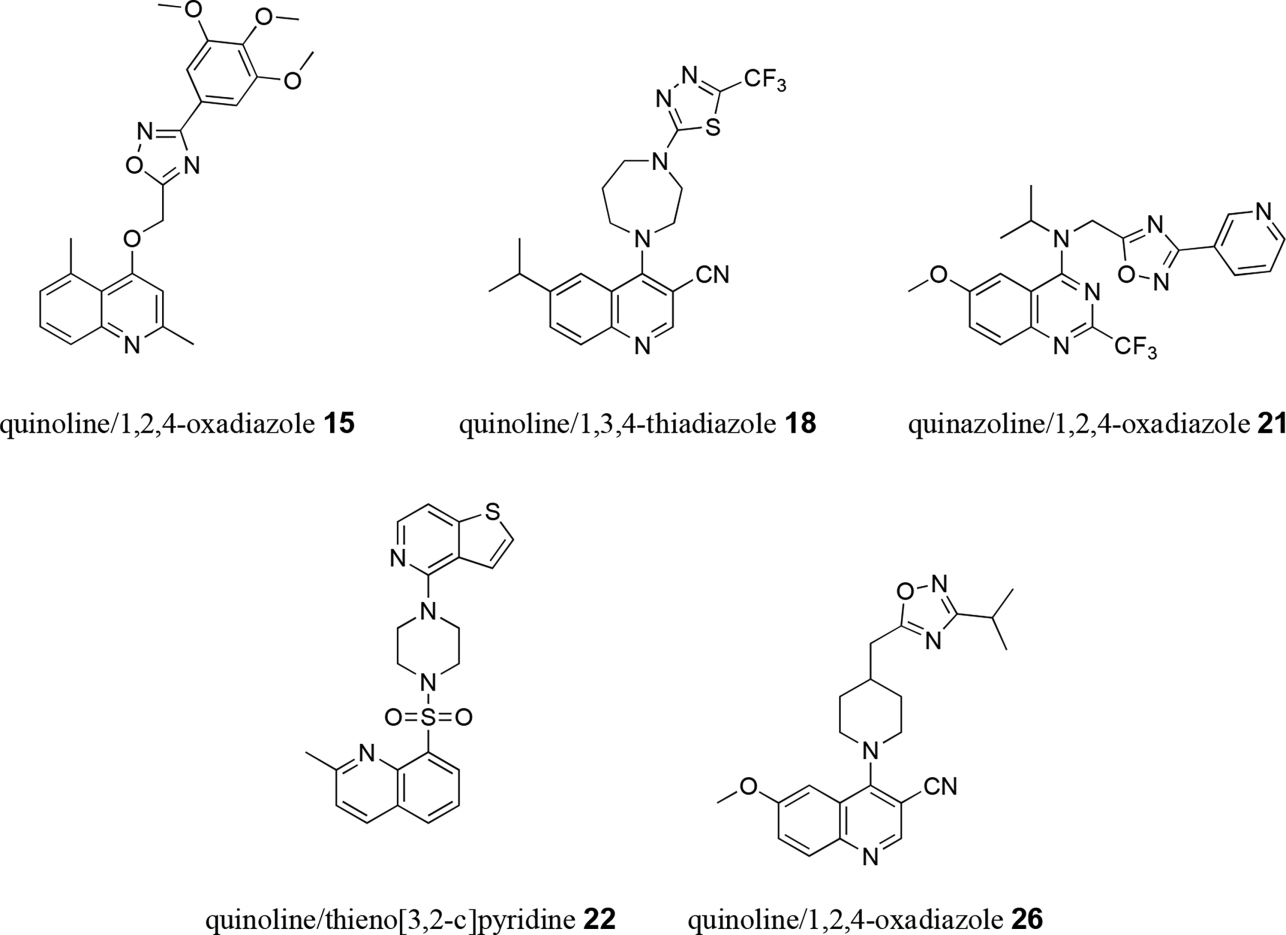
2D representation of multitarget ABCB1, ABCC1, and ABCG2 inhibitors discovered in this work: the quinoline and 1,2,4-oxadiazole derivative **15**, the quinoline and 1,3,4-thiadiazole derivative **18**, the quinazoline and 1,2,4-oxadiazole derivative **21**, the quinoline and thieno[3,2-*c*]pyridine derivative **22**, as well as the quinoline and 1,2,4-oxadiazole derivative **26**.

As can be seen from Figure 5A–C, 17, 5, and 18 of the 23 compounds showed an inhibitory activity against ABCB1 (A), ABCC1 (B), and ABCG2 (C), respectively, of over 20% [+ standard error or the mean (SEM)]. Hence, complete concentration-effect curves have been generated to obtain IC_50_ values for these compounds, which are summarized in Table 2. Compounds **15**, **18**, **21**, **22**, and **26** could be identified as triple ABCB1, ABCC1, and ABCG2 inhibitors and are depicted in Figure 6.

**Table 2:**
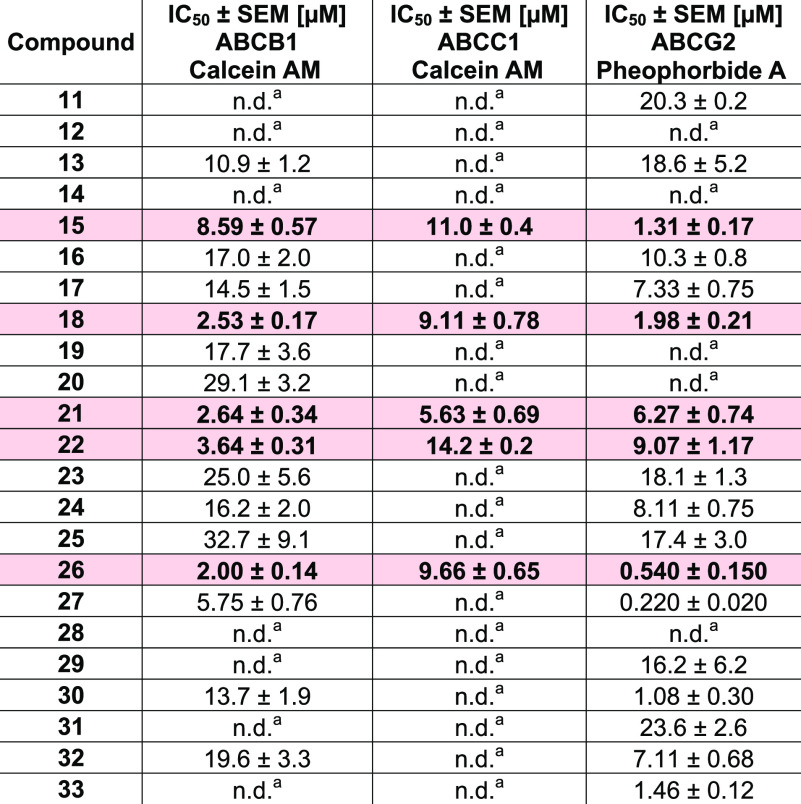
IC_50_ Values of Active Compounds That Had an Inhibition Level of At Least 20% [+ Standard Error of the Mean (SEM)] against ABCB1, ABCC1, and/or ABCG2 in the Initial Screening (Figure 5A–C)#

# ABCB1-overexpressing A2780/ADR, ABCC1-overexpressing H69AR, or ABCG2-overexpressing MDCK II BCRP cells in either calcein AM (ABCB1 and ABCC1) or pheophorbide A (ABCG2) assays were used.^14−16,56^ The positive control (100%) was defined by the effect value of 10 μM cyclosporine A (ABCB1 and ABCC1) or compound **34** (ABCG2), while buffer medium served as a negative control (0%). Shown is the mean ± SEM of at least three independent experiments. Rose mark: discovered triple ABCB1, ABCC1, and ABCG2 inhibitors.

a No IC_50_ determined due to lack of inhibitory activity in the initial screening (Figure 5A–C).

**Figure 7:**
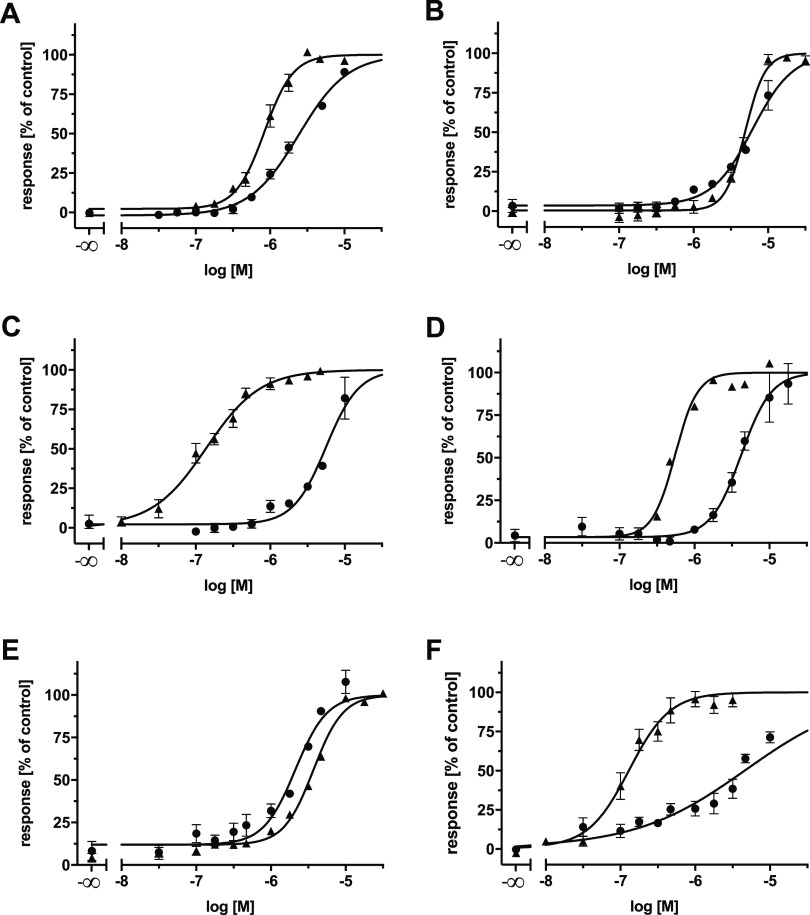
Concentration-effect curves of compound **21** (●) against ABCB1 (A and D), ABCC1 (B and E), and ABCG2 (C and F) as determined in calcein AM (A and B), pheophorbide A (C), Hoechst 33342 (D and F), and daunorubicin (E) assays using ABCB1-overexpressing A2780/ADR cells (A and D), ABCC1-overexpressing H69AR cells (B and E), or ABCG2-overexpressing MDCK II BCRP cells (C and F). Data were normalized by defining the effect of 10 μM cyclosporine A (▲; A, B, D, and E) and compound **34** (▲; C and F) as a positive control (100%) and buffer medium as a negative control (0%). Shown is the mean ± SEM of at least three independent experiments.

The most potent representative, compound **21**, had IC_50_ values of 2.64, 5.63, and 6.27 μM against ABCB1, ABCC1, and ABCG2, respectively. This makes compound **21** belonging to the around 50 most potent multitarget ABCB1, ABCC1, and ABCG2 inhibitors,^14−17,21,23,25−42^ which is also true for compounds **18** and **26**. Figure 7A–C shows the concentration-effect curves of compound **21**, while Supplementary Figures 1A–C, 2A–C, 3A–C, and 4A–C show the concentration-effect curves of compounds **15**, **18**, **22**, and **26**, respectively, obtained in the calcein AM and pheophorbide A assays. Considering the 23 evaluated compounds, the finding of five multitarget ABCB1, ABCC1, and ABCG2 inhibitors represents a biological hit rate of 21.7%.

**Figure 8:**
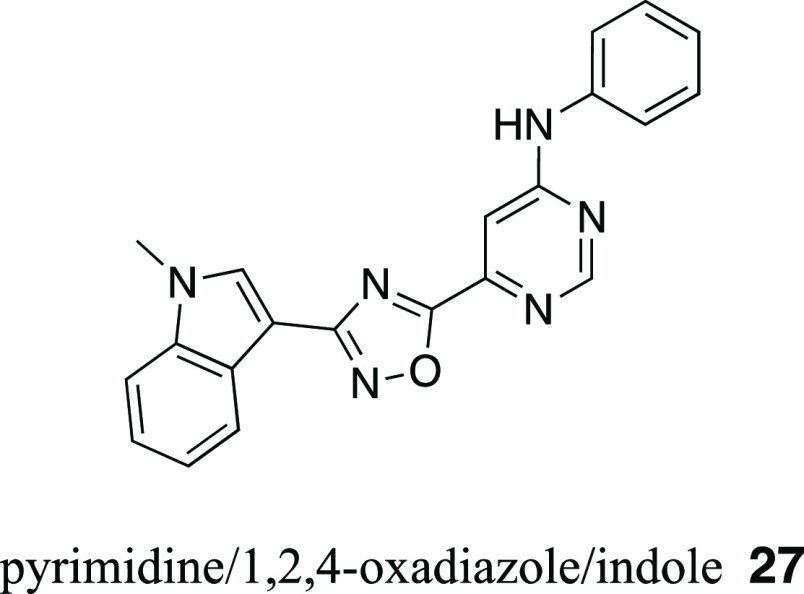
Molecular formula of the very potent ABCG2 inhibitor **27** (IC_50 Pheophorbide A_ = 0.220 μM; IC_50 Hoechst 33342_ = 0.260 μM) as discovered in the herein presented virtual screening approach.

In addition, two compounds revealed a remarkable inhibitory power against ABCG2: the quinoline/1,2,4-oxadiazole/indole derivative **26** (IC_50_ = 0.540 ± 0.150 μM; Figure 7) and the pyrimidine/1,2,4-oxadiazole/indole derivative **27** (IC_50_ = 0.220 ± 0.020 μM; Figure 8). This is a special finding given the fact that screenings usually do not provide compounds with very high activities. Especially, the results for compound **27** must be put into perspective as it possessed an equal inhibitory power against ABCG2 as the “golden standard”, compound **34**. Hence, it represents a promising lead molecule for ongoing research.

#### Confirmation of Inhibitory Power of Compounds **15**, **18**, **21**, **22**, **26**, and **27**

In order to confirm the found results with respect to multitarget ABCB1, ABCC1, and ABCG2 inhibition of compounds **15**, **18**, **21**, **22**, and **26** as well as ABCG2 inhibition of compound **27**, Hoechst 33342 (ABCB1 and ABCG2),^15,57^ and daunorubicin (ABCC1)^17^ fluorescence accumulation assays have been performed as described previously^15,17,57^ with minor modifications, using either ABCB1-overexpressing A2780/ADR, ABCC1-overexpressing H69AR, or ABCG2-overexpressing MDCK II BCRP cells. In short, Hoechst 33342 and daunorubicin are substrates of ABC transporters that passively diffuse into the cells and become extruded by the respective ABC transporter. ABC transporter inhibition leads to an intracellular accumulation of these fluorescence dyes. Hoechst 33342 intercalates with the DNA in the nucleus and accumulates in intracellular membrane bilayers, both leading to a fluorescent complex that could be detected using a microplate reader. On the other hand, daunorubicin is already fluorescent and has been evaluated *via* flow cytometry. In both assays, the measured fluorescence values correlated with the degree of inhibition of the respective transporter. Ten micromolar cyclosporine A and compound **34** have been used as references to define 100% inhibition of ABCB1 and ABCC1 as well as ABCG2, respectively. The data for the multitarget ABCB1, ABCC1, and ABCG2 inhibitors **15**, **18**, **21**, **22**, and **26** are summarized in Table 3.

**Table 3:**
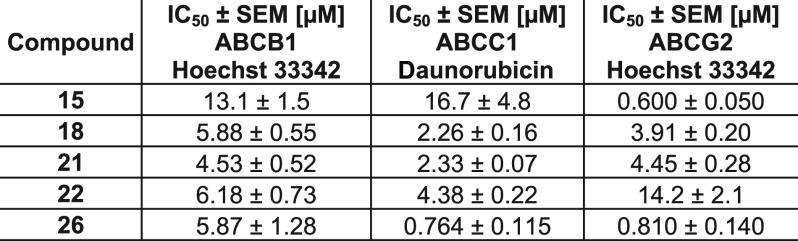
Confirmation of IC_50_ Values of Compounds **15**, **18**, **21**, **22**, and **26**^a^

a Hoechst 33342 and daunorubicin assays were conducted using either ABCB1-overexpressing A2780/ADR, ABCC1-overexpressing H69AR, or ABCG2-overexpressing MDCK II BCRP cells as reported earlier.^15,17,57^ Cyclosporine A (10 μM; ABCB1 and ABCC1) and compound **34** (10 μM; ABCG2) were used as a reference for 100% inhibition, and buffer medium represented 0%. Shown is the mean ± SEM of at least three independent experiments.

Compounds **15**, **18**, **21**, **22**, and **26** could be confirmed as triple ABCB1, ABCC1, and ABCG2 inhibitors. Generally, the IC_50_ values correlated with the values of the calcein AM (ABCB1 and ABCC1) and pheophorbide A (ABCG2) assays (Table 2). Only the IC_50_ value of compound **26** determined in the daunorubicin assay (ABCC1) fell out of the correlation, which was with 0.764 μM over 12 times lower than could have been expected from the calcein AM data. However, these discrepancies frequently occur as IC_50_ values generally depend on the used fluorescence dye, in particular its polarity, lipophilicity, (velocity of) membrane distribution, and affinity to the respective transporter.^15,24,58^ Again, compound **21** was the most potent representative of the five triple inhibitors with IC_50_ values of 4.53, 2.33, and 4.45 μM against ABCB1, ABCC1, and ABCG2, respectively. Considering these values, compound **21** belongs even to the 23 most potent multitarget inhibitors of ABCB1, ABCC1, and ABCG2.^14,15,21,23,25,26,28,32,34,37−39^ The corresponding concentration-effect curves derived in the Hoechst 33342 and daunorubicin assays are shown in Figure 7D–F, while the dose–response curves of compounds **15**, **18**, **22**, and **26** determined in the very same assays are depicted in Supplementary Figures 1D–F, 2D–F, 3D–F, and 4D–F. Additionally, the high inhibitory power of compound **27** could be confirmed as it had an IC_50_ value of 0.260 μM in the Hoechst 33342 assay. In all, the results of the calcein AM and pheophorbide A assays could be confirmed, which finally gave proof that critical fingerprints have been identified to predict multitarget ABC transporter inhibitors by C@PA.

## Discussion and Conclusions

C@PA was a major success, as a prediction of multitarget ABC transporter inhibitors has never been postulated and biologically proven before. More strikingly, compounds **15**, **18**, **21**, **22**, and **26** belong to the structural classes of 1,2,4-oxadiazoles, 1,3,4-thiadiazoles, and thieno[3,2-*c*]pyridines. While 1,2,5-oxadiazoles^59−62^ have frequently been reported as (selective^59−62^ or dual^62^) ABCB1^59,60,62^ and ABCC1 inhibitors,^61,62^ 1,2,4-oxadiazoles^63−65^ have only once been reported as selective ABCG2 inhibitors^65^ or reversers of ABCB1-, ABCC1-, or ABCG2-mediated MDR.^63,64^ 1,3,4-Thiadiazoles have also only once been reported in association with selective, dual, or triple ABCB1, ABCC1, and ABCG2 inhibition.^66^ However, these compounds had estimated IC_50_ values of far beyond 25 μM.^66^ Thieno[3,2-*c*]pyridines, on the other hand, have never been associated with either ABCB1, ABCC1, or ABCG2.

A biological hit rate of 21.7% is common for single-target computational approaches as reported in the literature that subsequently validated their postulations with biological studies.^67−71^ However, this number is very impressive for multitarget screening studies, in particular considering the huge challenges involved in the development of C@PA. We identified four major aspects that impacted the model development.

The first aspect is related to the selection of molecules as basis for the development of C@PA. The amount of data that could be used was highly limited. We found only 93 reports containing 1049 compounds that qualified for data processing. Many compounds were not characterized in full by complete concentration-effect curves and had to be estimated for a proper compound categorization and classification. The data processing procedures in these 93 reports that led to the published IC_50_ values were not standardized (*e.g.*, three- vs four-parameter logistic equation). Some IC_50_ values provided a limited number of significant digits or were not accompanied by standard deviations or standard errors. Furthermore, certain IC_50_ values resulted from so-called “partial inhibitors” (IC_50 absolute_ vs IC_50 relative_). Additionally, the applied assay systems were not standardized and varied within the 93 reports. While a majority of testing systems was accumulation (uptake) assays, some findings were based on efflux experiments. Furthermore, the transporter host system varied in the reported biological studies. While the majority of authors used living cells, some used inside-out membrane vesicles, both for its part influencing compound distribution and binding, but also transporter abundance and functionality (*e.g.*, pump rate).^24^ The living cells for their part were either transfected or selected cells, which impacted the expression and abundance of (functional) transport protein and eventually the inhibitory activity against the respective transporter. More importantly, a great variety of fluorescence dyes has been used to assess the corresponding compounds. It is well known that inhibitory activity can be strongly dependent on the manner of the fluorescence dye [*e.g.*, its polarity, lipophilicity, velocity of diffusion and distribution, as well as affinity toward the transporter(s)].^15,24,58^ Moreover, fluorescence measurements themselves pose a risk of artifacts, which can be explained by secondary effects like quenching (with each other or with the evaluated compounds). This can be circumvented by the use of other types of measurements, like radioactivity counts in radionuclide studies. However, this kind of testing system has only been used by a minority of authors. Finally, it must be taken into consideration that the 93 reports came from various laboratories with different non-standardized assay procedures, resulting in the very same assay being executed in various manners. Taken these data-related aspects together, the errors of each individual aspect collectively potentiated, giving a final uncertainty for C@PA’s prediction capabilities.

The second major aspect stems from the initial categorization of compounds into “active” and “inactive”. The “activity threshold” has been set to 10 μM. A threshold in general adversely affects compounds close to the chosen value, which inevitably leads to misclassifications. However, only 19 (ABCB1), 24 (ABCC1), and 42 (ABCG2) so-called “borderline-compounds”, where the IC_50_ ± SD/SEM values either overlapped with the threshold of 10 μM, or were defined as “around 10 μM” (∼10) or exactly 10 μM (10.000), have been identified from Supplementary Table 1. Hence, the problem of miscategorization of the compounds is rather negligible. Although the value of 10 μM seems to be quite high, one must take into account that broad-spectrum ABCB1, ABCC1, and ABCG2 inhibitors generally exert their effect almost always in the single- to double-digit micromolar concentration range. As stated out in the Introduction, only about 50 triple ABCB1, ABCC1, and ABCG2 inhibitors exerted their effect below 10 μM,^14−17,21,23,25−42^ and only 22 of them had activities below 5 μM.^14,15,21,23,25,26,28,32,34,37−39^ Setting the threshold to higher activities (lower IC_50_ values) would have led to a radical downsizing of the data set. This would not have left enough space for action and interpretation. Even setting the threshold at 10 μM allowed only for 48 triple ABCB1, ABCC1, and ABCG2 inhibitors to be considered as a basis of scaffold analysis and the following computational measures. A higher threshold would have led to a larger number of triple ABCB1, ABCC1, and ABCG2 inhibitors, but this would have led to the inclusion of rather inactive compounds. In addition, IC_50_ values above 10 μM imply that the necessary test concentrations were much higher (up to 100 μM or more). At these concentration ranges, compound-related assay interferences (*e.g.*, solubility problems, solvent effects, short-term cell toxicity, fluorescence quenching, or unspecific binding) are much more likely to have occurred. Hence, compounds with activities above 10 μM could not be considered as “active”. However, it must also be stated that, due to the 10 μM threshold, the value distribution after compound categorization and classification was rather unequal. This can be seen, for example, when comparing selective ABCG2 inhibitors (class 3) with 435 representatives, and dual ABCB1 and ABCC1 inhibitors (class 4) with 17 compounds. This mainly depended on the literature itself and could not be influenced.

The third major aspect was the data processing and the definition of selection criteria. A virtual hit rate of 62.5% is above average; however, the model was not able to predict all 48 triple ABCB1, ABCC1, and ABCG2 inhibitors, although its selection criteria were in part deduced from these. In terms of the selection criteria, it must generally be considered that selectivity and promiscuity are not discrete but continuous attributes of compounds. Statistically speaking, there is a fluent border between both attributes. Molecules consist mostly of several partial structures that for their part can independently or collectively interact with the target(s) leading to selectivity or promiscuity. This ambivalent characteristic of individual substructures can lead to the fact that these are present in both single- or multitarget inhibitors. Our aim was to define unambiguous selection criteria, or at least as close to this as possible. This explains why many substructures present in the triple inhibitors could not be acknowledged for the prediction of the very same triple inhibitors from the data set of 1049 compounds. Inclusion of these discriminated partial structures would inevitably have led to the prediction of more false positive hits and a decreased biological hit rate. To avoid a “randomization” of the model, we chose 15% as the threshold for the selection of clear positive hits. This threshold allowed for the selection of a sufficient number of substructures as clear positive hits. A higher percentage almost eliminates these clear positive hits, while a lower percentage results in the selection of less pronounced multitarget substructures (leading to more false positive hits). On the other hand, this number of 15% implies that the residual 85% of molecules contained dually active, selective, or even inactive compounds. This imbalance posed in our point of view the highest impact on the development of C@PA. Furthermore, novel scaffolds (Screen 5) were chosen that have never been reported before regarding the ABC transporters ABCB1, ABCC1, and ABCG2 according to the initial data set of 1049 compounds (Supplementary Table 4). Selecting for these 29 novel heteroaromatic scaffolds inherited *per se* a risk of lowering the biological hit rate. However, as the task of this investigation was to identify novel heteroaromatic scaffolds and molecules, stepping into this unknown territory was obligatory. Finally, the manual selection posed also a risk of faulty selection. As outlined above, these criteria were mainly based on our experience with ABC transporter inhibitors.^16,17,27−29,55^ C@PA benefited from these experience-driven decisions, as the following shows: (i) a strong focus was put on individual substituents like fluorine, chlorine, cyano, or methoxy, especially in combination. Strikingly, 6 of the 23 compounds had such a combination (**17**–**18**, **21**, **26**, **31**, and **33**), amongst these were three triple ABCB1, ABCC1, and ABCG2 inhibitors (**18**, **21**, and **26**; 50.0%). More strikingly, almost all (**15**, **18**, **21**, and **26**; 80.0%) of the triple inhibitors had at least one of such a substructure. Moreover, when turning the focus on dual and triple (= multitarget) inhibitors of ABC transporters, 71.4% (10 out of 14) had at least one of these substructures; (ii) the partial structures piperazine (**22**), homo-piperazine (**18**), and piperidine (**26**) were reflected in the five multitarget ABCB1, ABCC1, and ABCG2 inhibitors (60.0%); Hence, we conclude that the manual selection rather supported than impaired the model and contributed to the finding of multitarget ABCB1, ABCC1, and ABCG2 inhibitors.

The fourth and final major aspect is the target variety. Multitarget inhibition was in the focus of the present study. As ABCB1,^2^ ABCC1,^3^ and ABCG2^4^ have their individual “preferences” regarding inhibitors, finding simultaneously interfering agents is quite an obstacle, which distinguishes C@PA from other approaches in the literature.^67−71^ Compound characteristics such as lipophilicity or MW can inversely correlate with the inhibition of the respective transporter, therefore exacerbating the finding of a multitarget inhibitor. This raised initially the question if a rational approach was possible at all to obtain novel multitarget ABCB1, ABCC1, and ABCG2 inhibitors.

Despite these multifaceted challenges, the model proved that it is generally possible to predict broad-spectrum ABCB1, ABCC1, and ABCG2 inhibitors after processing of literature data and identification of critical fingerprints. This cannot only be perceived from the finding of five novel multitarget ABCB1, ABCC1, and ABCG2 inhibitors but also from the discovery of nine dual ABCB1 and ABCG2 inhibitors (**13**, **16**, **17**, **23**–**25**, **27**, **30**, and **32**). Consequently, 60.9% of the selected 23 molecules were multitarget inhibitors of ABC transporters. Although dual inhibition was not in the scope of the present study, it must be acknowledged that these numbers mean that suitable molecular patterns were extracted for multitarget ABCB1, ABCC1, and ABCG2 inhibition. In addition, two major achievements are that (i) the 1,2,4-oxadiazole moiety can be suggested as the seventh basic scaffold for triple ABCB1, ABCC1, and ABCG2 inhibition, and (ii) the fluorine, chlorine, methoxy, as well as cyano substructures, as well as the piperazine, homopiperazine, and piperidine linkers can, in association with multitarget ABC transporter inhibition, at least be considered as “secondary positive patterns”. Both achievements complement the multitarget fingerprints and will be of use when improving C@PA’s prediction capabilities (e.g., as C@PA_1.2).

C@PA provides the unique opportunity to shift the methodology to discover multitarget ABCB1, ABCC1, and ABCG2 inhibitors from “serendipity” to “rationale”. Now, it is not a matter of luck anymore to gain novel multitarget inhibitors, but only of statistics, and C@PA proved also to be greatly efficient compared to other computational approaches, such as similarity search and pharmacophore modeling. Remarkably, considering that common motifs within the ABC transporter superfamily exist, C@PA provides also the unique chance to predict and discover novel agents that target understudied ABC transporters that cannot be addressed by small-molecules until now. Finally, this methodology may be transferred to other protein families as well, thriving also drug development in other scientific areas in general.

## Experimental Section

### Computational Analysis

#### Compilation of Data Set and Categorization of Compounds

Literature research to find and assemble inactive, selective, dual, and triple inhibitors of the ABC transport proteins ABCB1, ABCC1, and ABCG2 was conducted using the National Center for Biotechnological Information (NCBI).^72^ Reports were only considered when they either presented simultaneous testing at ABCB1, ABCC1, and ABCG2, or the respective compound has been evaluated regarding ABCB1, ABCC1, and ABCG2 in several individual reports. SMILES codes (isomeric if applicable) were either obtained from PubChem,^72^ manually assembled from associated content and supplementary material as provided by the respective report, or manually drawn according to the 2D representations provided by the corresponding report using ChemDraw Pro [version 17.1.0.105 (19)]. Determined IC_50_ values and deviations were assembled as reported in the respective literature under referral to the applied testing system (detection method and host system; Supplementary Table 1). In case the IC_50_ was needed to be estimated from relative inhibition data, the used concentration of the respective compound and its relative effect to a standard ABCB1, ABCC1, and ABCG2 inhibitor were taken into account to categorize the corresponding compound into “active” (estimated IC_50_ value <10 μM) or “inactive” (estimated IC_50_ value ≥10 μM). In total, 1049 compounds from 93 reports between 2004 and 2020 were taken into account for further data processing. The associated original literature is also provided in Supplementary Table 1. For compound categorization, the assembled data in Supplementary Table 1 has been fused to associate one compound with one single IC_50_ value (Supplementary Table 2). In the case of two reported IC_50_ values or a given IC_50_ span, the mean was calculated. In case of defined and estimated IC_50_ values, the defined value has been given priority. In the case of activity (IC_50_ present) and inactivity (IC_50_ not present), the defined IC_50_ value was given priority. Compounds with IC_50_ values below 10 μM were considered as active (1, “one”), others as inactive (0, “zero”). The data provided in Supplementary Table 2 was translated into a script (ABCB1, ABCC1, and ABCG2), and the compounds classified as follows: (i) class 0: 0, 0, 0; (ii) class 1: 1, 0, 0; (iii) class 2: 0, 1, 0; (iv) class 3: 0, 0, 1; (v) class 4: 1, 1, 0; (vi) class 5: 1, 0, 1; (vii) class 6: 0, 1, 1; (viii) class 7: 1, 1, 1 (Supplementary Table 3).

### Basic Scaffold Search and Statistical Substructure Analysis

The Structure-Activity-Report (SAReport) tool^48^ implemented in Molecular Operating Environment (MOE; version 2019.1)^49^ was used for the elucidation of the basic scaffolds of class 7 compounds. A total of 308 substructures^50^ (Supplementary Table 4) were searched amongst the 1049 compounds using InstantJChem,^51^ and their absolute as well as relative distribution was calculated. The relative distribution was categorized into: (i) group A: percentage of class 0; (ii) group B: sum of percentages of classes 1–3; (iii) group C: sum of percentages of classes 4–6; (iv) group D: percentage of class 7; and (v) group E: sum of percentages of classes 4–7.

#### Identification of Multitarget Fingerprints

“Clear positive hits” as indicators for triple ABCB1, ABCC1, and ABCG2 inhibition were defined as follows: (i) the respective substructure must have appeared at least five times within the 1049 molecules; (ii) group D must be at least 15%; and either (iii) group D must be at least equal to group B, or (iv) group E must be at least equal to group B. “Clear negative hits” were defined as follows: (i) the respective substructure must have appeared at least five times amongst the 1049 molecules; (ii) the respective substructure must not account for class 7 compounds (group D must be 0%); and (iii) group B must be at least equal to group C.

#### Model Validation and Comparison to Common Computational Approaches

Model validation for C@PA has been conducted by applying Screen 2 (“Positive Pattern”) and Screen 3 (“Negative Pattern”) using a query search tool implemented in InstantJChem.^51^ The 2D similarity search was performed by using the MACCS fingerprints as implemented in MOE.^49^ This MACCS fingerprint contains 166 structural keys indicating the presence of specified structural fragments in the molecular graph representation. The similarity between the six selected query inhibitors **1** and **4**–**8** as well as the 1049 molecules in the dataset was measured by using a Tanimoto coefficient (Tc) with a cutoff value of 0.8. For the pharmacophore model, the six selected query inhibitors were aligned using the flexible alignment tool implemented in MOE^49^ as described before in detail.^16^ The best alignment was used to generate the pharmacophore model using the consensus methodology implemented in the Pharmacophore Query Editor. In total, 196,439 conformers for the 1049 molecules in the dataset were generated using the conformational search tool in MOE^49^ by applying the stochastic search method with a conformation limit of 10,000. The threshold for the identification of multitarget pharmacophore features was set at 50.0% and a tolerance value of 1.2.

#### Virtual Screening, Selection Criteria, and Manual Candidate Selection

The ENAMINE Diverse REAL drug-like database was downloaded^52^ and screened for compounds with (i) at least one basic scaffold, (ii) at least one clear positive hit, (iii) no clear negative hit, (iv) a LogP and MW that stretched inside the span of class 7 compounds (LogP span: 2.4–6.9; MW span: 295–915), and (v) at least one “novel scaffold”. LogP and MW were calculated using MOE (version 2019.01).^49^ In total 1505 potential candidates resulted, from which 87 were manually selected by experience-driven decisions depending on availability and price, from which 41 were ordered from ENAMINE and 23 were delivered within the purity requirement of 95%. All compounds were screened for substructures present in pan-assay interference compounds (PAINS) and did not contain any of these motifs.^74^

The identities of compounds **11**–**14**, **16**–**19**, and **21**–**32** were determined by ENAMINE *via*^1^H NMR spectroscopy. Compounds **15**, **20**, and **33** were analyzed in our laboratory by using a Bruker Avance 500 MHz (500 MHz). All NMR spectra were recorded in DMSO-*d*_6_, and chemical shifts (δ) are expressed in ppm calibrated to the solvent signal of DMSO (δ: 2.50 ppm). Spin multiplicities of compounds **11**–**33** are given as singlet (s), doublet (d), doublet of doublets (dd), doublet of triplets (dt), and multiplet (m). The purity of compounds **11**–**33** was determined by ENAMINE *via* LC-MS analysis and stated as at least 96% pure. The complete analytical assessment of the compounds is provided in the Supporting Information.

#### 3-(4-{3,4-Dimethylthieno[2,3-*b*]pyridine-2-carbonyl}piperazine-1-carbonyl)-2*H*-indazole (**11**)

ENAMINE ID: Z1001807112; ^1^H NMR (600 MHz, DMSO-*d*_6_) δ: 13.55 (s, 1H), 8.15–8.11 (m, 1H), 8.00–7.98 (m, 1H), 7.63–7.58 (m, 1H), 7.43–7.36 (m, 1H), 7.24–7.20 (m, 1H), 4.30–3.40 (m, 8H), 2.60 (s, 3H), 2.35 (s, 3H); LC-MS (*m*/*z*) calculated for C_22_H_21_N_5_O_2_S: 419.14; found: 420.0 [M + 1]^+^; purity: 100%.

#### 6-Methyl-*N*-[2-(5-propyl-1,2,4-oxadiazol-3-yl)propan-2-yl]quinoline-5-sulfonamide (**12**)

ENAMINE ID: Z1137670336; ^1^H NMR (600 MHz, DMSO-*d*_6_) δ: 9.25–9.20 (m, 1H), 8.90–8.80 (m, 1H), 8.61 (s, 1H), 8.10–8.05 (m, 1H), 7.65–7.55 (m, 2H), 2.73 (s, 3H), 2.48–2.43 (m, 3H), 1.46 (s, 6H), 1.43–1.35 (m, 2H), 0.85–0.78 (m, 3H); LC-MS (*m*/*z*) calculated for C_18_H_22_N_4_O_3_S: 374.14; found: 375.0 [M + 1]^+^; purity: 100%.

#### 3-{[4-(3-Methyl-1,2,4-thiadiazol-5-yl)-1,4-diazepan-1-yl]methyl}-2-(morpholin-4-yl)quinoline (**13**)

ENAMINE ID: Z1569466770; ^1^H NMR (500 MHz, DMSO-*d*_6_) δ: 8.11 (s, 1H), 7.85–7.69 (m, 2H), 7.61–7.58 (m, 1H), 7.41–7.35 (m, 1H), 4.25–3.40 (m, 10H), 3.25–3.08 (m, 4H), 2.90–2.78 (m, 2H), 2.71–2.64 (m, 2H), 2.30–2.15 (s, 3H), 1.98–1.78 (m, 2H); LC-MS (*m*/*z*) calculated for C_22_H_28_N_6_OS: 424.20; found: 425.2 [M + 1]^+^; purity: 100%.

#### 3,4-Dimethyl-*N*-{4-[(1,3,4-thiadiazol-2-yl)sulfamoyl]phenyl}thieno[2,3-*b*]pyridine-2-carboxamide (**14**)

ENAMINE ID: Z1619753040; ^1^H NMR (500 MHz, DMSO-*d*_6_) δ: 14.28 (s, 1H), 10.61 (s, 1H), 8.70 (s, 1H), 8.29–8.20 (m, 1H), 7.95–7.71 (m, 4H), 7.45–7.35 (m, 1H), 2.66–2.53 (m, 6H); LC-MS (*m*/*z*) calculated for C_18_H_15_N_5_O_3_S_3_: 445.03; found: 446.0 [M + 1]^+^; purity: 98%.

#### 2,5-Dimethyl-4-{[3-(3,4,5-trimethoxyphenyl)-1,2,4-oxadiazol-5-yl]methoxy}quinoline (**15**)

ENAMINE ID: Z1815536867; ^1^H NMR (500 MHz, DMSO-*d*_6_) δ: 7.69 (d, *J* = 8.4 Hz, 1H), 7.53 (dd, *J* = 8.4, 7.1 Hz, 1H), 7.30 (s, 2H), 7.26 (dt, *J* = 7.0, 1.2 Hz, 1H), 7.07 (s, 1H), 5.79 (s, 2H), 3.86 (s, 6H), 3.74 (s, 3H), 2,86 (s, 3H), 2.58 (s, 3H); LC-MS (*m*/*z*) calculated for C_23_H_23_N_3_O_5_: 421.16; found: 422.2 [M + 1]^+^; purity: 96%.

#### 5-Chloro-*N*-{[3-(4-methylquinolin-2-yl)-1,2,4-oxadiazol-5-yl]methyl}thiophene-2-sulfonamide (**16**)

ENAMINE ID: Z1890912753; ^1^H NMR (600 MHz, DMSO-*d*_6_) δ: 9.19 (s, 1H), 8.23–8.11 (m, 2H), 7.96 (s, 1H), 7.90–7.84 (m, 1H), 7.78–7.73 (m, 1H), 7.54–7.50 (m, 1H), 7.23–7.18 (m, 1H), 4.60 (s, 2H), 2.80 (s, 3H); LC-MS (*m*/*z*) calculated for C_17_H_13_ClN_4_O_3_S_2_: 420.01; found: 421.0 [M + 1]^+^; purity: 100%.

#### 3-[(5-Chloro-1,3-dimethyl-1*H*-pyrazol-4-yl)methyl]-5-[3-methyl-6-(trifluoromethyl)thieno[2,3-*b*]pyridin-2-yl]-1,2,4-oxadiazole (**17**)

ENAMINE ID: Z1891639106; ^1^H NMR (600 MHz, DMSO-*d*_6_) δ: 8.80–8.75 (m, 1H), 8.10–8.05 (m, 1H), 7.96 (s, 1H), 3.95 (s, 2H), 3.70 (s, 3H), 2.85 (s, 3H), 2.16 (s, 3H); LC-MS (*m*/*z*) calculated for C_17_H_13_ClF_3_N_5_OS: 427.05; found: 428.0 [M + 1]^+^; purity: 98%.

#### 6-(Propan-2-yl)-4-{4-[5-(trifluoromethyl)-1,3,4-thiadiazol-2-yl]-1,4-diazepan-1-yl}quinoline-3-carbonitrile (**18**)

ENAMINE ID: Z1896692207; ^1^H NMR (600 MHz, DMSO-*d*_6_) δ: 8.80 (s, 1H), 7.98–7.93 (m, 1H), 7.80–7.75 (m, 2H), 4.08–4.00 (m, 2H), 3.96–3.83 (m, 4H), 3.68–3.63 (m, 2H), 3.05–2.95 (m, 1H), 2.25–2.18 (m, 2H), 1.25–1.18 (m, 6H); LC-MS (*m*/*z*) calculated for C_21_H_21_F_3_N_6_S: 446.15; found: 447.0 [M + 1]^+^; purity: 100%.

#### 3-[3-(Furan-3-yl)-1,2,4-oxadiazol-5-yl]-*N*-(propan-2-yl)-*N*-(quinolin-3-yl)propanamide (**19**)

ENAMINE ID: Z1933909500; ^1^H NMR (600 MHz, DMSO-*d*_6_) δ: 8.80 (s, 1H), 8.43–8.33 (m, 2H), 8.13–8.05 (m, 2H), 7.90–7.80 (m, 2H), 7.73–7.65 (m, 1H), 6.88 (s, 1H), 4.95–4.85 (m, 1H), 3.13–3.03 (m, 2H), 2.45–2.35 (m, 2H), 1.25–0.80 (m, 6H); LC-MS (*m*/*z*) calculated for C_21_H_20_N_4_O_3_: 376.15; found: 377.0 [M + 1]^+^; purity: 100%.

#### *N*-Methyl-*N*-(quinolin-8-yl)thieno[3,2-*b*]pyridine-6-sulfonamide (**20**)

ENAMINE ID: Z1990107654; ^1^H NMR (500 MHz, DMSO-*d*_6_) δ: 8.83 (dd, *J* = 2.1, 0.8 Hz, 1H), 8.71 (d, *J* = 2.1 Hz, 2H), 8.43 (d, *J* = 5.5 Hz, 2H), 8.37 (dd, *J* = 8.3, 1.7 Hz, 1H), 8.28 (dd, *J* = 4.1, 1.7 Hz, 1H), 8.02 (dd, *J* = 8.2, 1.4 Hz, 1H), 7.77 (dd, *J* = 7.4, 1.4 Hz, 1H), 7.67 (dd, *J* = 5.5, 0.8 Hz, 1H), 7.65 (dd, *J* = 8.2, 7.4 Hz, 1H), 7.40 (dd, *J* = 8.3, 4.1 Hz, 1H), 3.45 (s, 3H); LC-MS (*m*/*z*) calculated for C_17_H_13_N_3_O_2_S_2_: 355.04; found: 356.1 [M + 1]^+^; purity: 100%.

#### 6-Methoxy-*N*-(propan-2-yl)-*N*-{[3-(pyridin-3-yl)-1,2,4-oxadiazol-5-yl]methyl}-2-(trifluoromethyl)quinazolin-4-amine (**21**)

ENAMINE ID: Z2142862400; ^1^H NMR (500 MHz, DMSO-*d*_6_) δ: 9.03 (s, 1H), 8.78–8.73 (m, 1H), 8.30–8.21 (m, 1H), 7.94–7.88 (m, 1H), 7.65–7.54 (m, 2H), 7.44 (s, 1H), 5.18 (s, 2H), 4.98–4.88 (m, 1H), 2.94 (s, 3H), 1.53–1.40 (m, 6H); LC-MS (*m*/*z*) calculated for C_21_H_19_F_3_N_6_O_2_: 444.15; found: 445.0 [M + 1]^+^; purity: 99%.

#### 2-Methyl-8-[(4-{thieno[3,2-c]pyridin-4-yl}piperazin-1-yl)sulfonyl]quinoline (**22**)

ENAMINE ID: Z2145689641; ^1^H NMR (500 MHz, DMSO-*d*_6_) δ: 8.43–8.33 (m, 2H), 8.28–8.21 (m, 1H), 7.99–7.93 (m, 1H), 7.74–7.65 (m, 2H), 7.59–7.43 (m, 3H), 3.61–3.49 (m, 4H), 3.48–3.38 (m, 4H), 2.71 (s, 3H); LC-MS (*m*/*z*) calculated for C_21_H_20_N_4_O_2_S_2_: 424.10; found: 425.0 [M + 1]^+^; purity: 100%.

#### 2-[(7-Chloroquinolin-4-yl)sulfanyl]-*N*-(1-{5-[(propan-2-yloxy)methyl]-1,2,4-oxadiazol-3-yl}ethyl)propenamide (**23**)

ENAMINE ID: Z2184940497; ^1^H NMR (600 MHz, DMSO-*d*_6_) δ: 9.15–8.93 (m, 1H), 8.80–8.70 (m, 1H), 8.15–8.03 (m, 2H), 7.70–7.63 (m, 1H), 7.53–7.43 (m, 1H), 5.10–5.00 (m, 1H), 4.70 (s, 2H), 4.40–4.30 (m, 1H), 3.75–3.65 (m, 1H), 1.60–1.35 (m, 6H), 1.18–1.08 (m, 6H); LC-MS (*m*/*z*) calculated for C_20_H_23_ClN_4_O_3_S: 434.12; found: 435.0 [M + 1]^+^; purity: 100%.

#### *N*^4^-(2,4-Dimethoxyphenyl)-*N*^6^-{2-[5-(propan-2-yl)-1,3,4-thiadiazol-2-yl]ethyl}pyrimidine-4,6-diamine (**24**)

ENAMINE ID: Z2199974094; ^1^H NMR (500 MHz, DMSO-*d*_6_) δ: 7.98 (s, 1H), 7.80 (s, 1H), 7.39–7.31 (m, 1H), 6.91–6.83 (m, 1H), 6.60 (s, 1H), 6.51–6.45 (m, 1H), 5.46 (s, 1H), 3.80–3.69 (m, 6H), 3.56–3.45 (m, 2H), 3.43–3.33 (m, 1H), 3.25–3.20 (m, 2H), 1.35–1.24 (m, 6H); LC-MS (*m*/*z*) calculated for C_19_H_24_N_6_O_2_S: 400.17; found: 401.0 [M + 1]^+^; purity: 98%.

#### *N*^4^-Ethyl-*N*^4^-phenyl-*N*^6^-{2-[5-(propan-2-yl)-1,3,4-thiadiazol-2-yl]ethyl}pyrimidine-4,6-diamine (**25**)

ENAMINE ID: Z2214001359; ^1^H NMR (600 MHz, DMSO-*d*_6_) δ: 8.08 (s, 1H), 7.49–7.43 (m, 2H), 7.33–7.28 (m, 1H), 7.26–7.21 (m, 2H), 6.88–6.80 (m, 1H), 5.18 (s, 1H), 3.90–3.83 (m, 2H), 3.55–3.43 (m, 2H), 3.40–3.30 (m, 1H), 3.20–3.15 (m, 2H), 1.35–1.25 (m, 6H), 1.10–1.03 (m, 3H); LC-MS (*m*/*z*) calculated for C_19_H_24_N_6_S: 368.18; found: 369.0 [M + 1]^+^; purity: 100%.

#### 6-Methoxy-4-(4-{[3-(propan-2-yl)-1,2,4-oxadiazol-5-yl]methyl}piperidin-1-yl)quinoline-3-carbonitrile (**26**)

ENAMINE ID: Z2434240495; ^1^H NMR (600 MHz, DMSO-*d*_6_) δ: 8.60 (s, 1H), 7.91–7.89 (m, 1H), 7.51–7.48 (m, 1H), 7.26–7.20 (m, 1H), 3.90 (s, 3H), 3.81–3.75 (m, 2H), 3.46–3.38 (m, 2H), 3.09–3.00 (m, 1H), 3.00–2.98 (m, 2H), 2.20–2.11 (m, 1H), 1.94–1.83 (m, 2H), 1.68–1.58 (m, 2H), 1.30–1.19 (m, 6H); LC-MS (*m*/*z*) calculated for C_22_H_25_N_5_O_2_: 391.20; found: 392.3 [M + 1]^+^; purity: 100%.

#### 6-[3-(1-Methyl-1*H*-indol-3-yl)-1,2,4-oxadiazol-5-yl]-*N*-phenylpyrimidin-4-amine (**27**)

ENAMINE ID: Z2902917812; ^1^H NMR (600 MHz, DMSO-*d*_6_) δ: 10.13 (s, 1H), 8.83 (s, 1H), 8.20 (s, 1H), 8.15–8.08 (m, 1H), 7.80–7.58 (m, 4H), 7.43–7.25 (m, 4H), 7.15–7.05 (m, 1H), 3.91 (s, 3H); LC-MS (*m*/*z*) calculated for C_21_H_16_N_6_O: 368.14; found: 369.0 [M + 1]^+^; purity: 100%.

#### 8-Methoxy-*N*-{2-[5-(propan-2-yl)-1,3,4-thiadiazol-2-yl]ethyl}quinazolin-4-amine (**28**)

ENAMINE ID: Z3019339476; ^1^H NMR (600 MHz, DMSO-*d*_6_) δ: 8.43 (s, 1H), 8.40–8.30 (m, 1H), 7.73–7.65 (m, 1H), 7.48–7.40 (m, 1H), 7.28–7.20 (m, 1H), 3.98–3.85 (m, 4H), 3.50–3.40 (m, 2H), 3.40–3.34 (m, 2H), 1.38–1.25 (m, 6H); LC-MS (*m*/*z*) calculated for C_16_H_19_N_5_OS: 329.13; found: 330.0 [M + 1]^+^; purity: 96%.

#### *N*-(7,8-Difluoroquinolin-3-yl)-4-(propan-2-yl)-1,2,3-thiadiazole-5-carboxamide (**29**)

ENAMINE ID: Z4595013321; ^1^H NMR (500 MHz, DMSO-*d*_6_) δ: 11.35 (s, 1H), 9.10–9.01 (m, 1H), 8.98–8.85 (m, 1H), 7.99–7.89 (m, 1H), 7.80–7.69 (m, 1H), 3.74–3.61 (m, 1H), 1.49–1.35 (m, 6H); LC-MS (*m*/*z*) calculated for C_15_H_12_FN_5_OS: 334.07; found: 335.0 [M + 1]^+^; purity: 100%.

#### 7,8-Difluoro-*N*-[1-(propan-2-yl)-1*H*-indazol-5-yl]quinazolin-4-amine (**30**)

ENAMINE ID: Z4595013374; ^1^H NMR (500 MHz, DMSO-*d*_6_) δ: 10.10 (s, 1H), 8.55 (s, 1H), 8.48–8.40 (m, 1H), 8.10 (s, 1H), 8.06 (s, 1H), 7.79–7.66 (m, 2H), 7.66–7.60 (m, 1H), 5.05–4.95 (m, 1H), 1.55–1.40 (m, 6H); LC-MS (*m*/*z*) calculated for C_18_H_15_F_2_N_5_: 339.13; found: 340.2 [M + 1]^+^; purity: 100%.

#### 3-(3-Chloro-2-methoxyphenyl)-5-{thieno[2,3-*b*]pyridin-5-yl}-1,2,4-oxadiazole (**31**)

ENAMINE ID: Z4595013397; ^1^H NMR (600 MHz, DMSO-*d*_6_) δ: 9.30 (s, 1H), 9.09 (s, 1H), 8.11–8.08 (m, 1H), 8.03–7.99 (m, 1H), 7.80–7.75 (m, 1H), 7.70–7.65 (m, 1H), 7.43–7.36 (m, 1H), 3.90 (s, 3H); LC-MS (*m*/*z*) calculated for C_16_H_10_ClN_3_O_2_S: 343.02; found: 344.0 [M + 1]^+^; purity: 96%.

#### 8-Fluoro-3-(5-{[1-(propan-2-yl)-1*H*-pyrazol-3-yl]methyl}-1,2,4-oxadiazol-3-yl)quinoline (**32**)

ENAMINE ID: Z4595013410; ^1^H NMR (600 MHz, DMSO-*d*_6_) δ: 9.48 (s, 1H), 9.10 (s, 1H), 8.10–8.03 (m, 1H), 7.80–7.63 (m, 3H), 6.30–6.23 (m, 1H), 4.50–4.40 (m, 3H), 1.45–1.35 (m, 6H); LC-MS (*m*/*z*) calculated for C_18_H_16_FN_5_O: 337.13; found: 338.2 [M + 1]^+^; purity: 100%.

#### 3-Chloro-*N*-[3-(methylsulfanyl)-1,2,4-thiadiazol-5-yl]thieno[2,3-*b*]pyridine-2-carboxamide (**33**)

ENAMINE ID: Z4595013450; ^1^H NMR (500 MHz, DMSO-*d*_6_) δ: 13.81 (s, 1H), 8.82 (dd, *J* = 4.6, 1.6 Hz, 1H), 8.38 (d, *J* = 8.2 Hz, 1H), 7.67 (dd, *J* = 8.2, 4.6 Hz, 1H), 2.60 (s, 3H); LC-MS (*m*/*z*) calculated for C_11_H_7_ClN_4_OS_3_: 341.95; found: 343.0 [M + 1]^+^; purity: 100%.

### Biological Investigation

#### Materials

Cyclosporine A and compound **34** were obtained from Tocris Bioscience (Bristol, UK). Calcein AM and pheophorbide A were purchased from Calbiochem [EMD Chemicals (San Diego, USA), supplied by Merck KgaA (Darmstadt, Germany)]. Other chemicals were delivered by Carl Roth (Karlsruhe, Germany), Merck KgaA (Darmstadt, Germany), or Sigma-Aldrich (Taufkirchen, Germany). Ten millimolar DMSO stock solutions of cyclosporine A, compound **34**, and compounds **11**–**33** were prepared and stored at −20 °C. Dilution series of the respective compounds and in-experiment cell culture was performed with Krebs-HEPES buffer [KHB; 118.6 mM NaCl, 4.7 mM KCl, 1.2 mM KH_2_PO_4_, 4.2 mM NaHCO_3_, 1.3 mM CaCl_2_, 1.2 mM MgSO_4_, 11.7 mM d-glucose monohydrate, 10.0 mM HEPES (2-[4-(2-hydroxyethyl)piperazin-1-yl]ethanesulfonic acid) in doubly distilled water; adjusted to pH 7.4 with NaOH; sterilized with 0.2 μm membrane filters].

#### Cell Culture

A2780/ADR cells were delivered by European Collection of Animal Cell Culture (ECACC, no. 93112520) and cultured with RPMI-1640 medium (PAN-Biotech GmbH, Aidenbach, Germany) supplemented with 10% fetal bovine serum (FCS; PAN-Biotech GmbH, Aidenbach, Germany), 50 μg/μL streptomycin (PAN-Biotech GmbH, Aidenbach, Germany), 50 U/mL penicillin G (PAN-Biotech GmbH, Aidenbach, Germany), and 2 mM l-glutamine (PAN-Biotech GmbH, Aidenbach, Germany). H69AR cells were provided by American Type Culture Collection (ATCC CRL-11351) and cultivation was performed using RPMI-1640 medium supplemented with 20% FCS, 50 μg/μL streptomycin, 50 U/mL penicillin G, and 2 mM l-glutamine. MDCK II BCRP cells were a generous gift from Dr. A. Schinkel (The Netherlands Cancer Institute, Amsterdam, The Netherlands)^75^ and cultured in Dulbecco’s modified eagle medium (DMEM; Sigma Life Science, Steinheim, Germany) supplemented with 10% FCS, 50 μg/μL streptomycin, 50 U/mL penicillin G, and 2 mM l-glutamine. Cells were stored under liquid nitrogen in medium (90%) and DMSO (10%) before culturing (5% CO_2_-humidified atmosphere; 37 °C). Cell harvesting was performed at a confluence of at least 90% by exposure to a trypsin (0.05%)-EDTA (0.02%) solution (PAN-Biotech GmbH, Aidenbach, Germany). Cells were subsequently collected, centrifuged in a 50 mL falcon (Greiner Bio-One, Frickenhausen, Germany) at 266*g*, 4 °C, 4 min (Avanti J-25, Beckmann Coulter, Krefeld, Germany), supernatant removal and resuspension in fresh media, counted (CASY TT cell counter with 150 μm capillary, Schärfe System GmbH, Reutlingen, Germany), and seeded in right amount for sub-culturing or biological testing.

#### Calcein AM Assay

Calcein AM assays to assess inhibitory activity against ABCB1 and ABCC1 were applied as described earlier.^14−16,56^ Twenty micromolar of either 50 or 100 μM of compounds **11**–**33** were added to a 96-well flat-bottom clear plate (Greiner, Frickenhausen, Germany) and complemented with 160 μL of cell suspension containing either A2780/ADR or H69AR cells at a density of 30,000 and 60,000 cells/well, respectively. After incubation (5% CO_2_-humidified atmosphere; 37 °C) for 30 min, calcein AM (3.125 μM; 20 μL) was added to each well followed by immediate measurement of fluorescence increase (excitation: 485 nm; emission: 520 nm; interval: 60 s; duration: 1 h) using POLARstar and FLUOstar Optima microplate readers (BMG Labtech, software versions 2.00R2/2.20 and 4.11-0; Offenburg, Germany). Slopes from the linear fluorescence increase were calculated and compared to the respective slopes of the standard inhibitors. To determine IC_50_ values, in-depth concentration-effect curves have been generated by plotting the slopes against several logarithmic concentrations of the tested compounds. Data analysis was performed using GraphPad Prism (version 8.4.0, San Diego, CA, USA) using the statistically preferred model (three- or four-parameter logistic equation).

#### Pheophorbide A Assay

The pheophorbide A assay to assess inhibitory activity against ABCG2 was applied as described earlier.^14−16^ Compound and cell preparation was conducted as described above. In total, 45,000 cells in a 160 μL suspension were pipetted into flat-bottom clear 96-well plates after 20 μL of the respective compound dilution has been added (Thermo Scientific, Rochester, NY, USA). A pheophorbide A solution (20 μL; 5 μM) was supplemented, and the reaction mixture was incubated for 120 min (5% CO_2_-humidified atmosphere; 37 °C). Eventually, the intracellular fluorescence was detected *via* flow cytometry (Guava easyCyte HT, Merck Millipore, Billerica, MA, USA) at an excitation wavelength of 488 nm and emission wavelength of 695/50 nm. The absolute fluorescence values were compared to the effect caused by the standard ABCG2 inhibitor compound **34**. Determination of relative inhibition and IC_50_ values were determined as described above.

#### Hoechst 33342 Assay

To confirm the inhibitory effect of compounds **15**, **18**, **21**, **22**, and **26** against ABCB1 and ABCG2, as well as compound **27** against ABCG2, a Hoechst 33342 assay was performed as described earlier.^15,57^ Twenty microliters of the dilutions of the compounds in KHB were pipetted into black 96-well plates (Greiner, Frickenhausen, Germany). Cells were processed as described before, and approximately 30,000 were seeded into the plates with 160 μL per well. After a 30 min incubation period at 37 °C and 5% CO_2_, Hoechst 33342 solution (10 μM) was added at a quantity of 20 μL resulting in a final Hoechst 33342 concentration of 1 μM. Fluorescence intensity was assessed in 60 s time intervals for a time period of 120 min at an excitation wavelength of 355 nm and an emission wavelength of 460 nm using microplate readers (POLARstar and FLUOstar Optima by BMG Labtech, Offenburg, Germany). The average fluorescence values at the steady state were calculated for each concentration and plotted against the logarithm of the compound concentration. Determination of relative inhibition and IC_50_ values were determined as described above.

#### Daunorubicin Accumulation Assay

For further confirmation of the inhibitory potency of triple inhibitors on ABCC1, daunorubicin accumulation assay was applied as described before with minor modifications. Dilution series of test compounds and cell culture were performed as described for the calcein AM assay. To 20 μL of the test compounds in different concentrations in a clear flat-bottom 96-well plate (Thermo Scientific, Waltham, MA, USA), 160 μL of the cell suspension containing approximately 45,000 H69 AR cells in colorless culture medium without supplements was added. Then, 20 μL of a 30 μM daunorubicin solution were pipetted to the mixture and incubated for 180 min protected from light at 37 °C and a 5% CO_2_ humidified atmosphere. Fluorescence was measured by flow cytometry (Guava easyCyte HT) at a 488 nm excitation wavelength and 695/50 nm emission wavelength. Data analysis was performed as described before. Determination of relative inhibition and IC_50_ values were determined as described above.

## Supporting Information Available

The complete dataset of categorized and classified 1049 compounds (Supplementary Tables 1–3); statistical analysis of substructure search (Supplementary Table 4); and molecular formula strings of “Screen 5” compounds before manual selection (Supplementary Table 5) (ZIP)
Molecular formula strings and the biological data of compounds **11**–**33** (Supplementary Table 6) (CSV)
Concentration-effect curves of compound **15** against ABCB1 (A and D), ABCC1 (B and E), and ABCG2 (C and F) as determined in calcein AM (A and B), pheophorbide A (C), Hoechst 33342 (D and F), and daunorubicin (E) assays (Supplementary Figure 1 A–F); concentration-effect curves of compound **18** against ABCB1 (A and D), ABCC1 (B and E), and ABCG2 (C and F) as determined in calcein AM (A and B), pheophorbide A (C), Hoechst 33342 (D and F), and daunorubicin (E) assays (Supplementary Figure 2 A–F); concentration-effect curves of compound **22** against ABCB1 (A and D), ABCC1 (B and E), and ABCG2 (C and F) as determined in calcein AM (A and B), pheophorbide A (C), Hoechst 33342 (D and F), and daunorubicin (E) assays (Supplementary Figure 3 A–F); concentration-effect curves of compound **26** against ABCB1 (A and D), ABCC1 (B and E), and ABCG2 (C and F) as determined in calcein AM (A and B), pheophorbide A (C), Hoechst 33342 (D and F), and daunorubicin (E) assays (Supplementary Figure 4 A–F); and LC-MS data of compounds **11**–**33** as provided by ENAMINE (PDF)

## Abbreviations Used

ABC: ATP-binding cassette
BCRP: breast cancer resistance protein
calcein AM: calcein acetoxymethyl
C@PA: computer-aided pattern analysis
EC_50_: half-maximal reversal concentration
IC_50_: half-maximal inhibition concentration
I_max_: maximal inhibition level
I_rel_: relative inhibition
LogP: partition coefficient
MDR: multidrug resistance
MRP1: multidrug resistance-associated protein 1
MW: molecular weight
P-gp: P-glycoprotein
SMILES: simplified molecular input line entry specification
Tc: Tanimoto coefficient.
