# Supplementary material for: C@PA: Computer-Aided Pattern Analysis to Predict Multitarget ABC Transporter Inhibitors
Source: J Med Chem. 2021 Mar 16;64(6):3350–66. doi: 10.1021/acs.jmedchem.0c02199 (PMC8041314; doi:10.1021/acs.jmedchem.0c02199)
Supplement: Supplementary file 3 — jm0c02199_si_003.pdf [file jm0c02199_si_003.pdf]

Supporting Information.

C@PA: Computer-aided Pattern Analysis to Predict Multitarget ABC Transporter Inhibitors

Vigneshwaran Namasivayam[a], Katja Silbermann[a], Michael Wiese[a], Jens Pahnke[b,c,d,e], Sven Marcel Stefan[,a,b,f]*

[a] Department of Pharmaceutical and Cellbiological Chemistry, University of Bonn, An der Immenburg 4, 53121 Bonn, Germany

[b] Department of Neuro-/Pathology, University of Oslo and Oslo University Hospital, Sognsvannsveien 20, 0372 Oslo, Norway

[c] LIED, University of Lübeck, Ratzenburger Allee 160, 23538 Lübeck, Germany

[d] Department of Pharmacology, Faculty of Medicine, University of Latvia, Jelgava Iela, 1004 Riga, Latvia

[e] Department of Bioorganic Chemistry, Leibniz-Institute of Plant Biochemistry, Weinberg 3, 06120 Halle, Germany

[f] Cancer Drug Resistance and Stem Cell Program, University of Sydney, Kolling Builging, 10 Westbourne Street, Sydney, New South Wales 2065, Australia.

* Corresponding author: Sven Marcel Stefan (s.m.stefan@medisin.uio.no)

Table of contents.

Supplementary Figure 1 A–F: Concentration-effect curves of compound **15** against ABCB1 (A and D), ABCC1 (B and E), and ABCG2 (C and F) as determined in calcein AM (A and B), pheophorbide A (C), Hoechst 33342 (D and F), and daunorubicin (E) assays.

Supplementary Figure 2 A–F: Concentration-effect curves of compound **18** against ABCB1 (A and D), ABCC1 (B and E), and ABCG2 (C and F) as determined in calcein AM (A and B), pheophorbide A (C), Hoechst 33342 (D and F), and daunorubicin (E) assays.

Supplementary Figure 3 A–F: Concentration-effect curves of compound **22** against ABCB1 (A and D), ABCC1 (B and E), and ABCG2 (C and F) as determined in calcein AM (A and B), pheophorbide A (C), Hoechst 33342 (D and F), and daunorubicin (E) assays.

Supplementary Figure 4 A–F: Concentration-effect curves of compound **26** against ABCB1 (A and D), ABCC1 (B and E), and ABCG2 (C and F) as determined in calcein AM (A and B), pheophorbide A (C), Hoechst 33342 (D and F), and daunorubicin (E) assays.

*LC-MS data and NMR spectra of compounds **11–33**.*

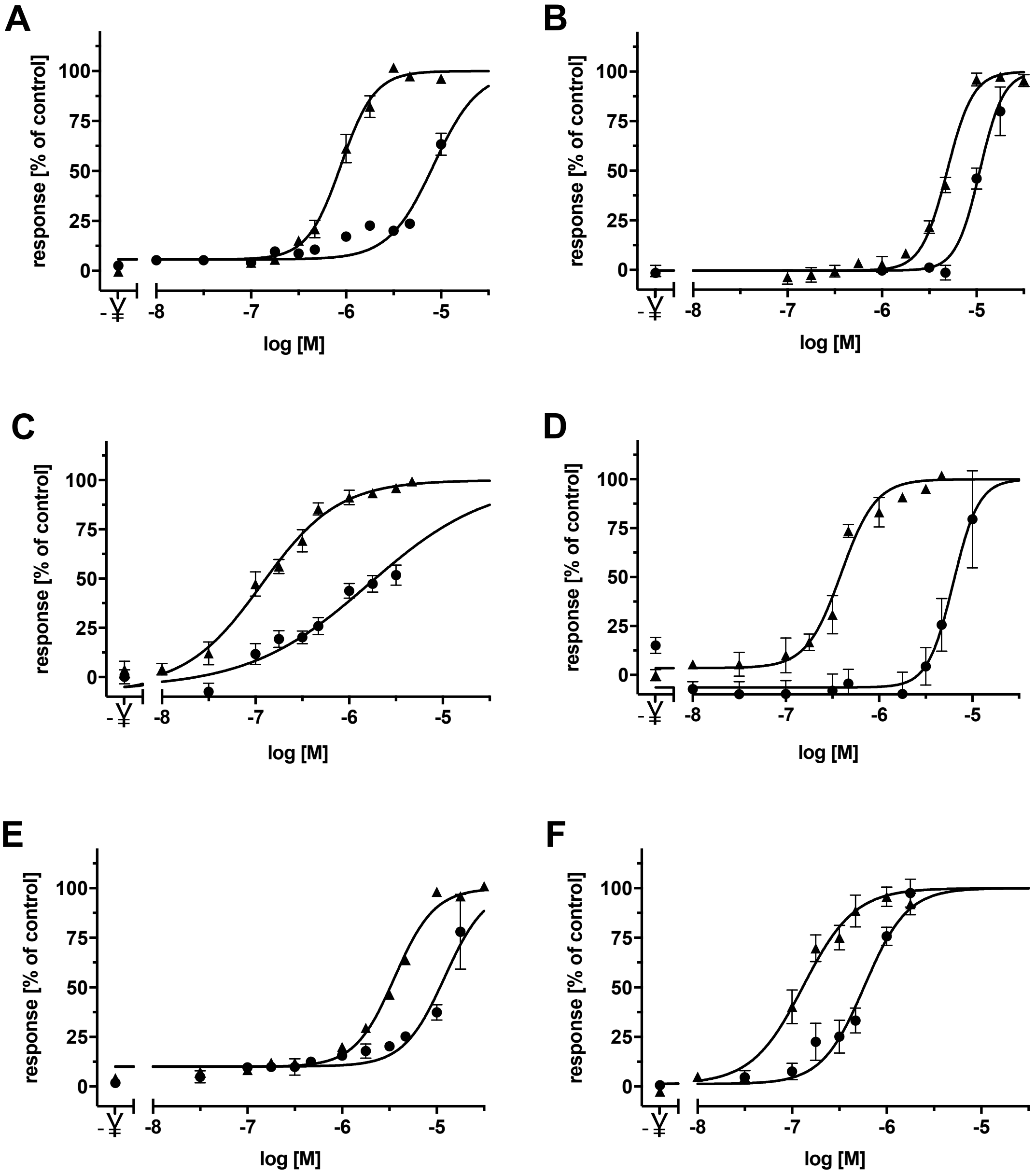

Supplementary Figure 1 A–F: Concentration-effect curves of compound **15** (●) as determined in calcein AM (A and B), pheophorbide A (C), Hoechst 33342 (D and F), and daunorubicin (E) assays using either ABCB1-overexpressing A2780/ADR cells (A and D), ABCC1-overexpressing H69AR cells (B and E), or ABCG2-overexpressing MDCK II BCRP cells (C and F). Data were normalized by defining the effect of 10 µM cyclosporine A (▲; A, B, D, and E) and compound **34** (▲; C and F) as positive control (100%) and buffer medium was used as negative control (0%). Shown is mean ± SEM of at least three independent experiments.

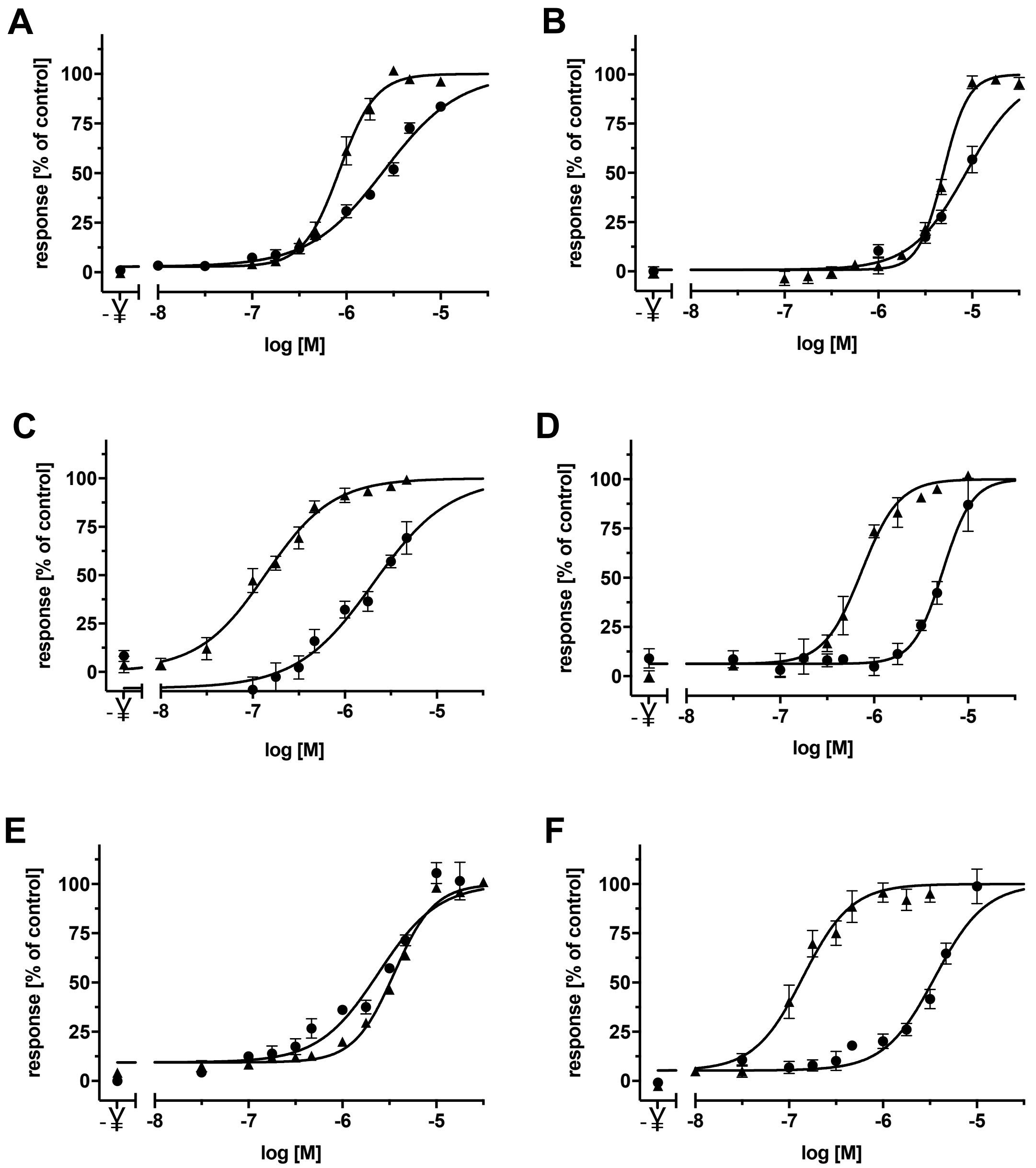

Supplementary Figure 2 A–F: Concentration-effect curves of compound **18** (●) as determined in calcein AM (A and B), pheophorbide A (C), Hoechst 33342 (D and F), and daunorubicin (E) assays using either ABCB1-overexpressing A2780/ADR cells (A and D), ABCC1-overexpressing H69AR cells (B and E), or ABCG2-overexpressing MDCK II BCRP cells (C and F). Data were normalized by defining the effect of 10 µM cyclosporine A (▲; A, B, D, and E) and compound **34** (▲; C and F) as positive control (100%) and buffer medium was used as negative control (0%). Shown is mean ± SEM of at least three independent experiments.

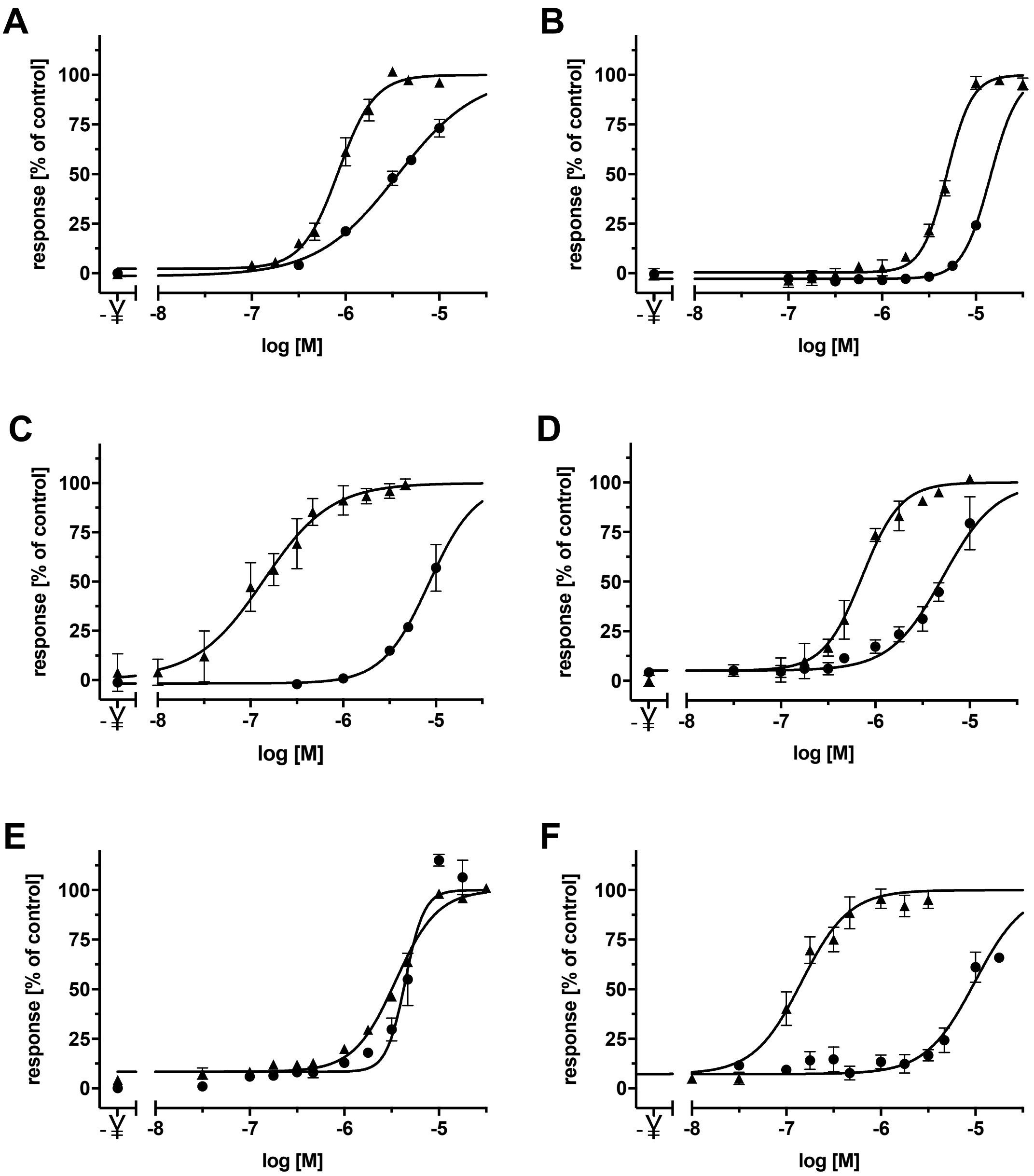

Supplementary Figure 3 A–F: Concentration-effect curves of compound **22** (●) as determined in calcein AM (A and B), pheophorbide A (C), Hoechst 33342 (D and F), and daunorubicin (E) assays using either ABCB1-overexpressing A2780/ADR cells (A and D), ABCC1-overexpressing H69AR cells (B and E), or ABCG2-overexpressing MDCK II BCRP cells (C and F). Data were normalized by defining the effect of 10 μM cyclosporine A (▲; A, B, D, and E) and compound **34** (▲; C and F) as positive control (100%) and buffer medium was used as negative control (0%). Shown is mean ± SEM of at least three independent experiments.

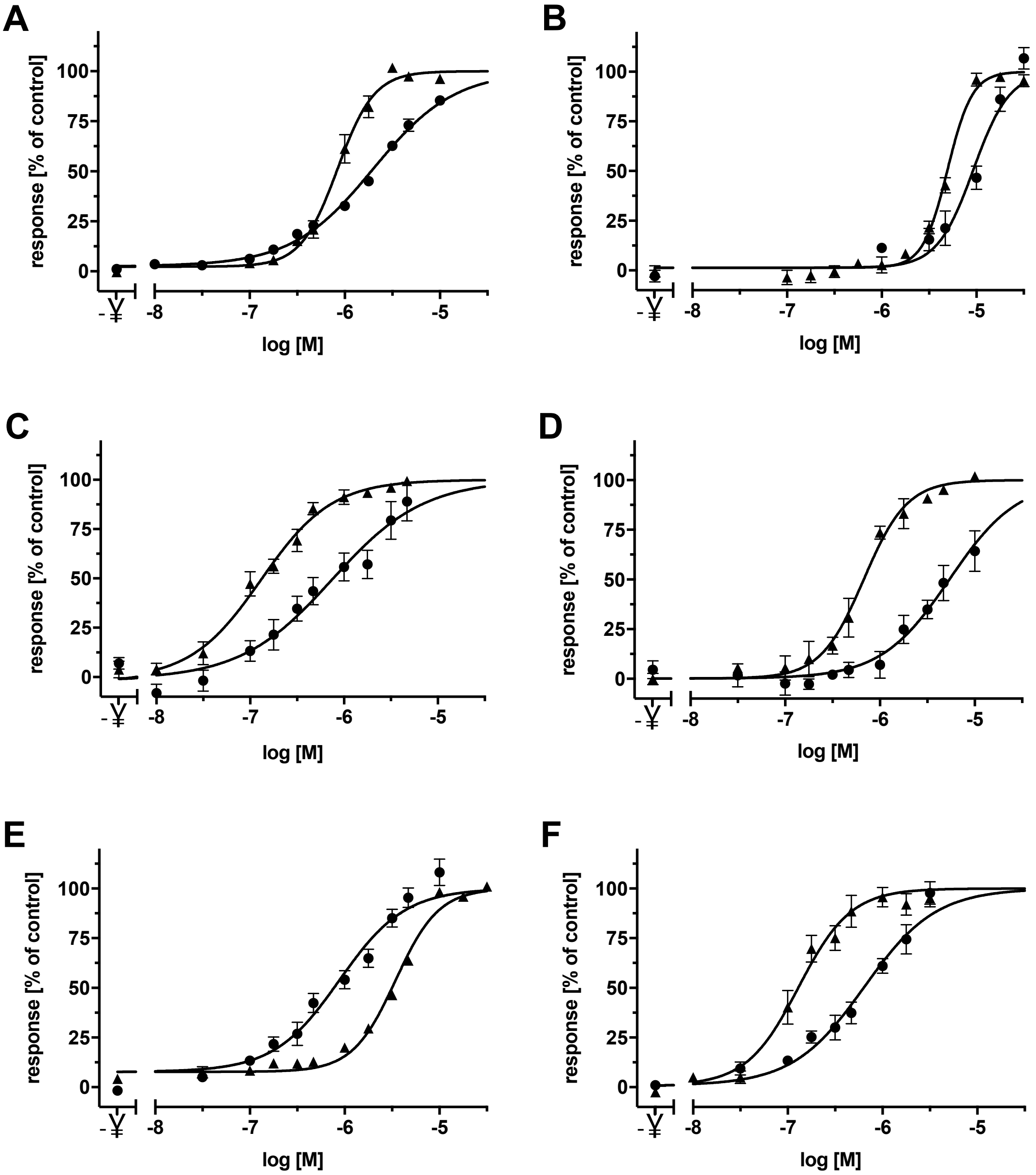

Supplementary Figure 4 A–F: Concentration-effect curves of compound **26** (●) as determined in calcein AM (A and B), pheophorbide A (C), Hoechst 33342 (D and F), and daunorubicin (E) assays using either ABCB1-overexpressing A2780/ADR cells (A and D), ABCC1-overexpressing H69AR cells (B and E), or ABCG2-overexpressing MDCK II BCRP cells (C and F). Data were normalized by defining the effect of 10 µM cyclosporine A (▲; A, B, D, and E) and compound **34** (▲; C and F) as positive control (100%) and buffer medium was used as negative control (0%). Shown is mean ± SEM of at least three independent experiments.

Compound **11**

Sample H2658185

0.1%FA-ACN 5-95%ACN 6 min. Zorbax-Eclipse-Plus C18 4,6 x 100mm, 3.5 mkm.

#	Time	Area%
1	4.575	100.00

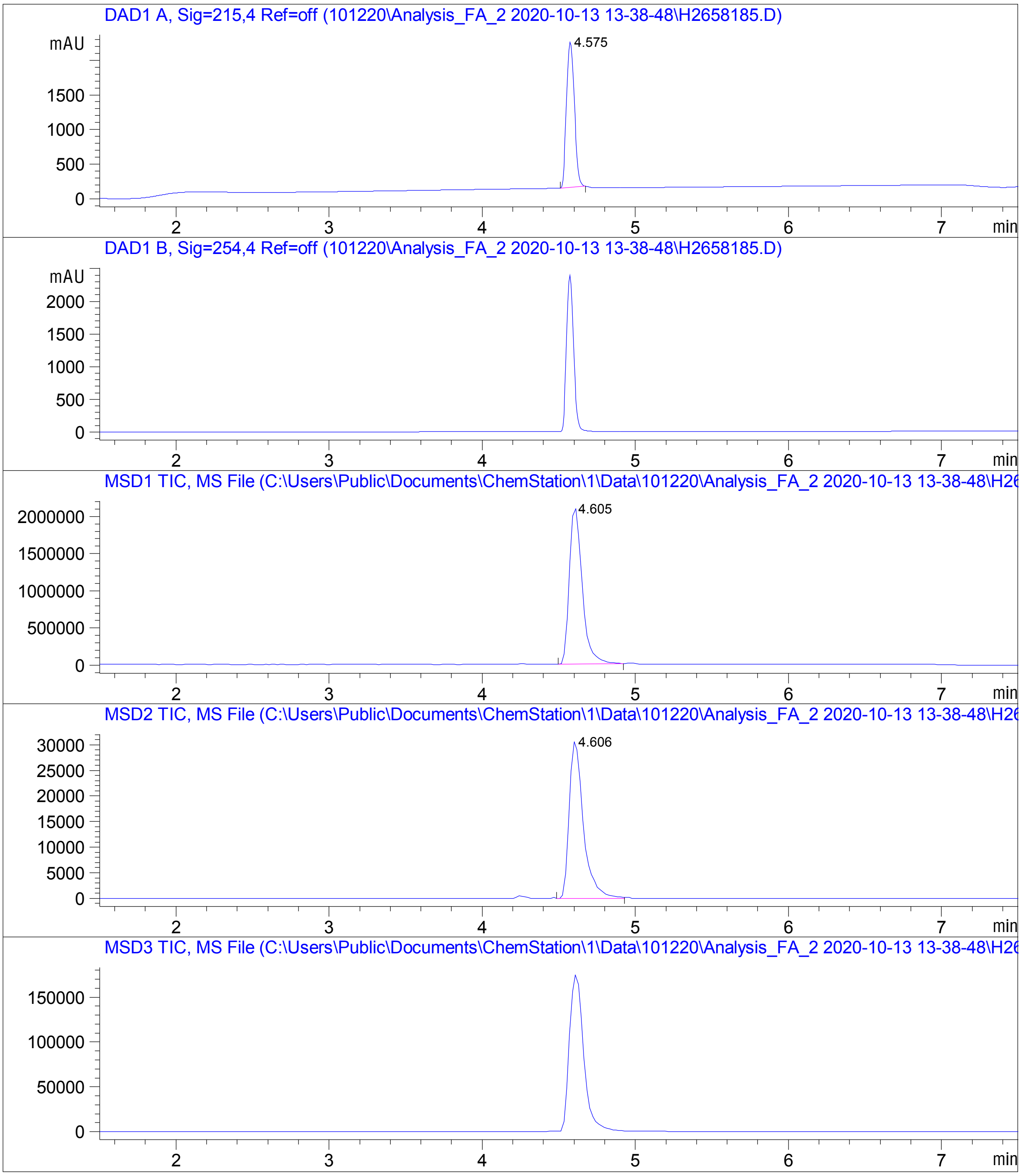

RT 4.605

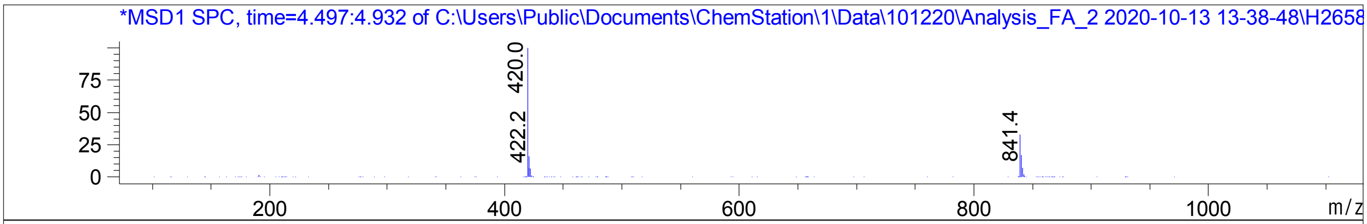

RT 4.606

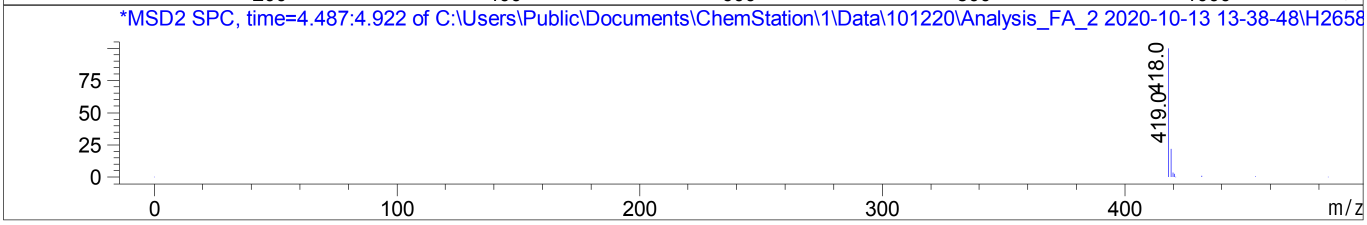

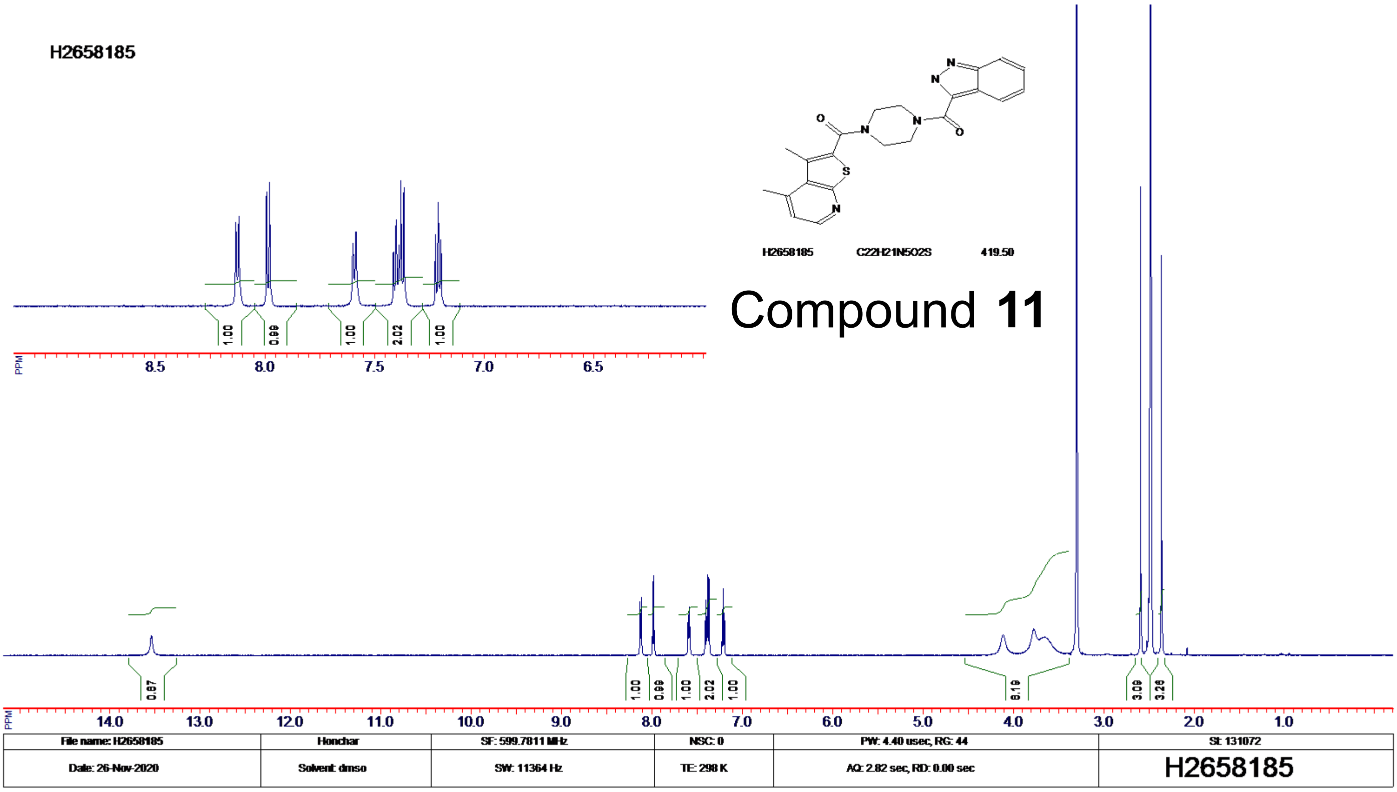
H2658185
H2658185 C22H21N5O2S 419.50
Compound 11
File name: H2658185
Honchar
SF: 599.7811 MHz
NSC: 0
PW: 4.40 usec, RG: 44
St: 131072
Date: 26-Nov-2020
Solvent: dmso
SW: 11364 Hz
TE: 298 K
AQ: 2.82 sec, RD: 0.00 sec
H2658185

Compound **12**

Sample H2660984 0.1%FA-ACN 5-95%ACN 6 min. Zorbax-Eclipse-Plus C18 4,6 x 100mm, 3.5 mkm.

#	Time	Area%
1	5.113	100.00

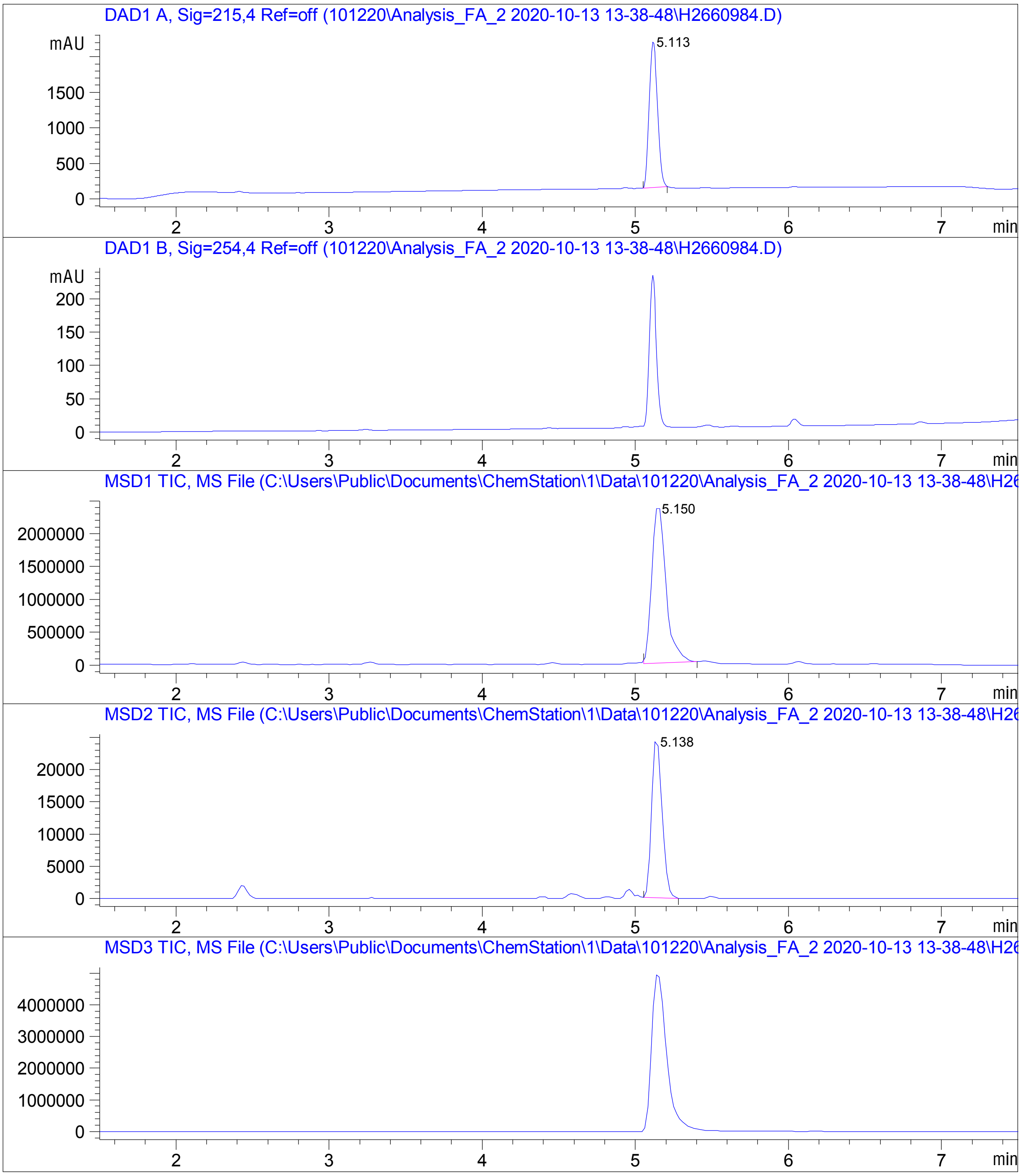

RT 5.150

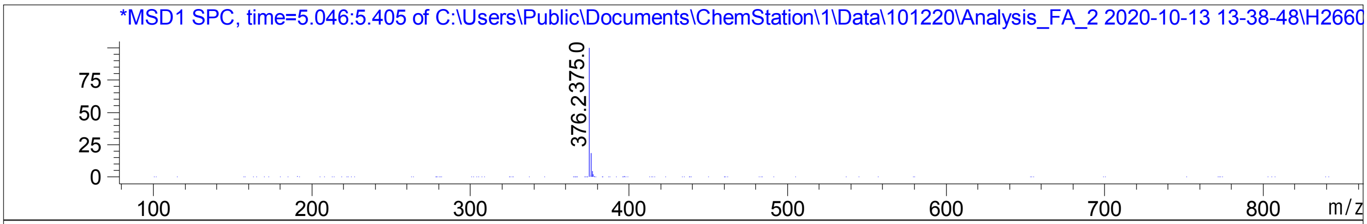

RT 5.138

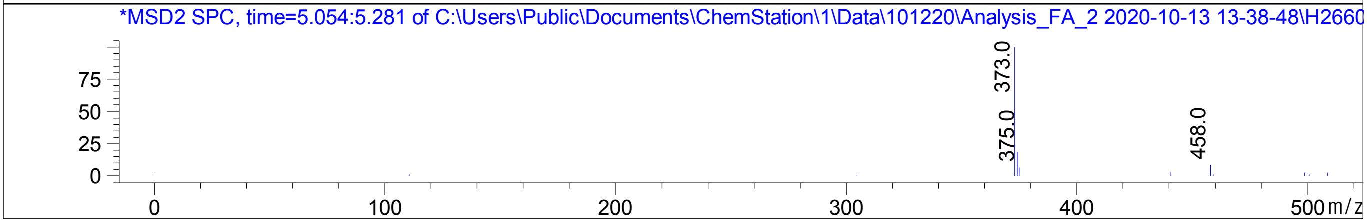

Tue, 13.->13.10.2020 S-> E D1F-A8 Acq. Method C:\Users\ ->

H2660984

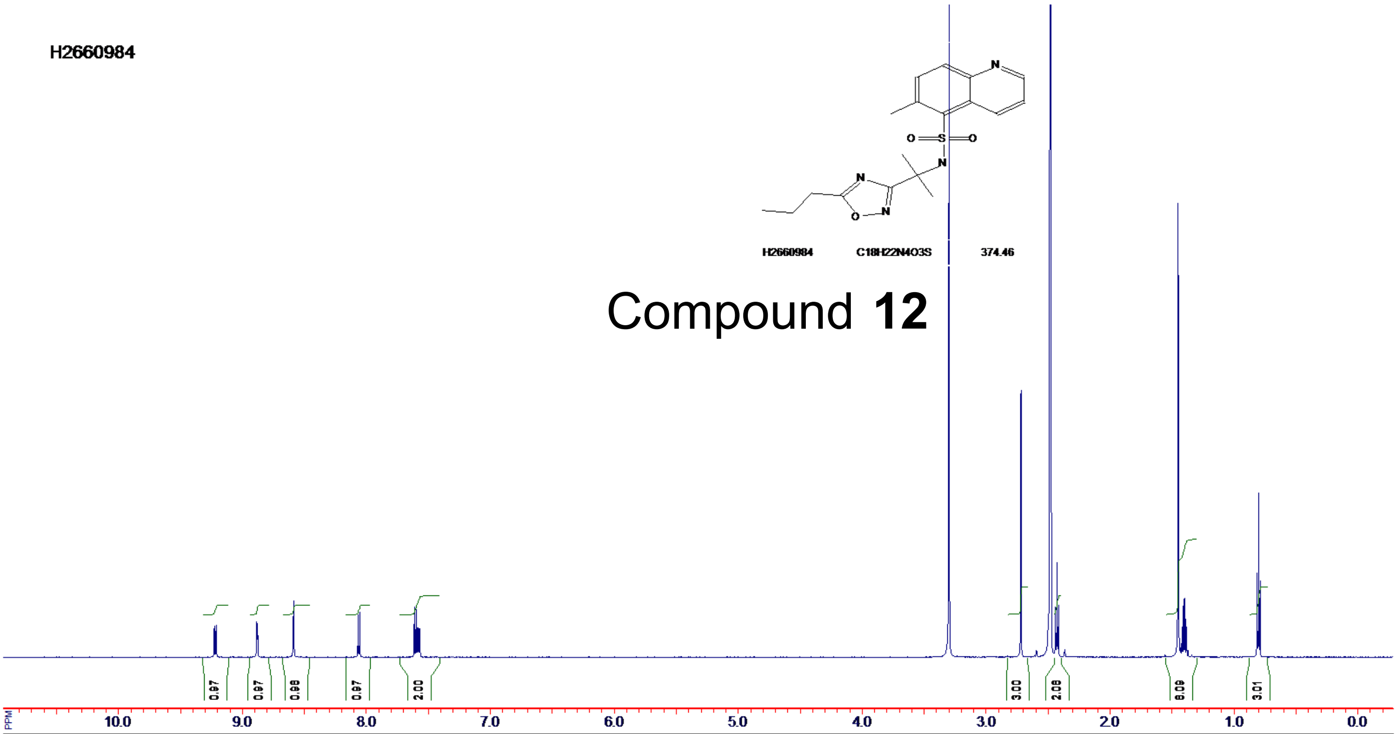

Compound **12**

File name: H2660984	Honchar	SF: 599.7811 MHz	NSC: 0	PW: 4.40 usec, RG: 44	St: 131072
Date: 26-Nov-2020	Solvent: dmso	SW: 11364 Hz	TE: 298 K	AQ: 2.82 sec, RD: 0.00 sec	H2660984

Compound **13**

MaxPeak: 100.00%
Ret_Time: 0.981 min

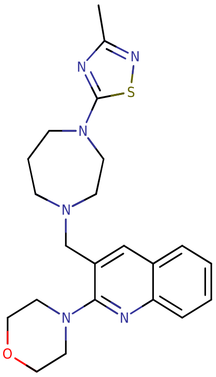

Mol Wt 424.56

Exact Mass 424.24

#	Time	Area%
1	0.981	100.00

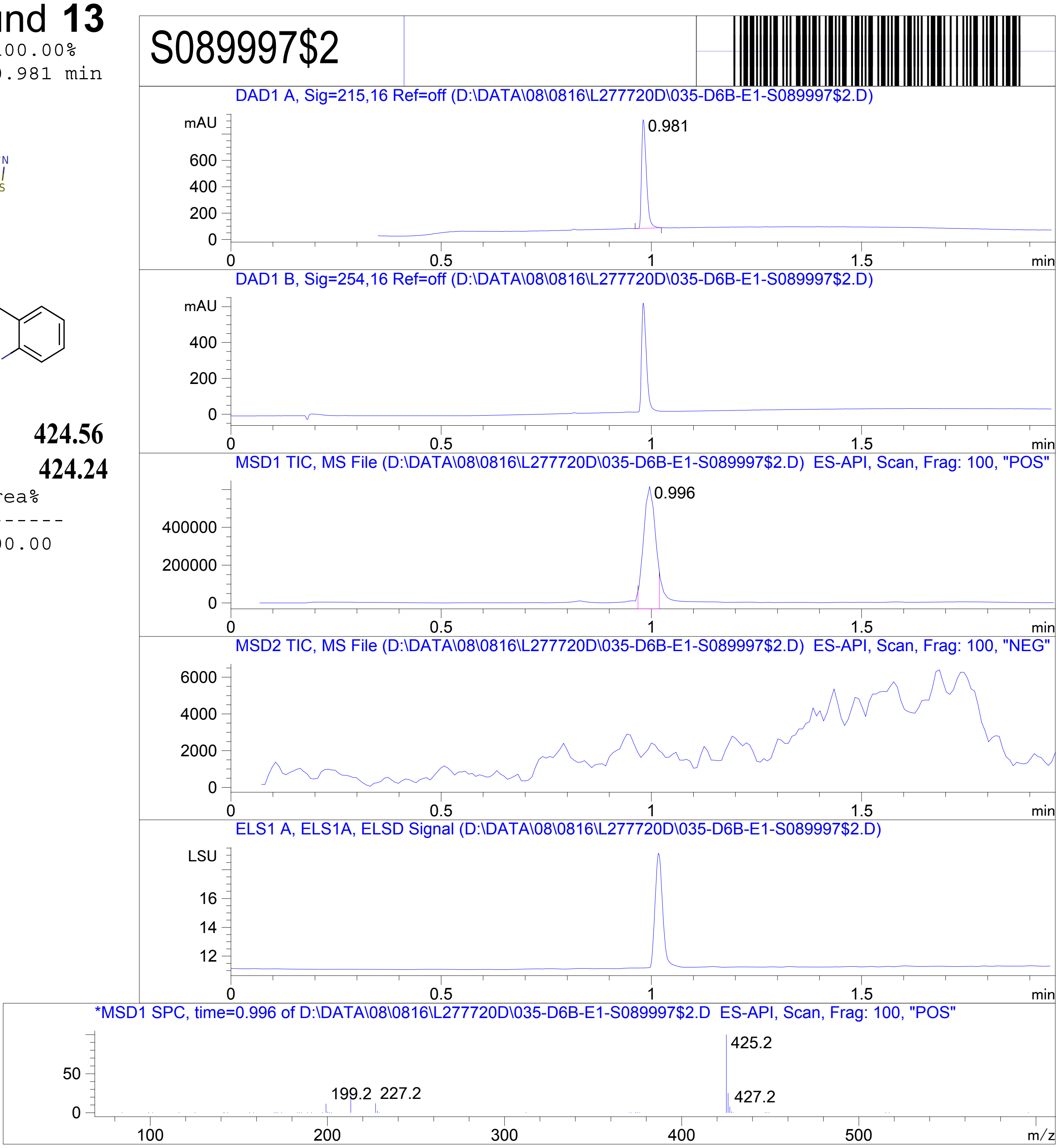

RT 0.996

H2562999

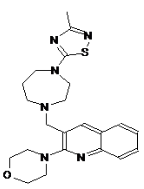

H2562999 C22H28N6OS 424.56

Compound **13**

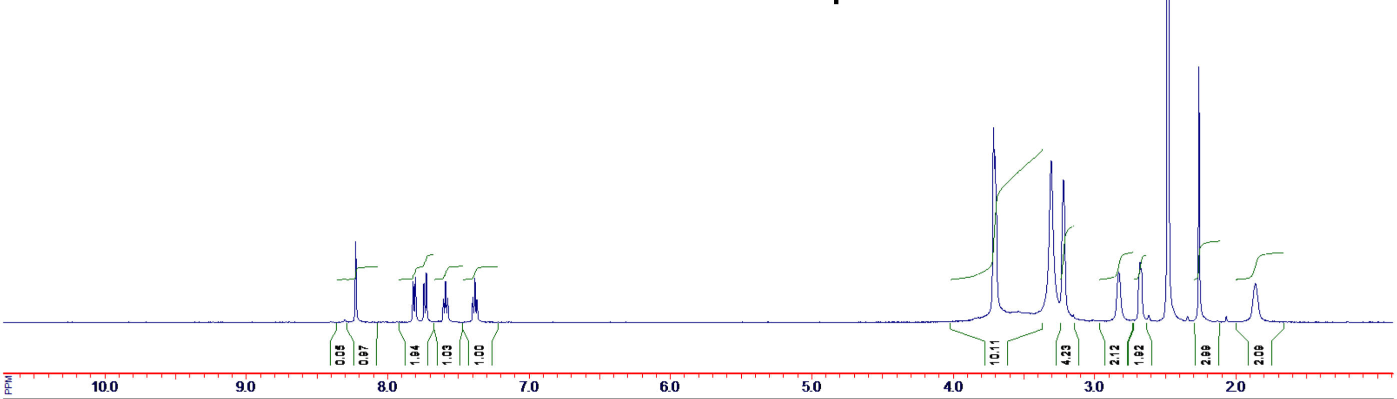

File name: H2562999	Honchar	SF: 499.8203 MHz	NSC: 0	PW: 12.40 usec, RG: 14	SI: 65536
Date: 01-Dec-2020	Solvent: dmso	SW: 9542 Hz	TE: 298 K	AQ: 1.68 sec, RD: 0.00 sec	H2562999

Compound **14**

MaxPeak: 97.99%
Ret_Time: 0.999 min

S089976$2

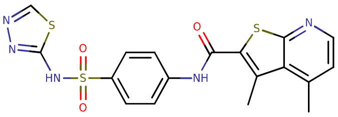

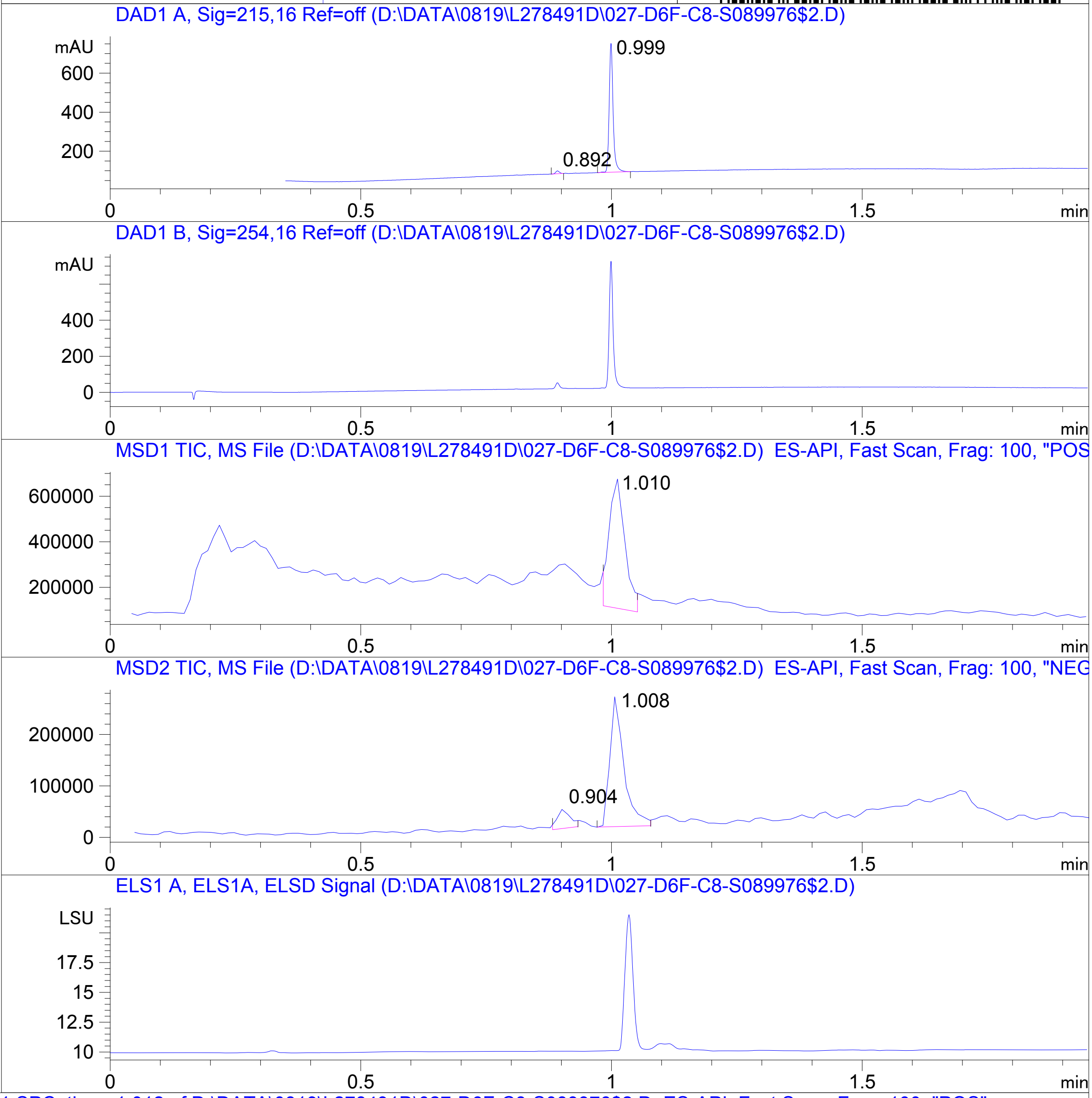

Mol Wt 445.54

Exact Mass 445.03

#	Time	Area%
1	0.892	2.01
2	0.999	97.99

RT 1.010

RT 0.904

RT 1.008

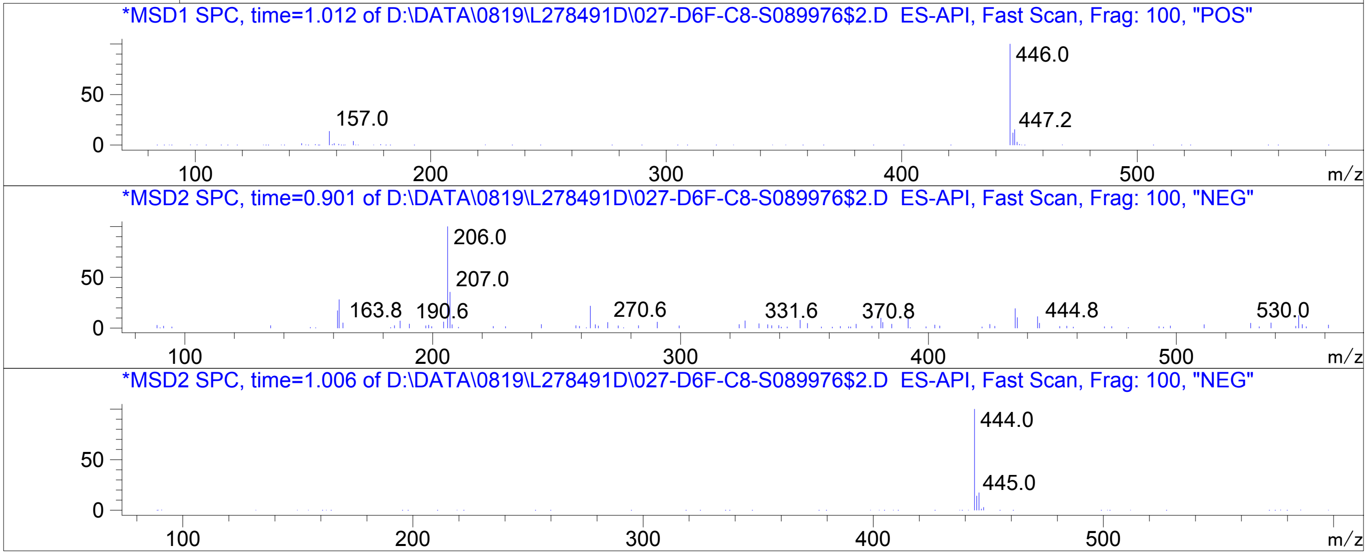

Inj.Date 8/18/2020 M Acq. Method C:\Users\ -> ->

H2565358

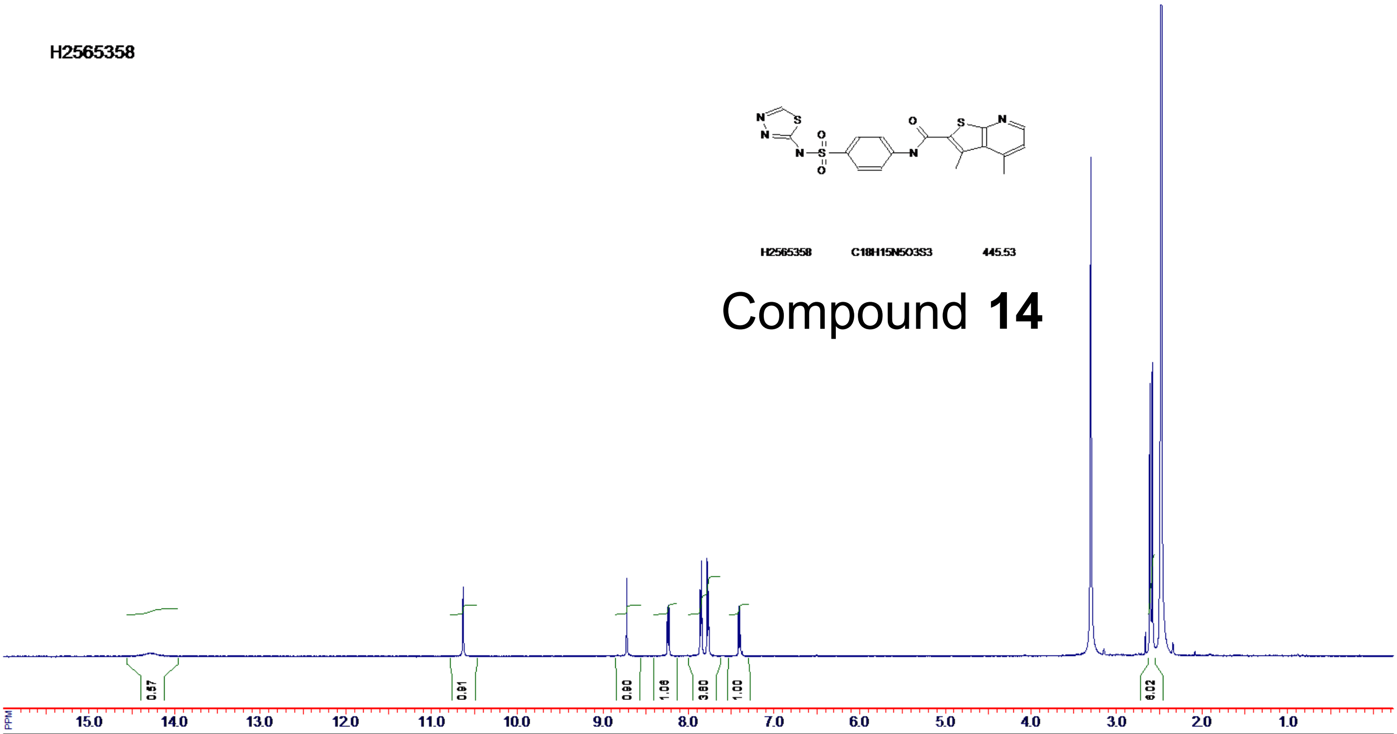

H2565358 C18H15N5O3S3 445.53

Compound **14**

File name: H2565358	Honchar	SF: 499.8207 MHz	NSC: 0	PW: 6.60 usec, RG: 40	St: 131072
Date: 01-Dec-2020	Solvent: dmso	SW: 11364 Hz	TE: 298 K	AQ: 2.82 sec, RD: 0.00 sec	H2565358

Compound **15**

S491337$21

MaxPeak: 95.91%
Ret_Time: 1.070 min

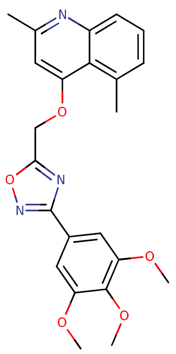

Mol Wt 421.45

Exact Mass 421.18

#	Time	Area%
1	0.865	1.97
2	1.005	2.12
3	1.070	95.91

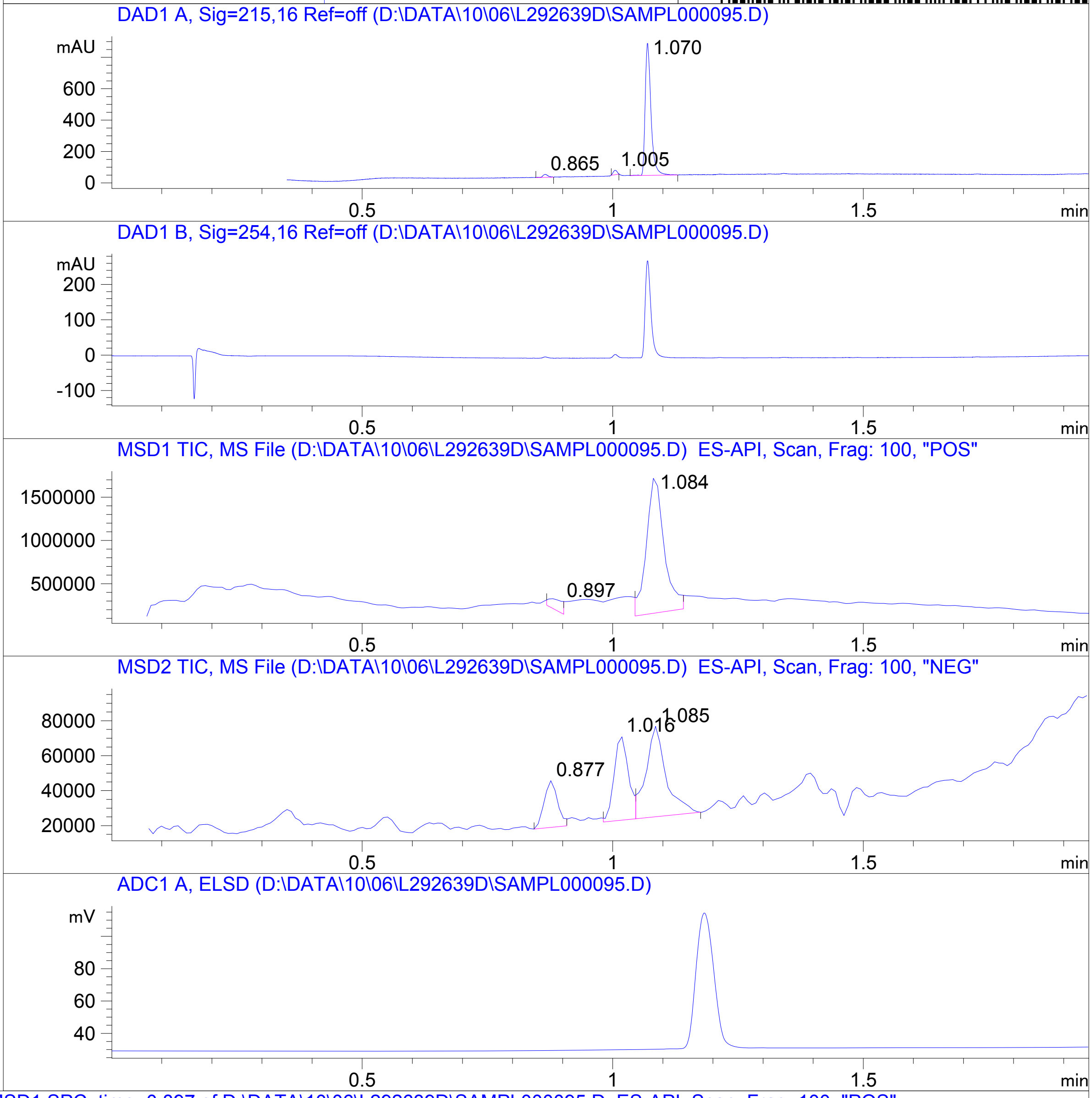

RT 0.897

RT 1.084

RT 0.877

RT 1.016

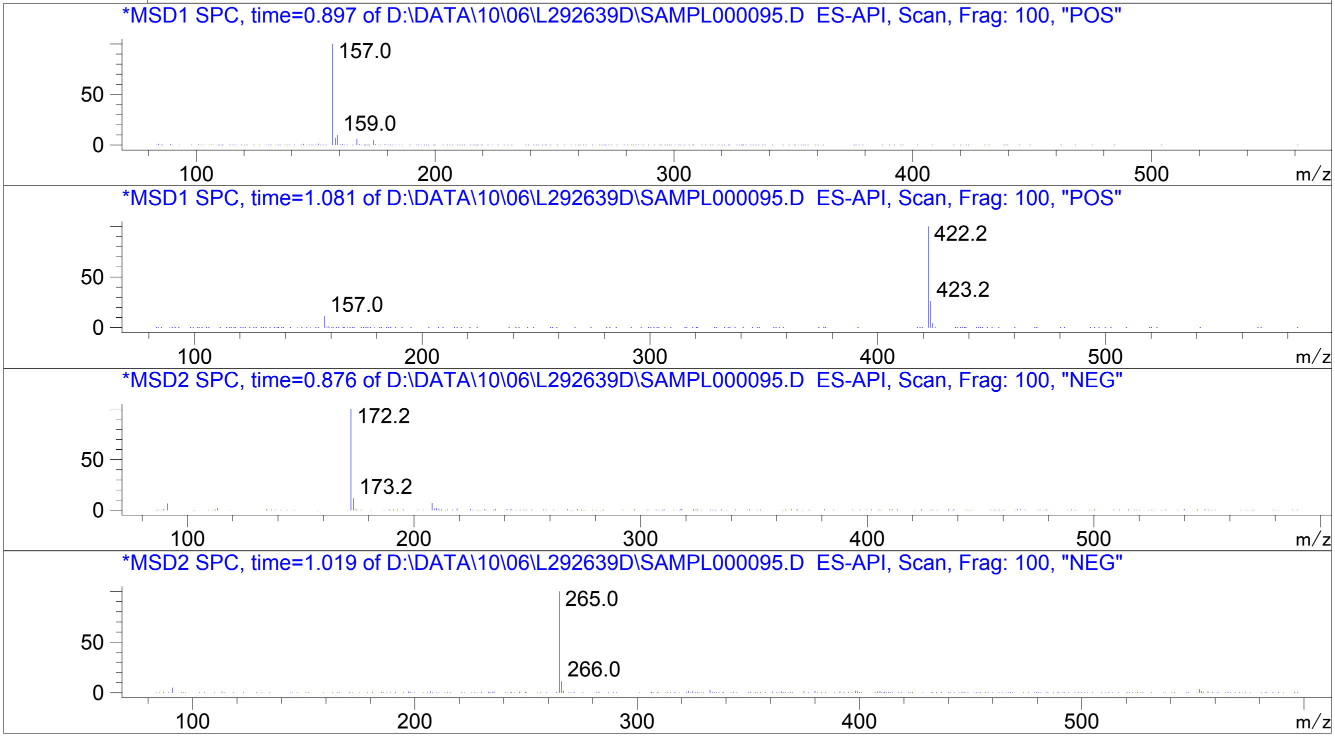

RT 1.085

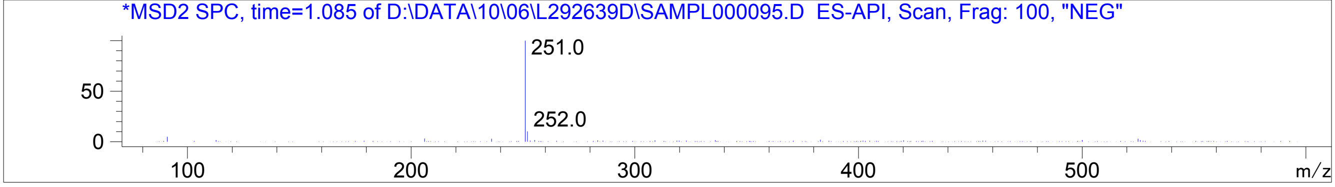
*MSD2 SPC, time=1.085 of D:\DATA\10\06\L292639D\SAMPL000095.D ES-API, Scan, Frag: 100, "NEG"
251.0
252.0
50
0
100
200
300
400
500
m/z

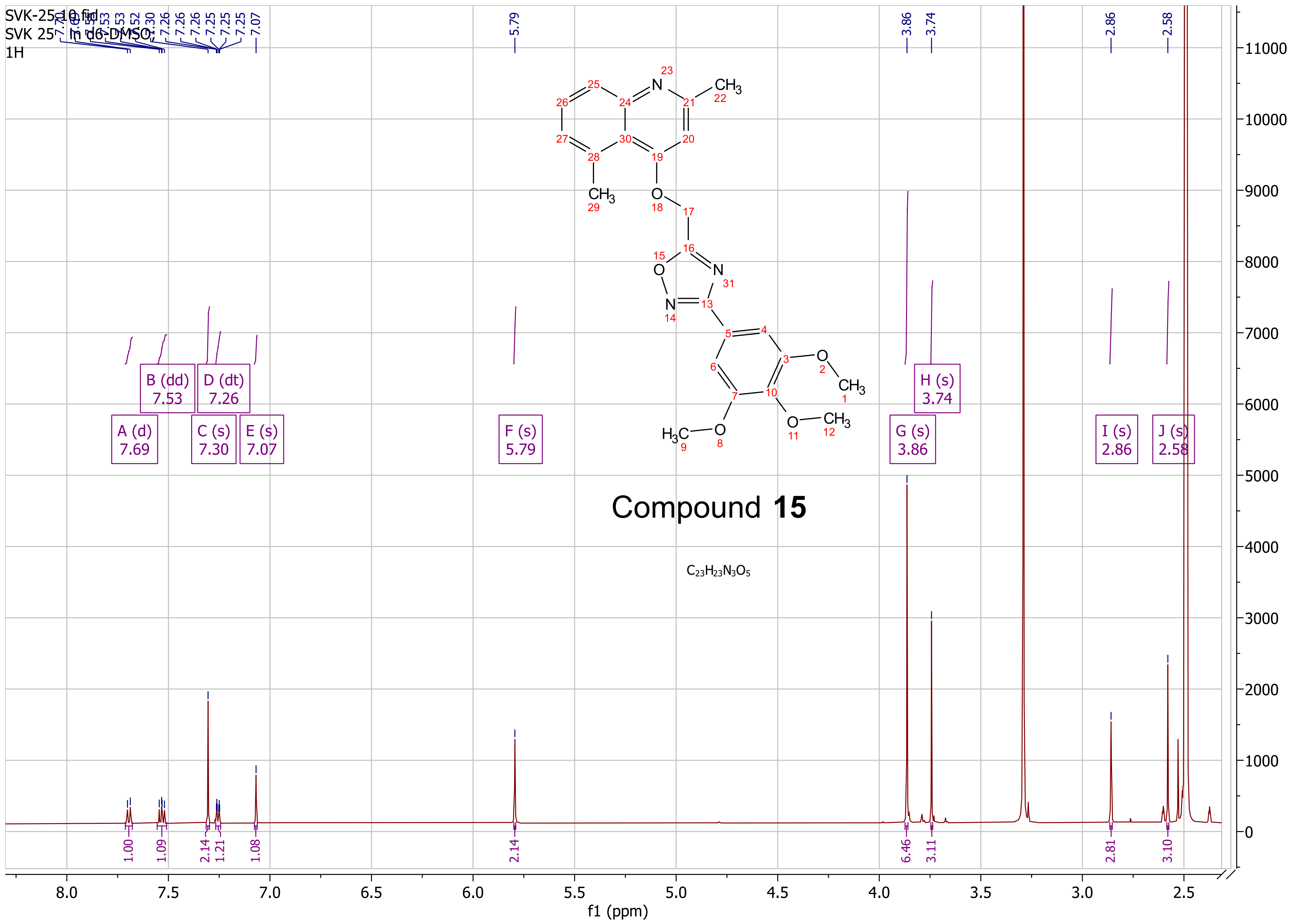

SVK-25-10.fid
SVK 25 in d6-DMSO;
1H
7.70
7.69
7.53
7.53
7.53
7.52
7.30
7.26
7.26
7.26
7.25
7.25
7.25
7.07
5.79
3.86
3.74
2.86
2.58
A (d) 7.69
B (dd) 7.53
C (s) 7.30
D (dt) 7.26
E (s) 7.07
F (s) 5.79
G (s) 3.86
H (s) 3.74
I (s) 2.86
J (s) 2.58
1.00
1.09
2.14
1.21
1.08
2.14
6.46
3.11
2.81
3.10
Compound 15
$C_{23}H_{23}N_3O_5$
f1 (ppm)

Compound **16**

Sample H2672083

0.1%FA-ACN 5-95%ACN 6 min. Zorbax-Eclipse-Plus C18 4,6 x 100mm, 3.5 mkm.

#	Time	Area%
1	5.827	100.00

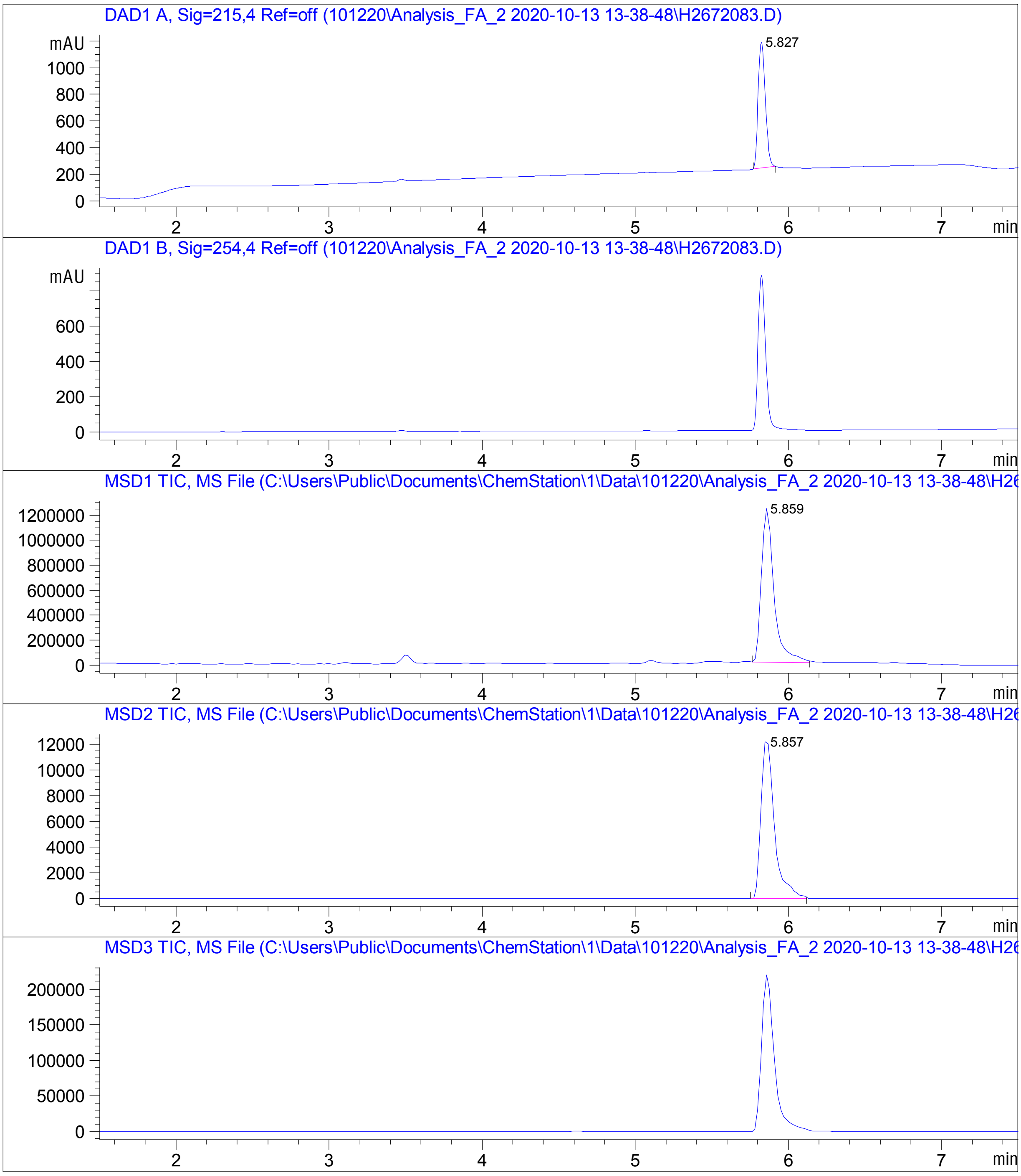

RT 5.859

RT 5.857

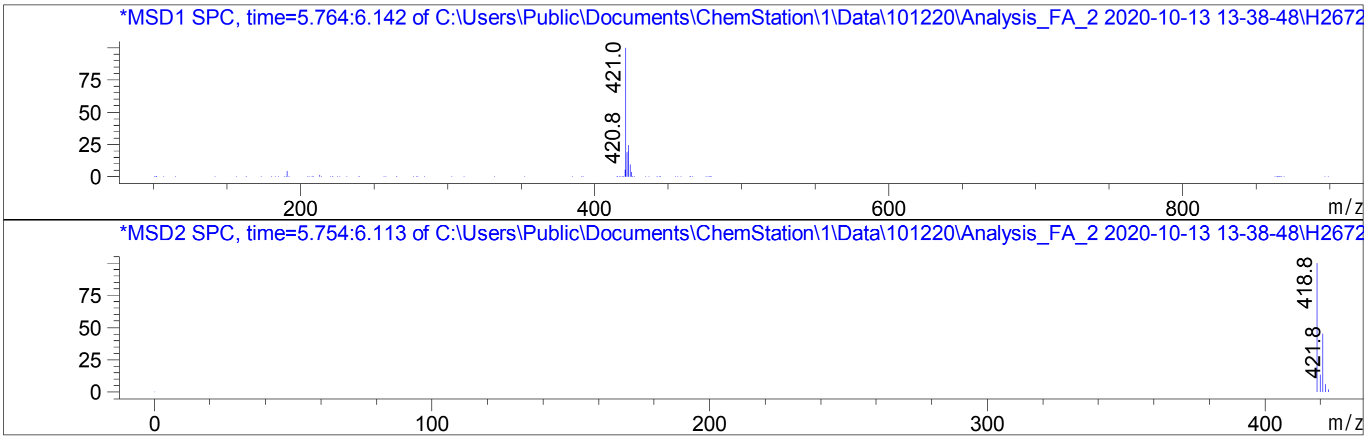

Tue, 13.->13.10.2020 S-> E D1F-B7 Acq. Method C:\Users\ ->

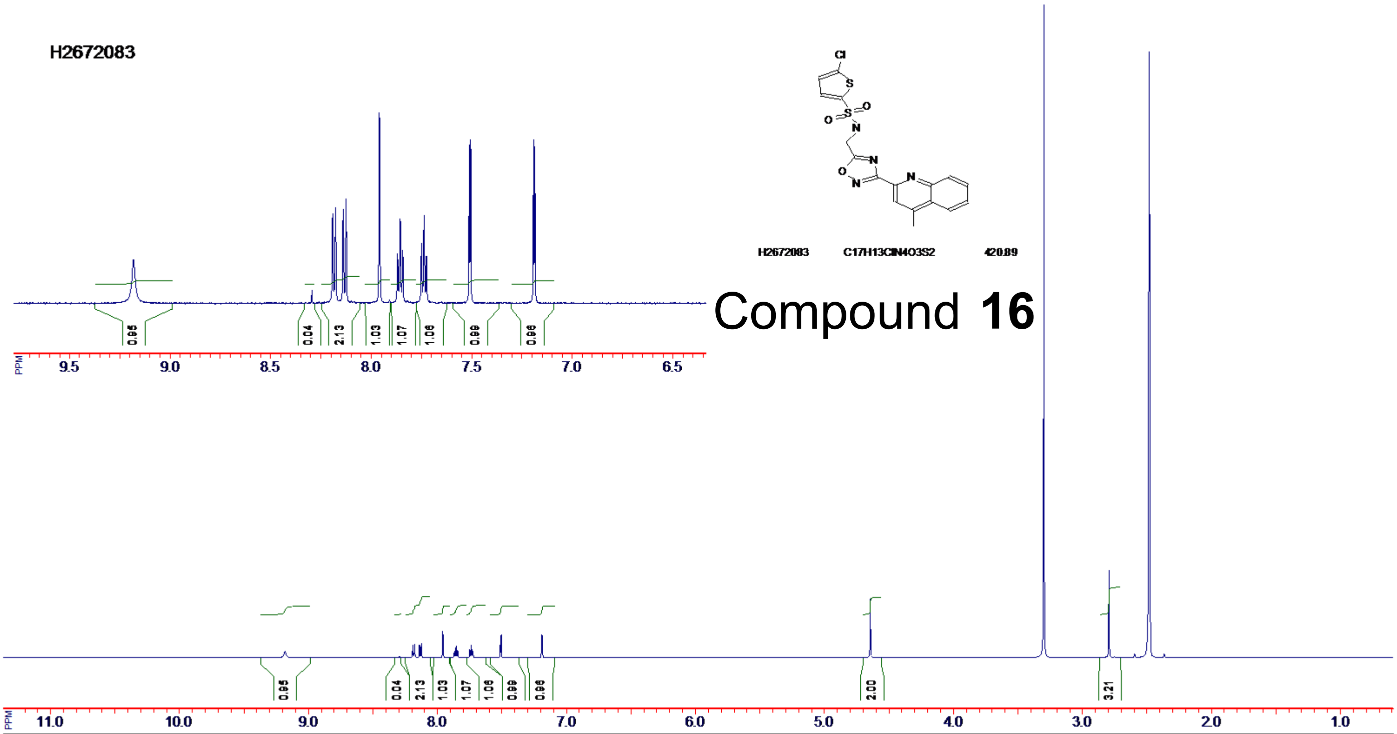

File name: H2672083	Honchar	SF: 599.7811 MHz	NSC: 0	PW: 4.40 usec, RG: 44	St: 131072
Date: 26-Nov-2020	Solvent: dmso	SW: 11364 Hz	TE: 298 K	AQ: 2.82 sec, RD: 0.00 sec	H2672083

Compound **17**

Sample H2665601

0.1%FA-ACN 5-95%ACN 6 min. Zorbax-Eclipse-Plus C18 4,6 x 100mm, 3.5 mkm.

#	Time	Area%
1	2.394	1.75
2	6.730	98.25

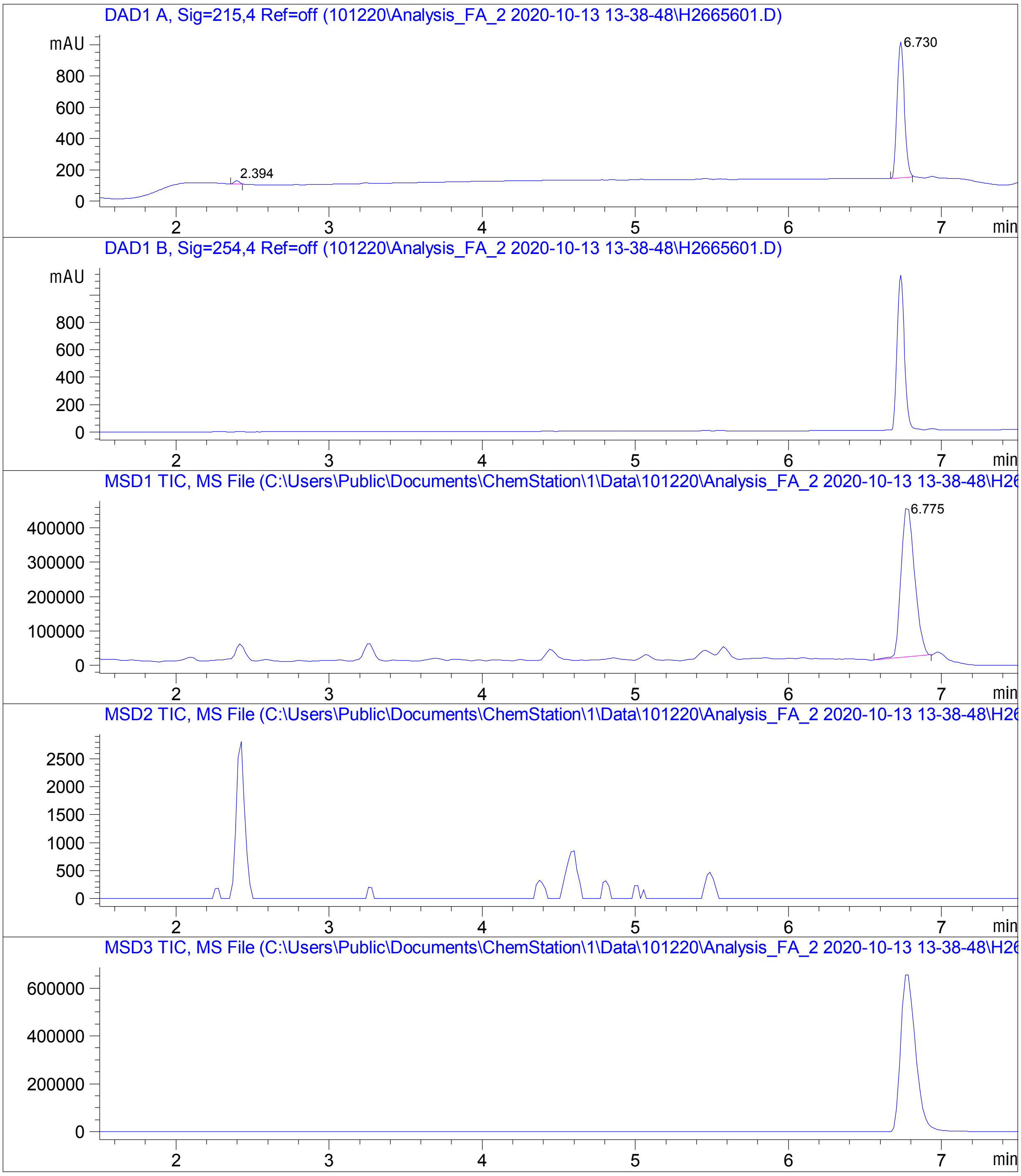

RT 6.775

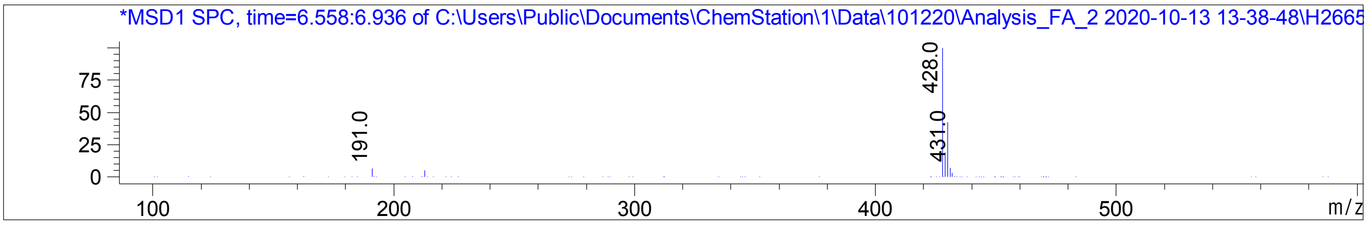

Tue, 13.->13.10.2020 S-> E D1F-A6 Acq. Method C:\Users\ ->

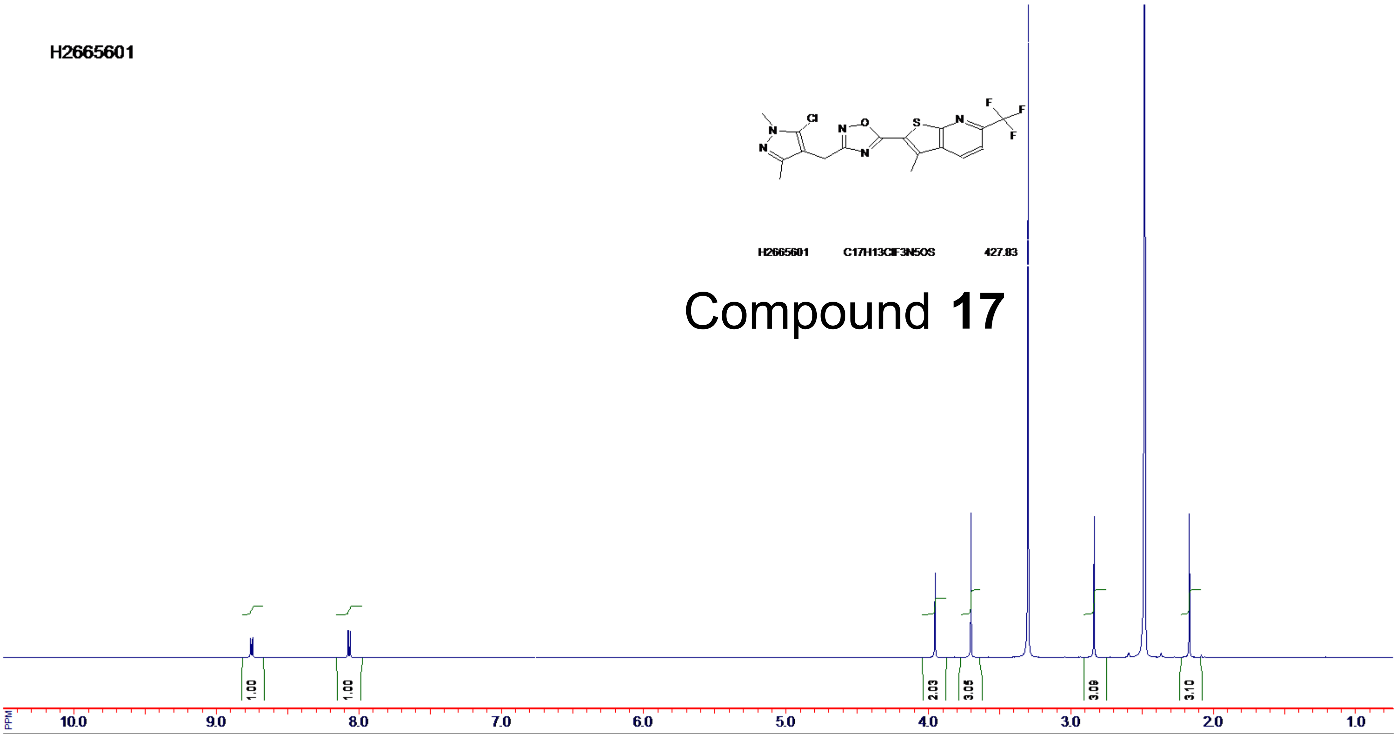

Compound **17**

File name: H2665601	Honchar	SF: 599.7811 MHz	NSC: 0	PW: 4.40 usec, RG: 44	St: 131072
Date: 26-Nov-2020	Solvent: dmso	SW: 11364 Hz	TE: 298 K	AQ: 2.82 sec, RD: 0.00 sec	H2665601

Compound **18**

Sample H2660982 0.1%FA-ACN 5-95%ACN 6 min. Zorbax-Eclipse-Plus C18 4,6 x 100mm, 3.5 mkm.

#	Time	Area%
1	6.442	100.00

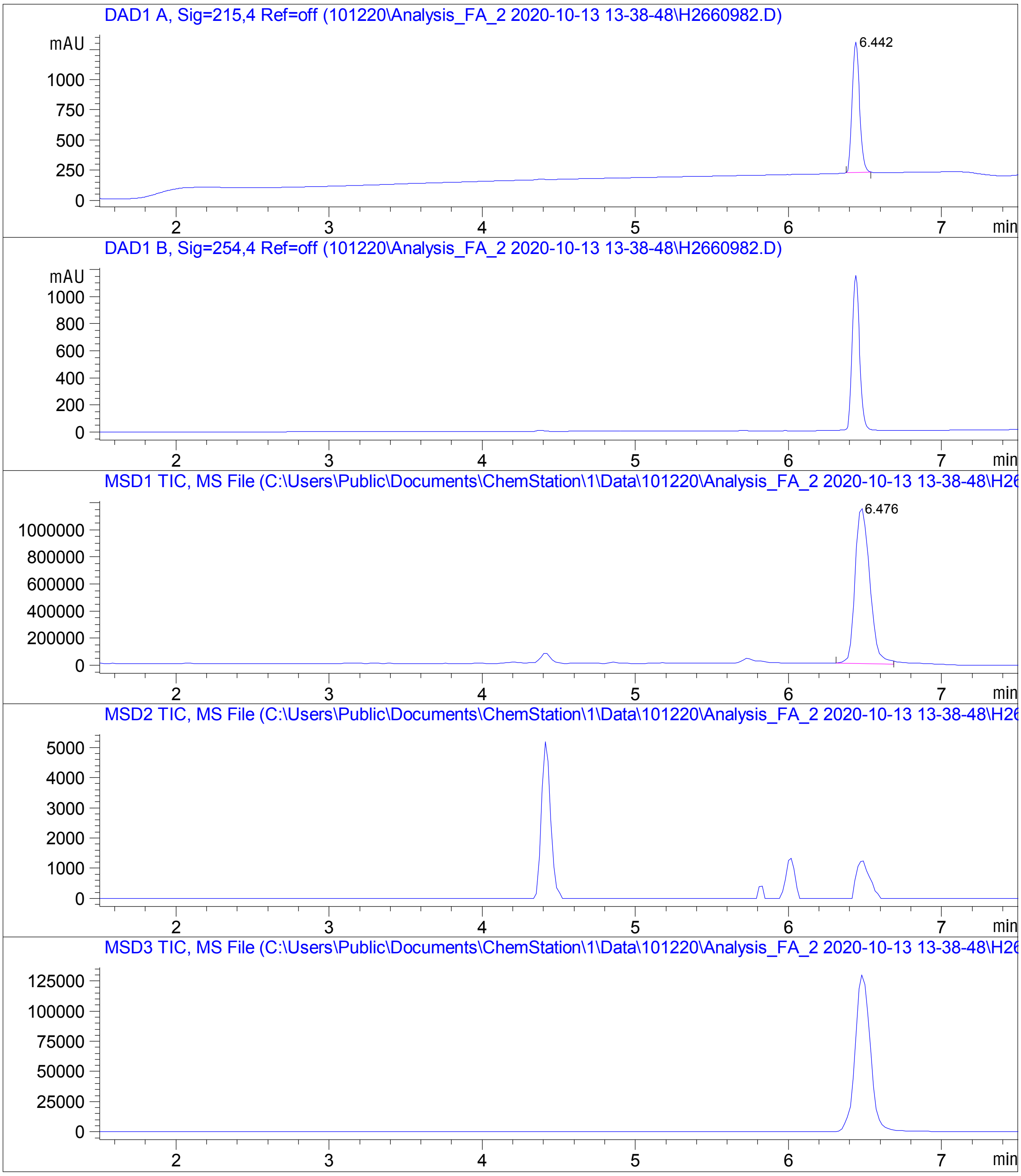

RT 6.476

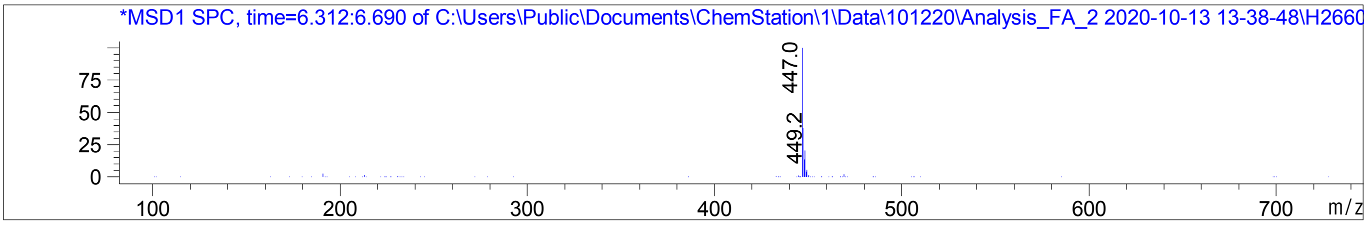

Tue, 13.->13.10.2020 S-> E D1F-B3 Acq. Method C:\Users\ ->

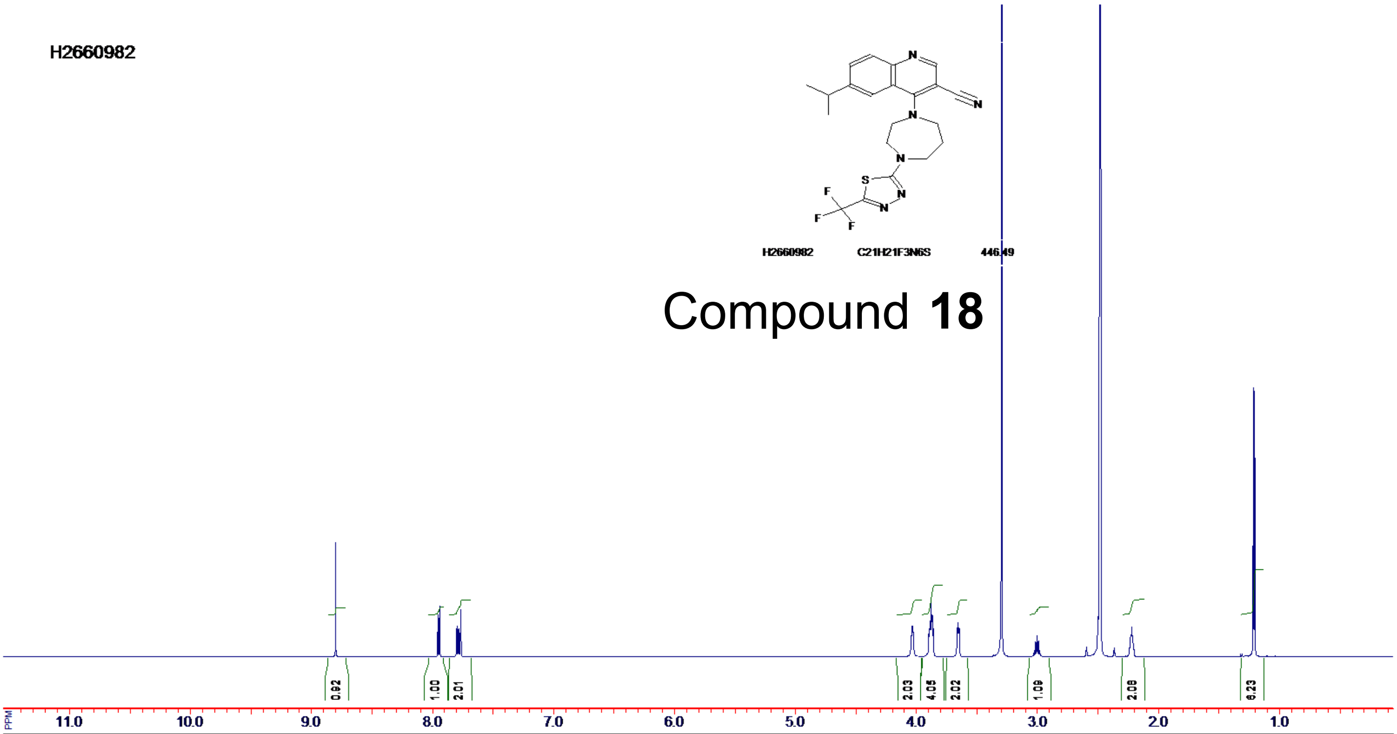

Compound **18**

File name: H2660982	Honchar	SF: 599.7811 MHz	NSC: 0	PW: 4.40 usec, RG: 44	SI: 131072
Date: 26-Nov-2020	Solvent: dmso	SW: 11364 Hz	TE: 298 K	AQ: 2.82 sec, RD: 0.00 sec	H2660982

Compound **19**

Sample H2666177

0.1%FA-ACN 5-95%ACN 6 min. Zorbax-Eclipse-Plus C18 4,6 x 100mm, 3.5 mkm.

#	Time	Area%
1	4.378	0.28
2	5.416	99.72

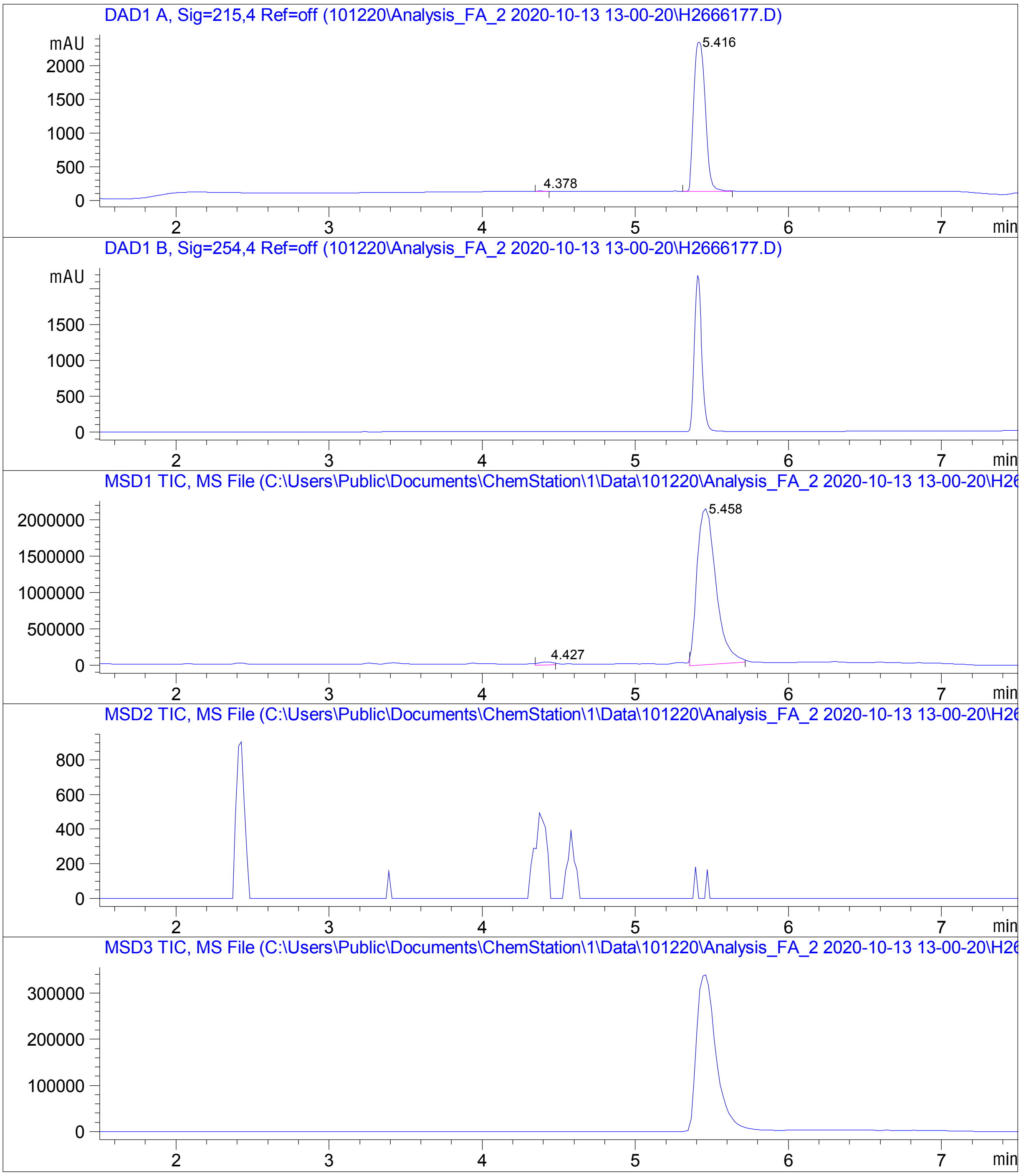

RT 4.427

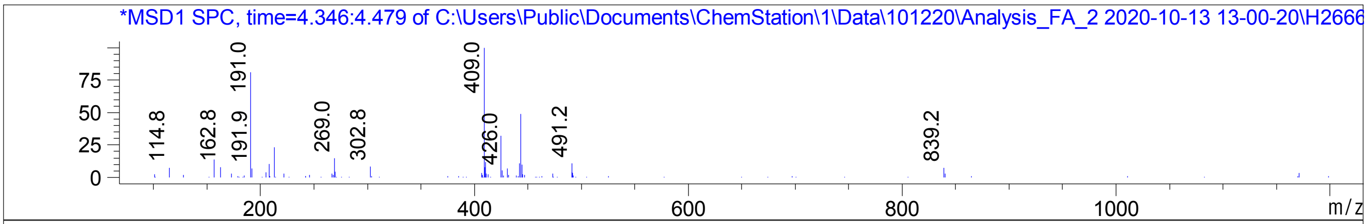

RT 5.458

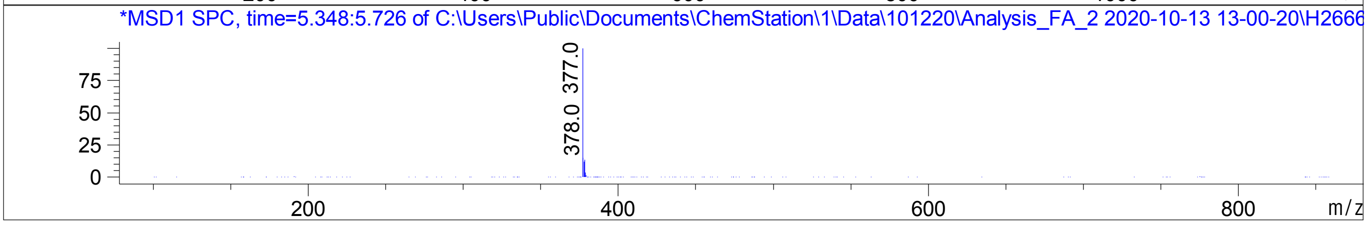

Tue, 13.->13.10.2020 S-> E D1F-A4 Acq. Method C:\Users\ ->

H2666177

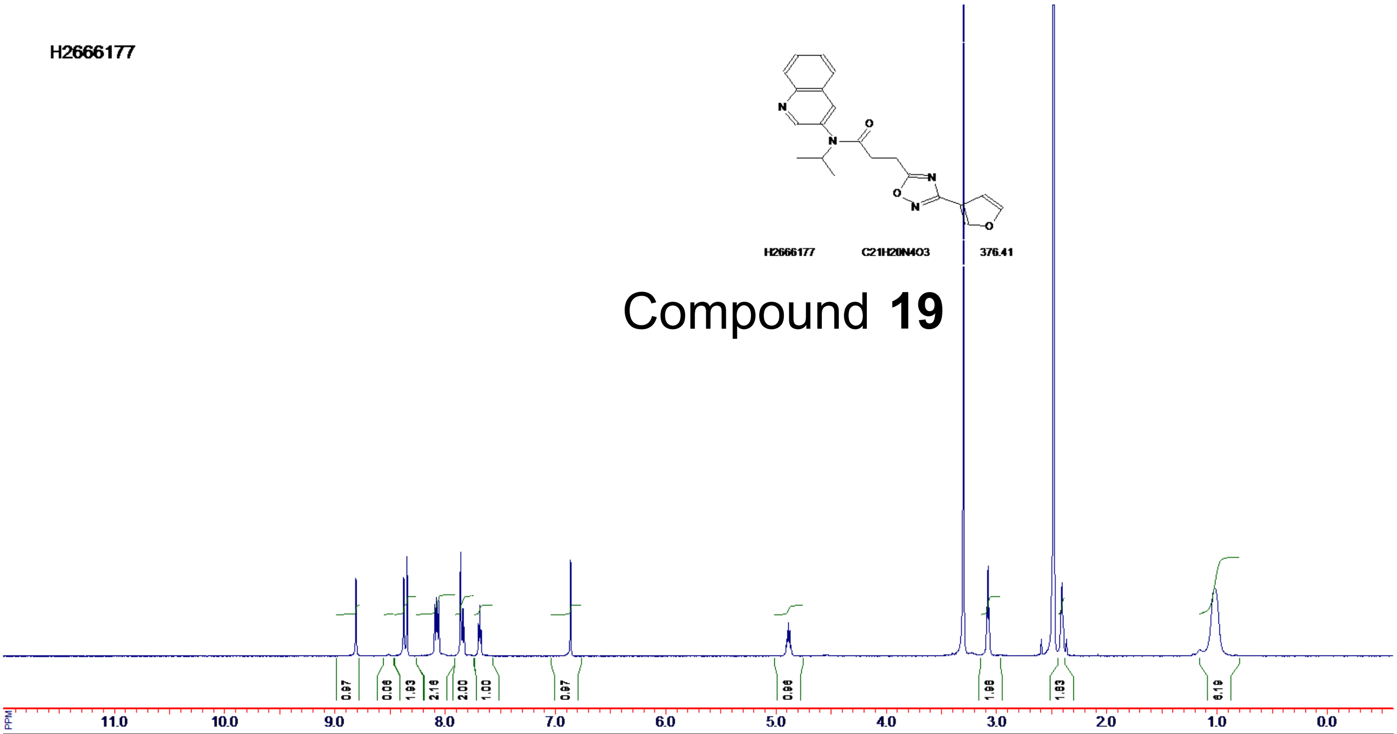

Compound **19**

File name: H2666177	Honchar	SF: 599.7811 MHz	NSC: 0	PW: 4.40 usec, RG: 44	SI: 131072
Date: 26-Nov-2020	Solvent: dmso	SW: 11364 Hz	TE: 298 K	AQ: 2.82 sec, RD: 0.00 sec	H2666177

Compound **20**

S089986$3

MaxPeak: 100.00%
Ret_Time: 1.248 min

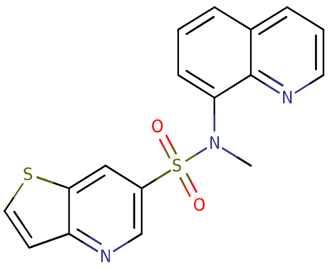

Mol Wt 355.43
Exact Mass 355.05

#	Time	Area%
1	1.248	100.00

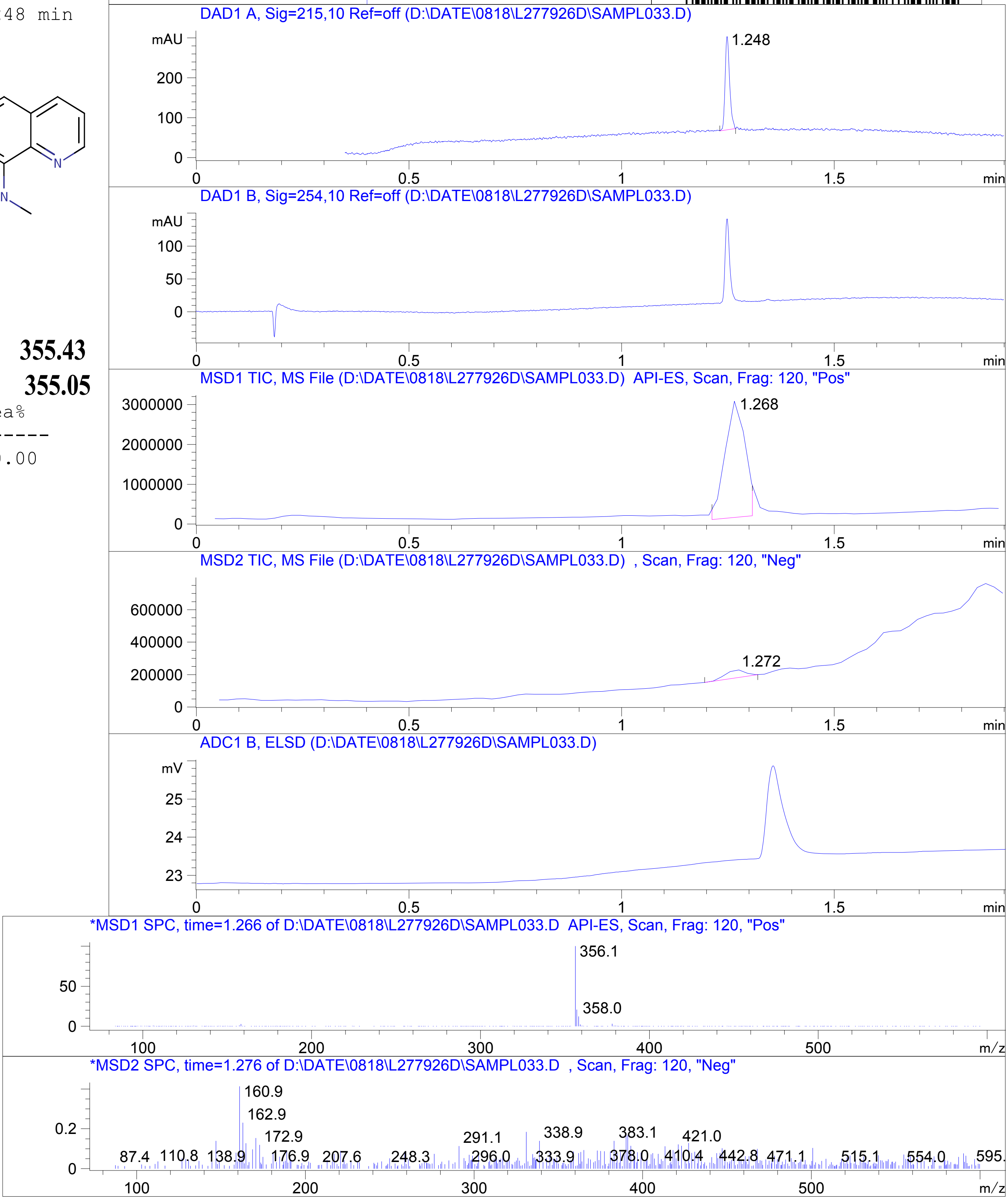

RT 1.268

RT 1.272

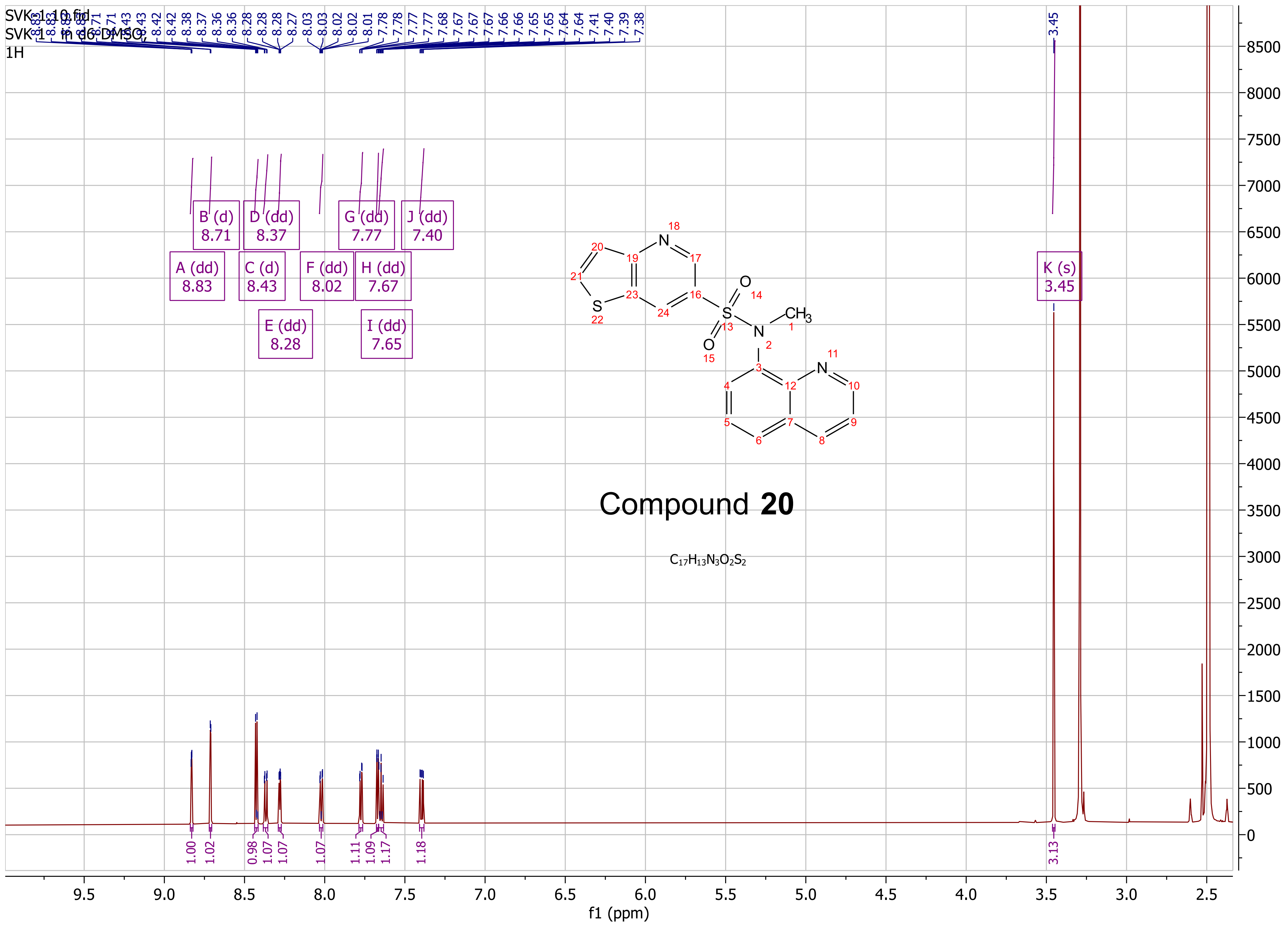

Compound **20**

$C_{17}H_{13}N_3O_2S_2$

Compound **21**

MaxPeak: 98.57%
Ret_Time: 1.513 min

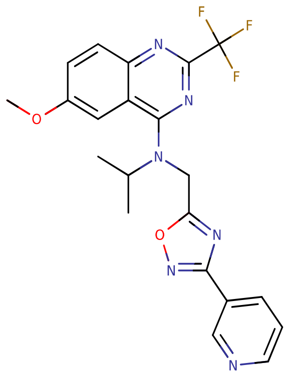

Mol Wt 444.41
Exact Mass 444.17

#	Time	Area%
1	1.475	1.43
2	1.513	98.57

S089944$22

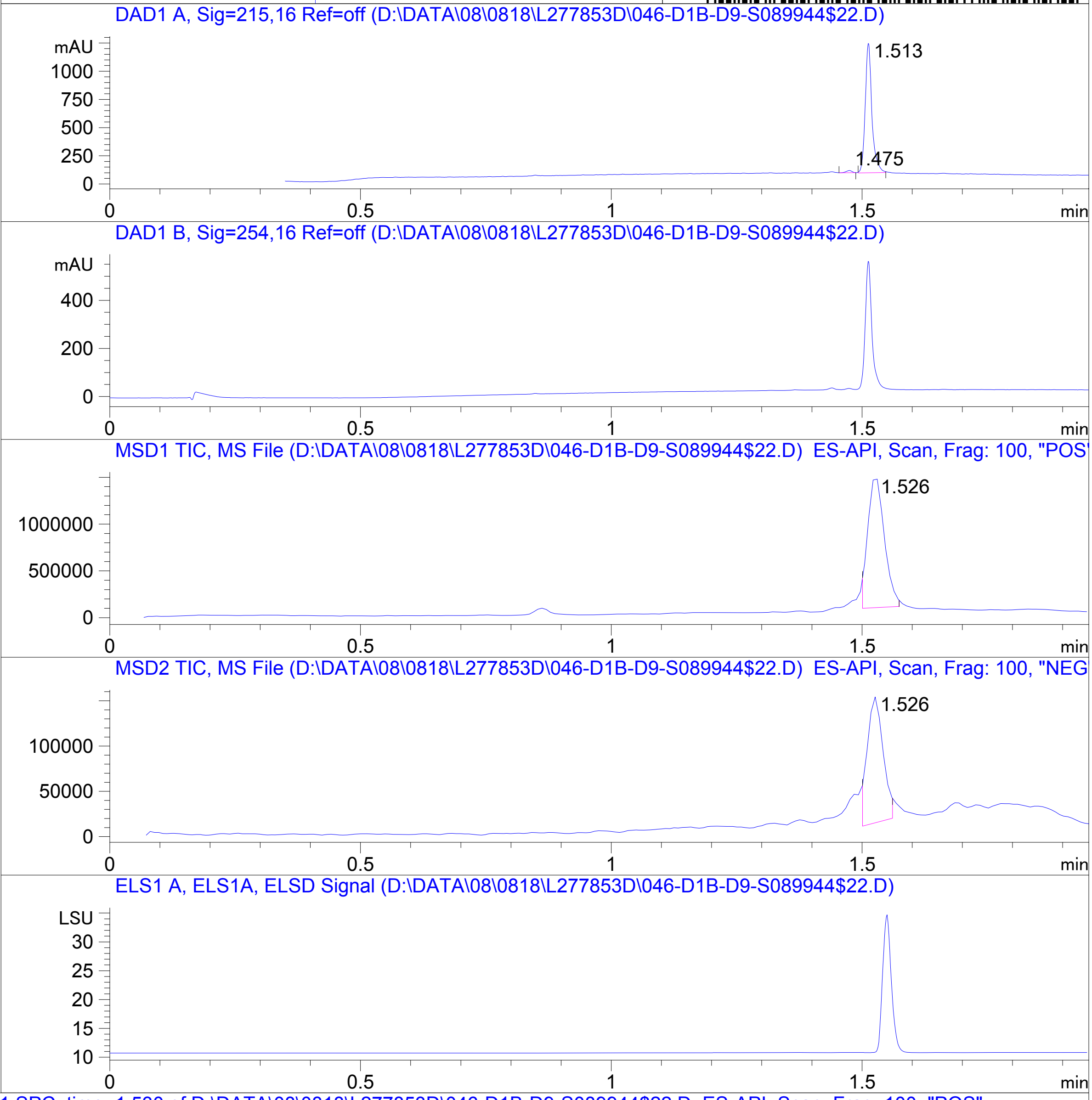

RT 1.526

RT 1.526

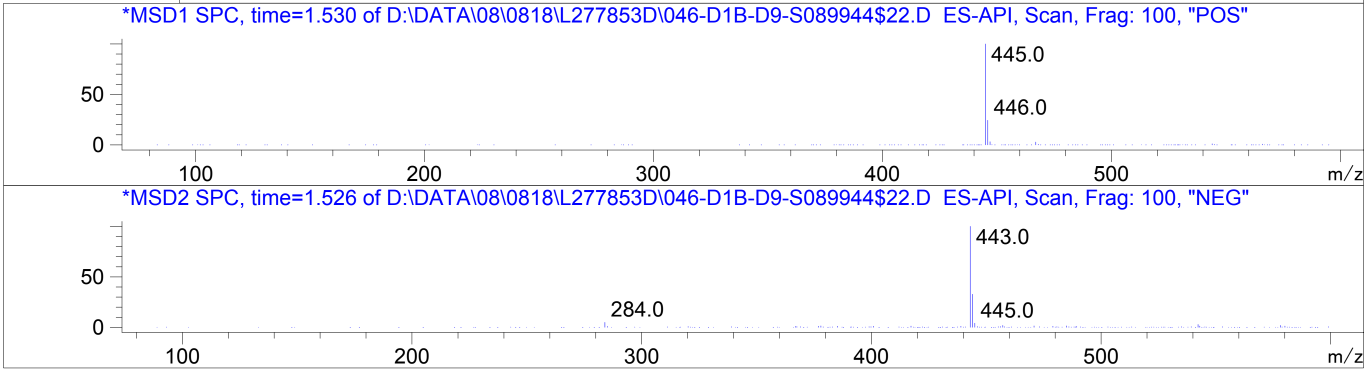

Inj.Date 8/17/2020 LT 13 Acq. Method C:\Chem32\-> ->

H2565658

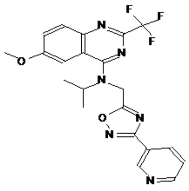

H2565658 $C_{21}H_{19}F_3N_6O_2$ 444.42

Compound **21**

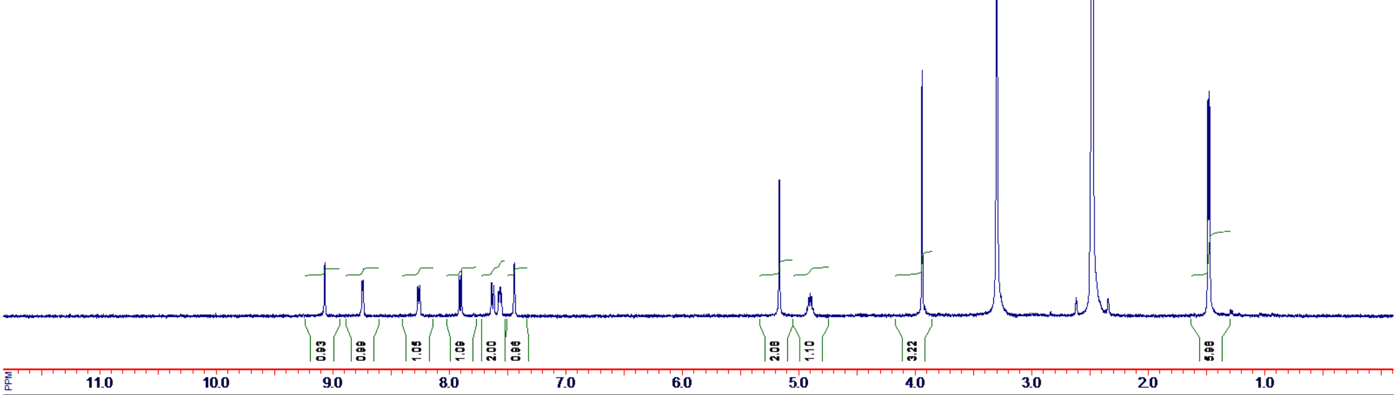

File name: H2565658	Honchar	SF: 499.8207 MHz	NSC: 0	PW: 6.60 usec, RG: 40	SI: 131072
Date: 01-Dec-2020	Solvent: dmso	SW: 11364 Hz	TE: 298 K	AQ: 2.82 sec, RD: 0.00 sec	H2565658

Compound **22**

MaxPeak: 100.00%
Ret_Time: 1.110 min

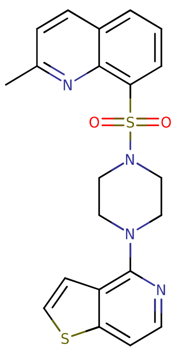

Mol Wt 424.54

Exact Mass 424.12

#	Time	Area%
1	1.110	100.00

S089988$5

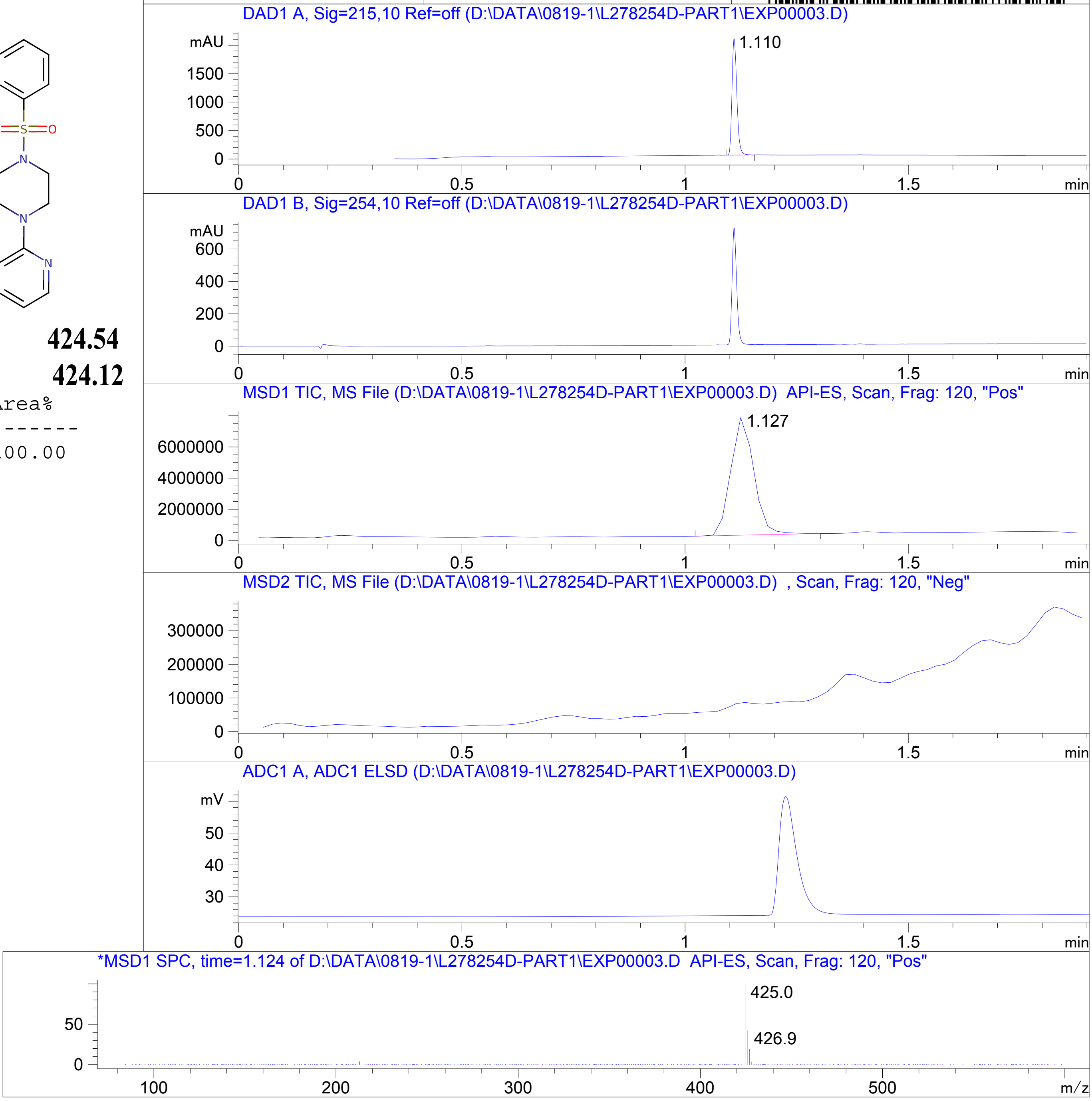

RT 1.127

Inj.Date 8/20/2020 M -VL- Acq. Method C:\HPCHEM\-> ->

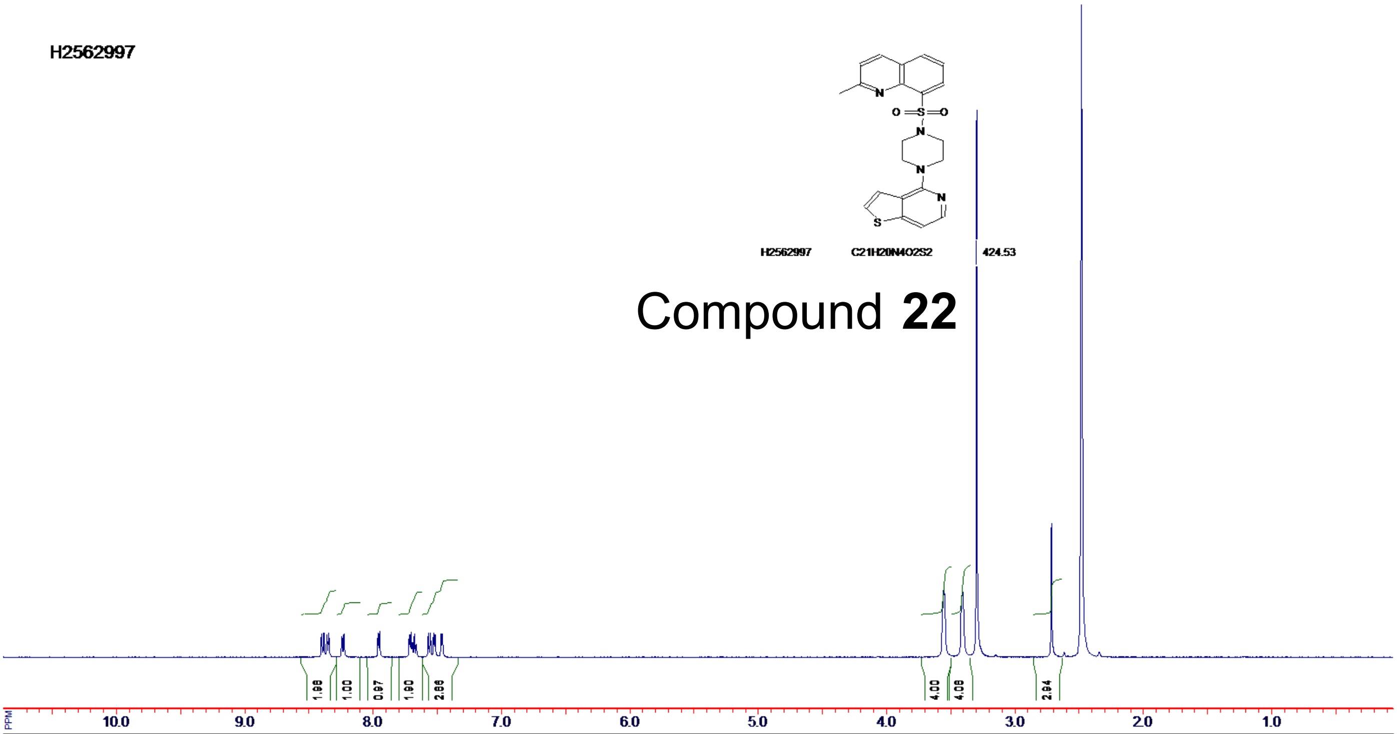

Compound **22**

File name: H2562997	Honchar	SF: 499.8207 MHz	NSC: 0	PW: 6.60 usec, RG: 40	St: 131072
Date: 01-Dec-2020	Solvent: dmso	SW: 11364 Hz	TE: 298 K	AQ: 2.82 sec, RD: 0.00 sec	H2562997

Compound 23

Sample H2660127 0.1%FA-ACN 5-95%ACN 6 min. Zorbax-Eclipse-Plus C18 4,6 x 100mm, 3.5 mkm.

#	Time	Area%
1	5.391	53.36
2	5.443	46.64

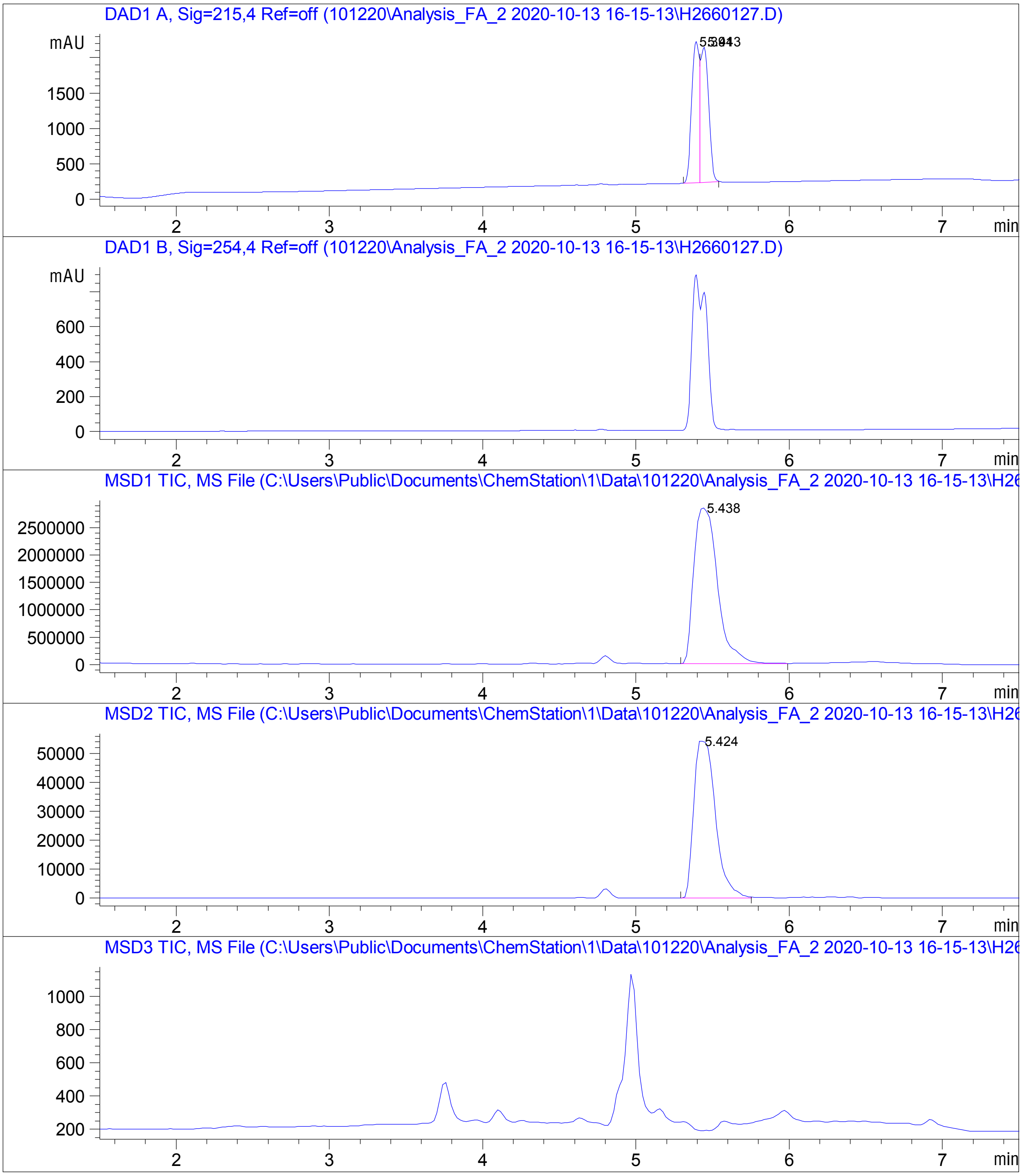

RT 5.438

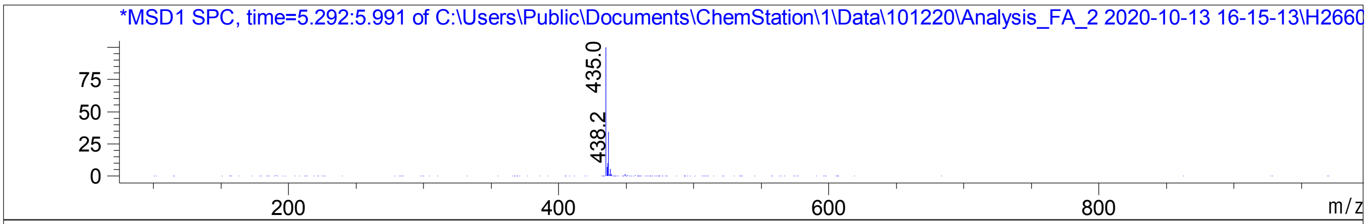

RT 5.424

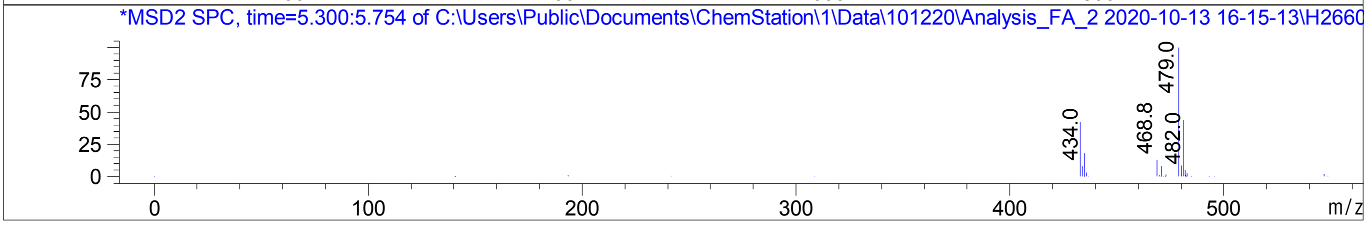

Tue, 13.->13.10.2020 S-> E D1F-B2 Acq. Method C:\Users\ ->

H2660127

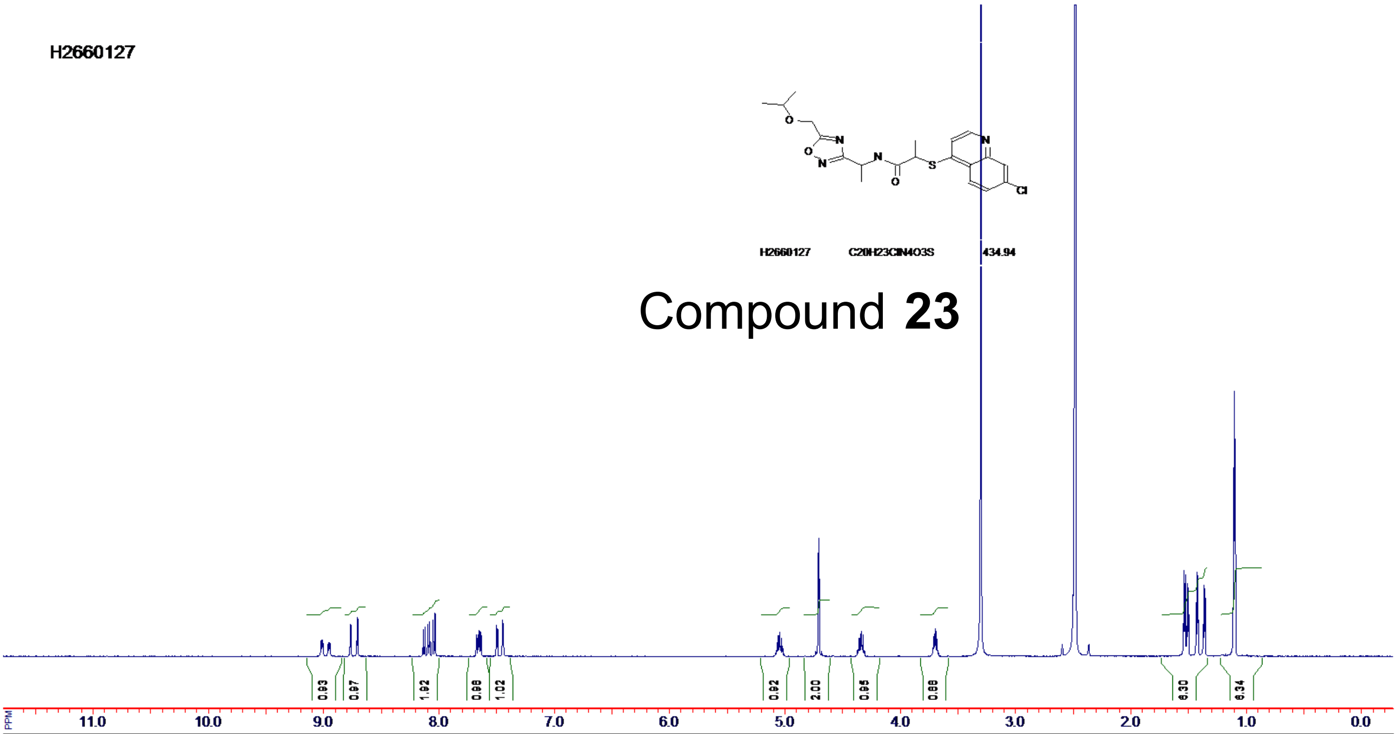

Compound **23**

File name: H2660127	Honchar	SF: 599.7811 MHz	NSC: 0	PW: 4.40 usec, RG: 44	SI: 131072
Date: 26-Nov-2020	Solvent: dmso	SW: 11364 Hz	TE: 298 K	AQ: 2.82 sec, RD: 0.00 sec	H2660127

Compound **24**

Sample H2572918 0.1%FA-ACN 5-95% ACN, 15 min. Kinetext C18 4,6 x 150mm, 2.6 u.

#	Time	Area%
1	6.489	98.38
2	7.207	0.45
3	8.529	0.70
4	8.772	0.27
5	9.330	0.20

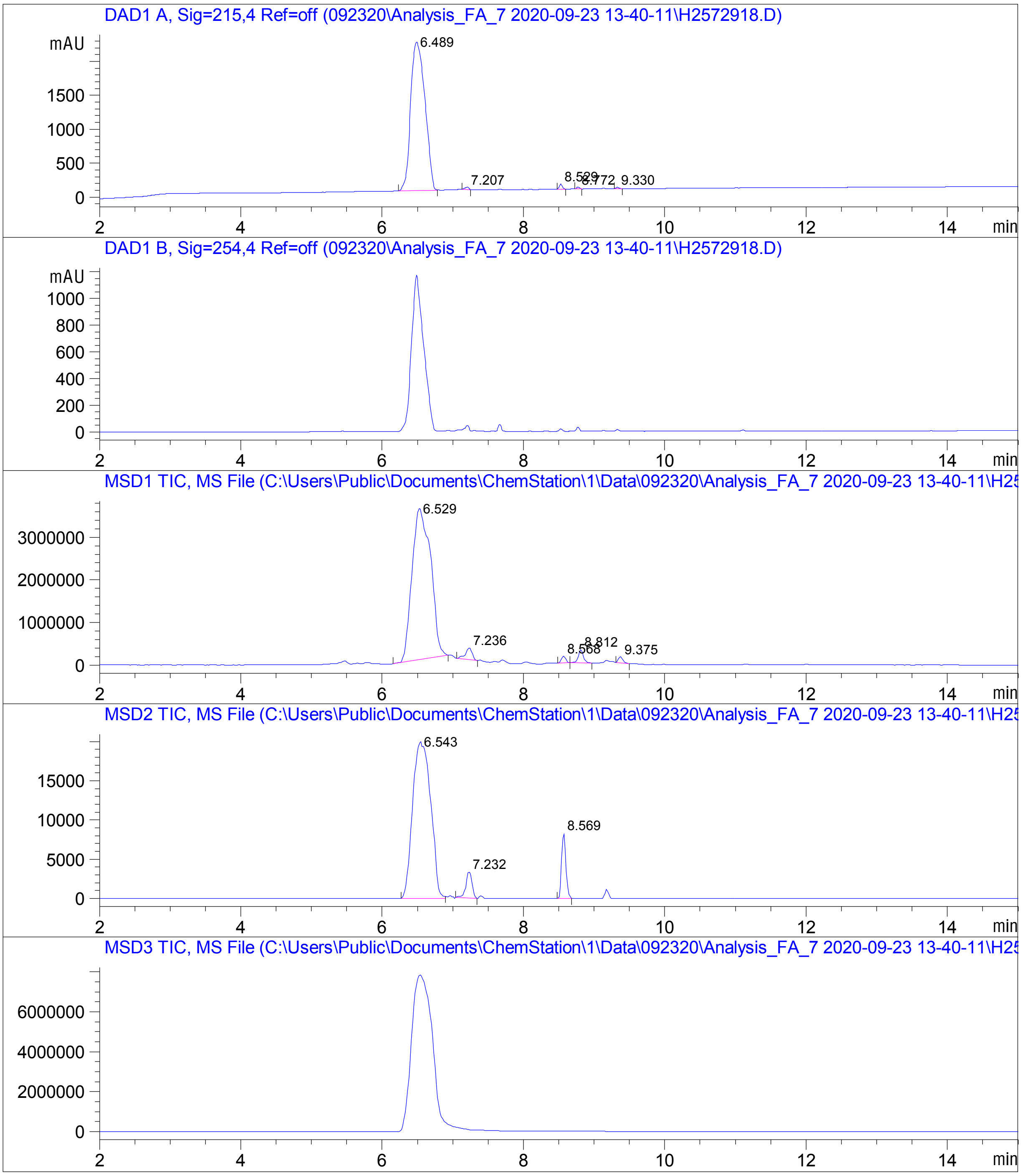

RT 6.529

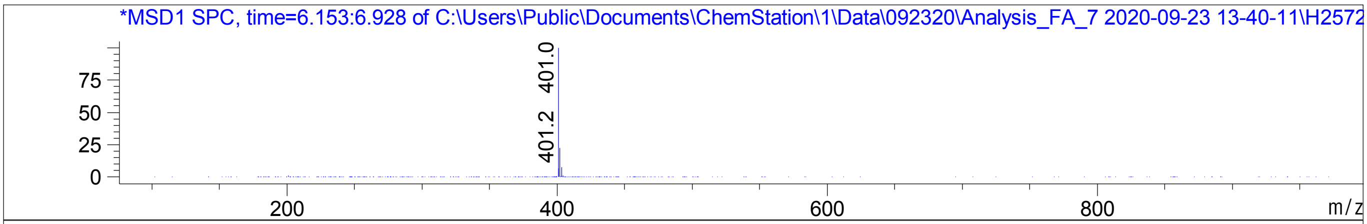

RT 7.236

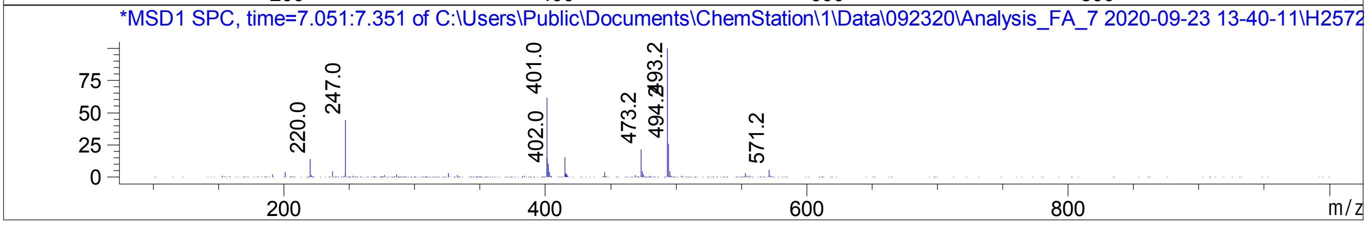

RT 8.568

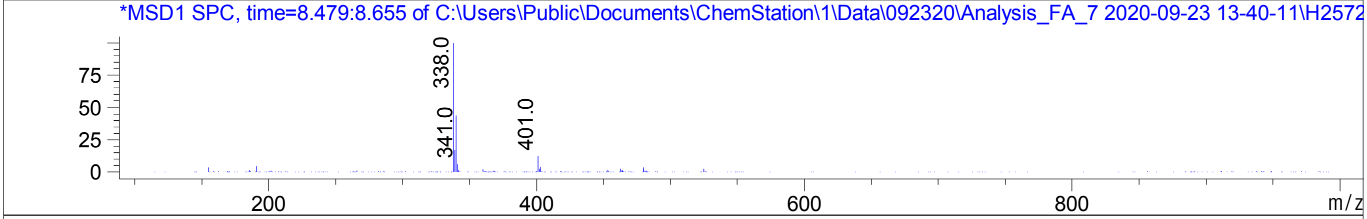

RT 8.812

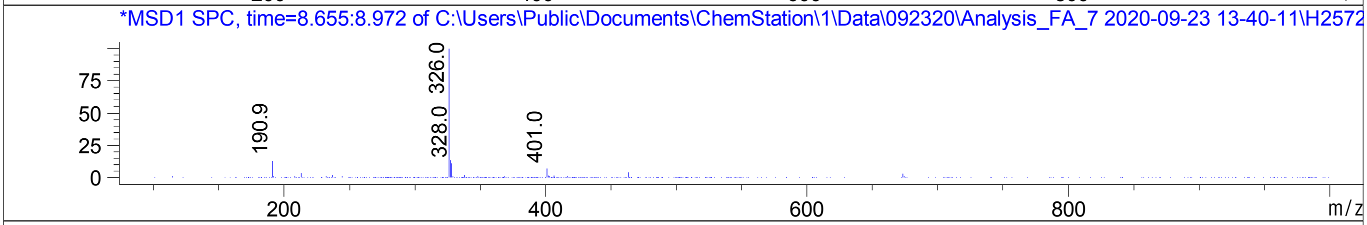

RT 9.375

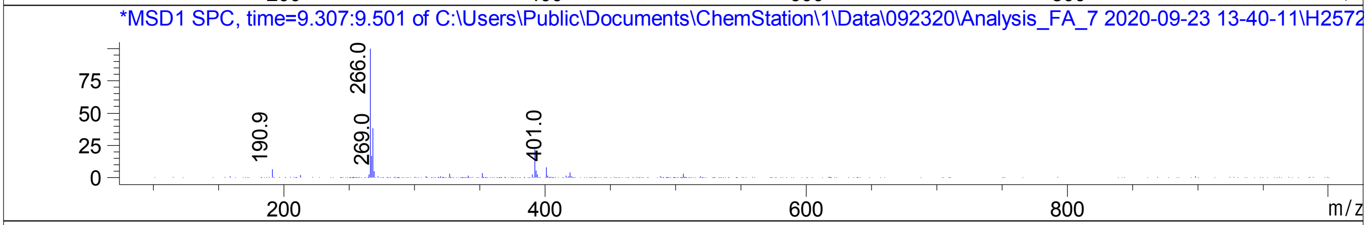

RT 6.543

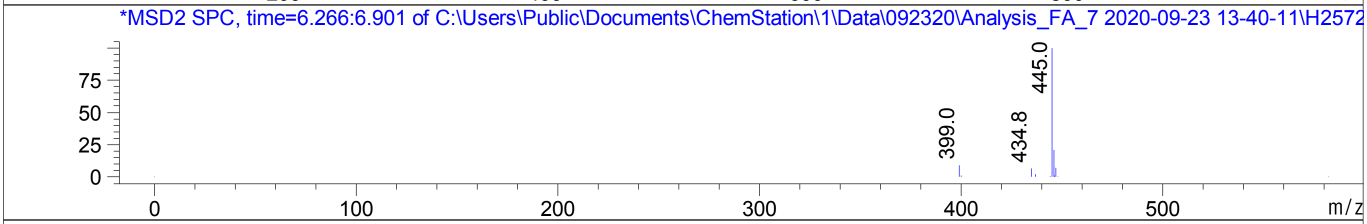

RT 7.232

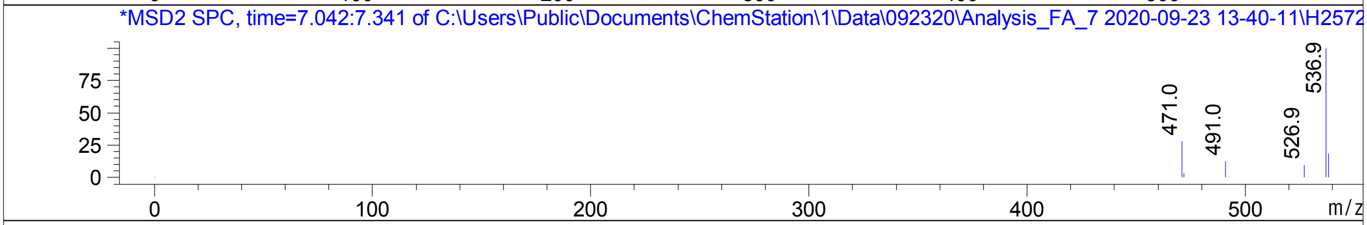

RT 8.569

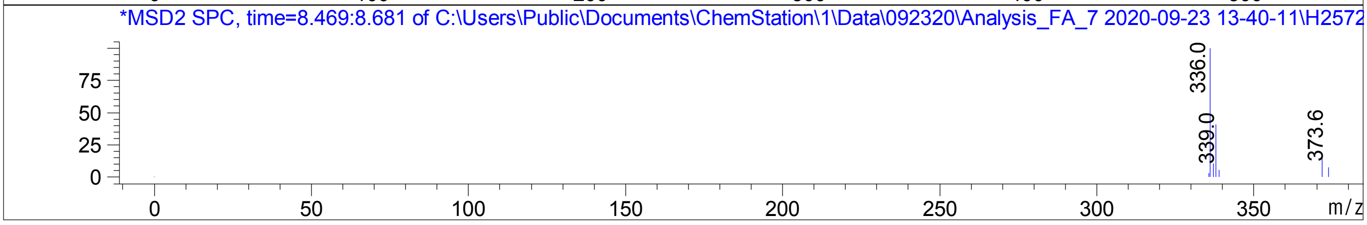

H2572918

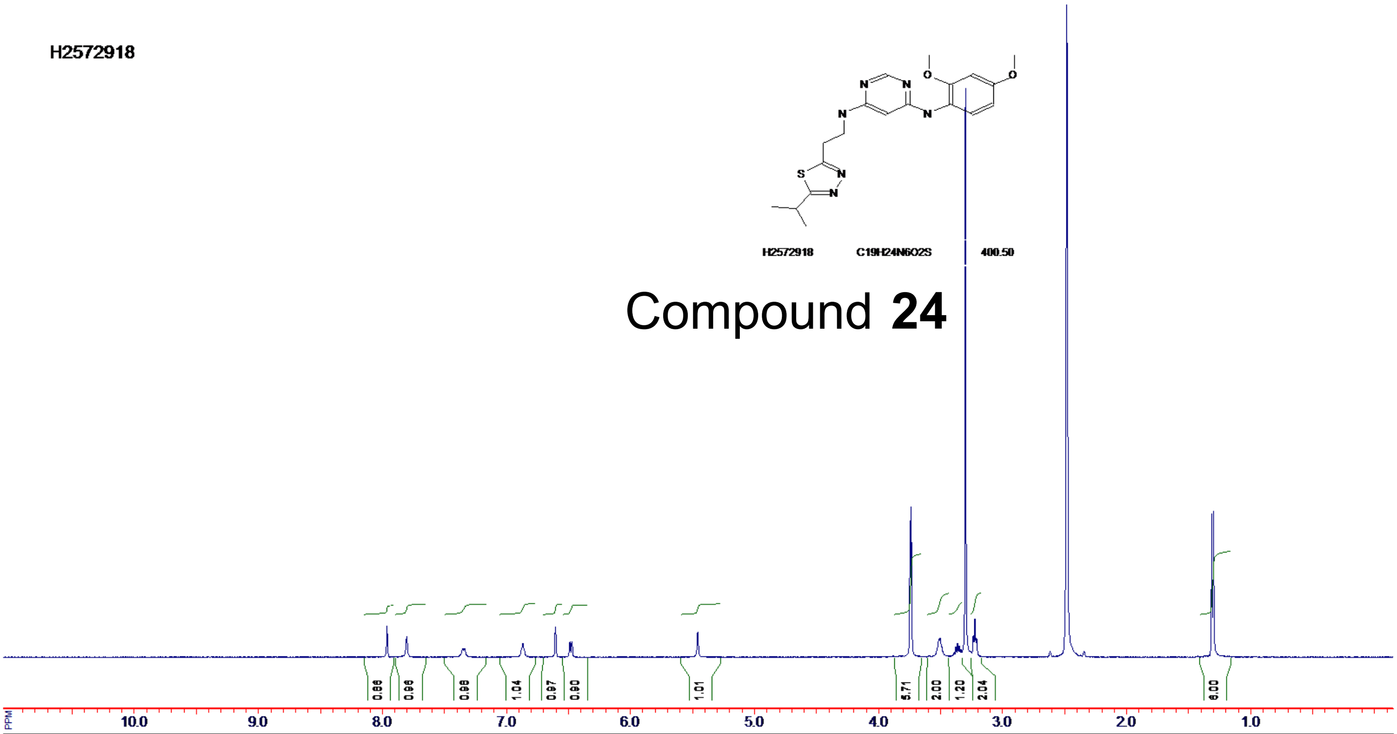

File name: H2572918	Honchar	SF: 499.8207 MHz	NSC: 0	PW: 6.60 usec, RG: 40	SI: 131072
Date: 01-Dec-2020	Solvent: dmso	SW: 11364 Hz	TE: 298 K	AQ: 2.82 sec, RD: 0.00 sec	H2572918

Compound 25

Sample H2663691

0.1%FA-ACN 5-95%ACN 6 min. Zorbax-Eclipse-Plus C18 4,6 x 100mm, 3.5 mkm.

#	Time	Area%
1	3.891	100.00

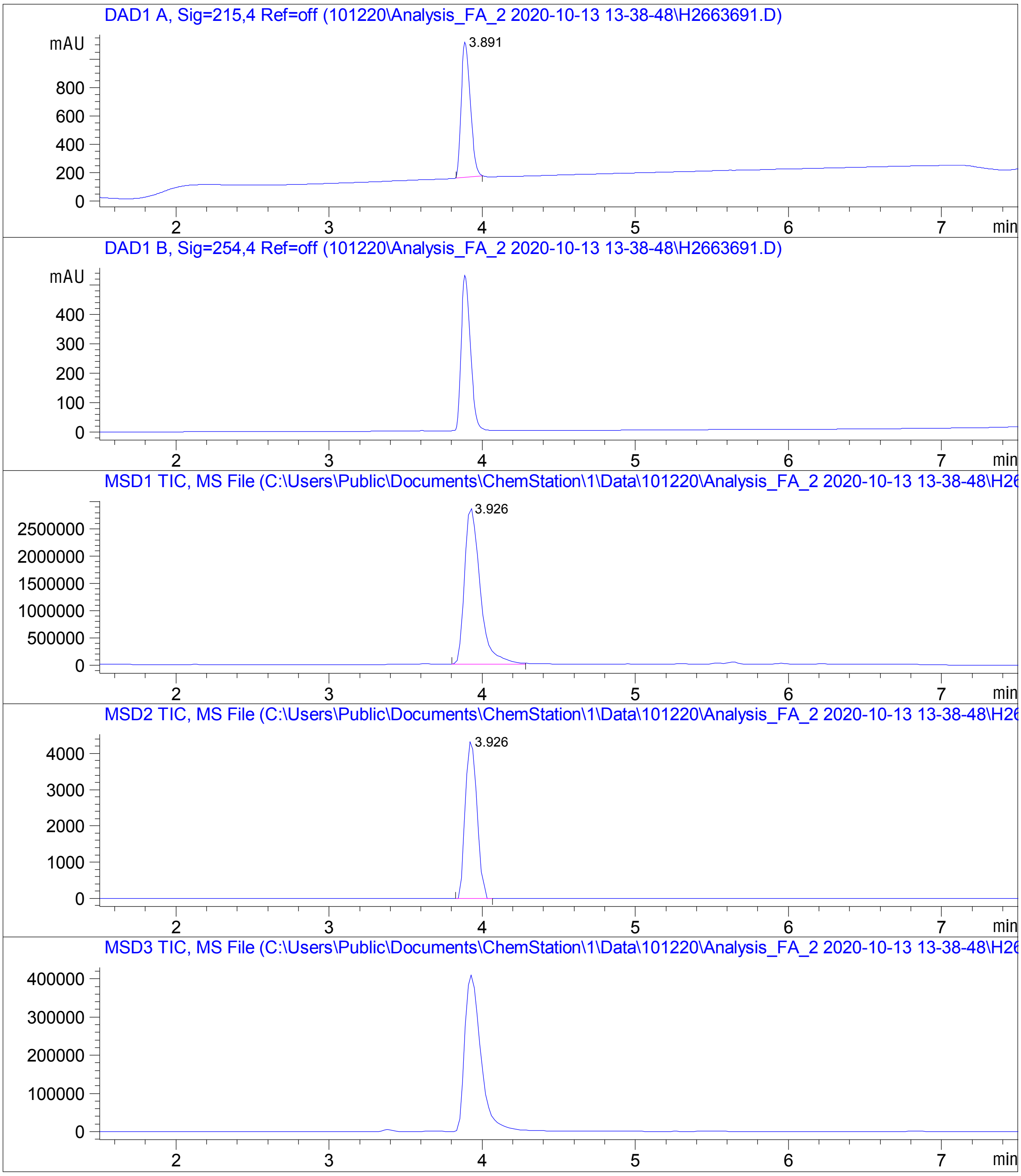

RT 3.926

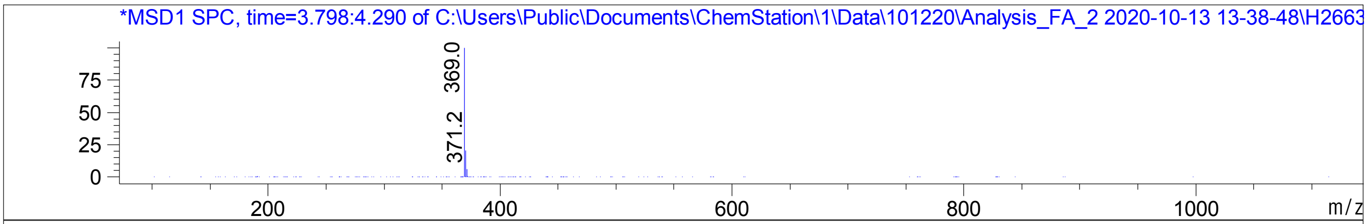

RT 3.926

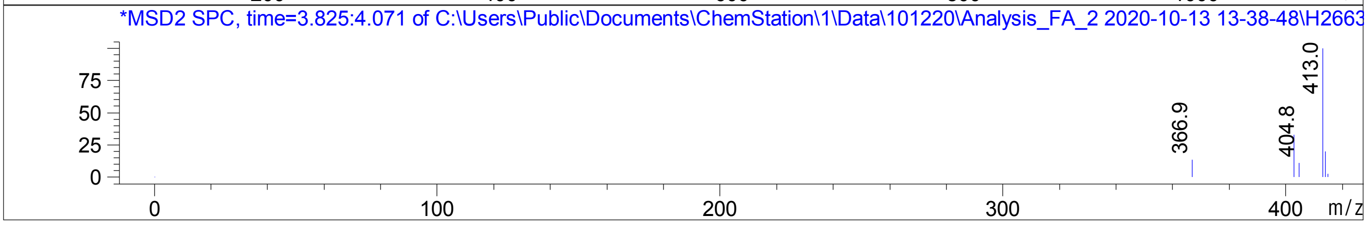

Tue, 13.->13.10.2020 S-> E D1F-B4 Acq. Method C:\Users\ ->

H2663691

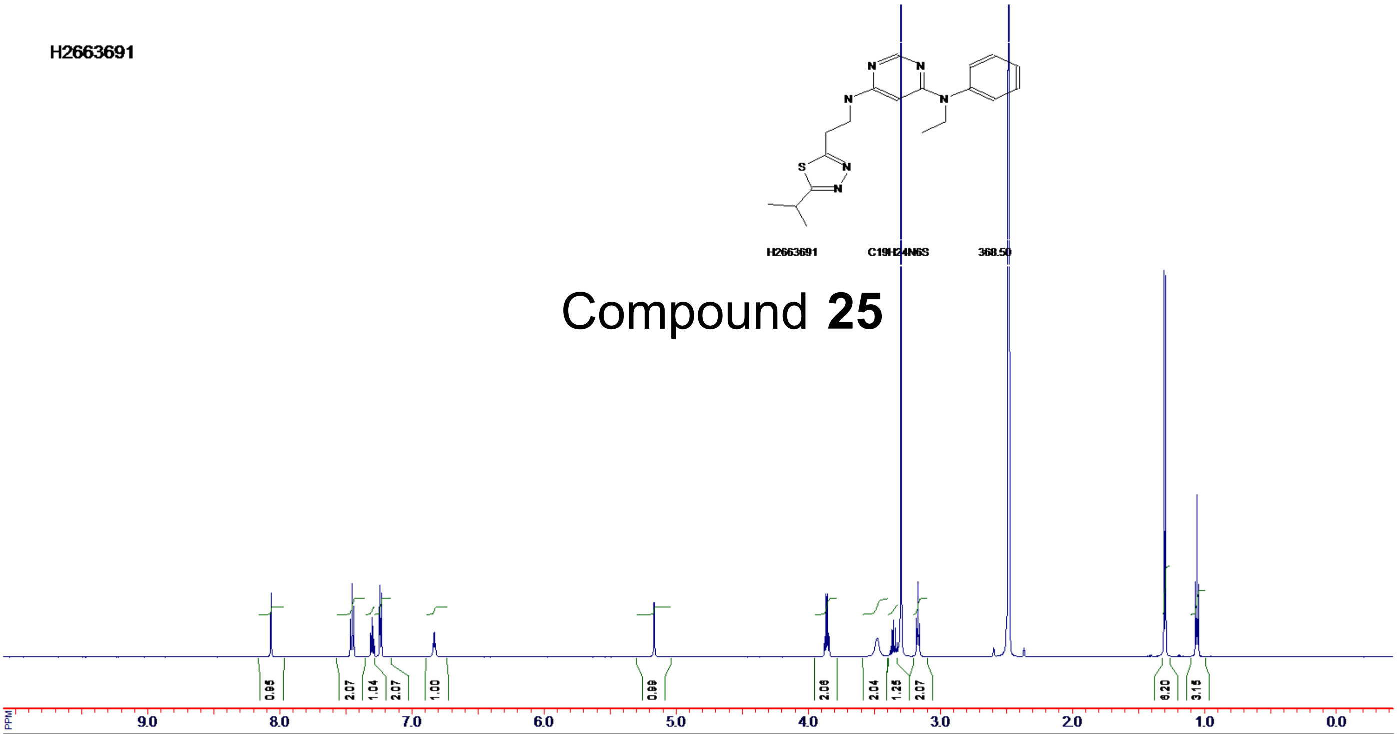

Compound **25**

File name: H2663691	Honchar	SF: 599.7811 MHz	NSC: 0	PW: 4.40 usec, RG: 44	St: 131072
Date: 26-Nov-2020	Solvent: dmso	SW: 11364 Hz	TE: 298 K	AQ: 2.82 sec, RD: 0.00 sec	H2663691

Compound 26 Sample H2660983 0.1%FA-ACN 5-95%ACN 6 min. Zorbax-Eclipse-Plus C18 4,6 x 100mm, 3.5 mkm.

#	Time	Area%
1	5.738	100.00

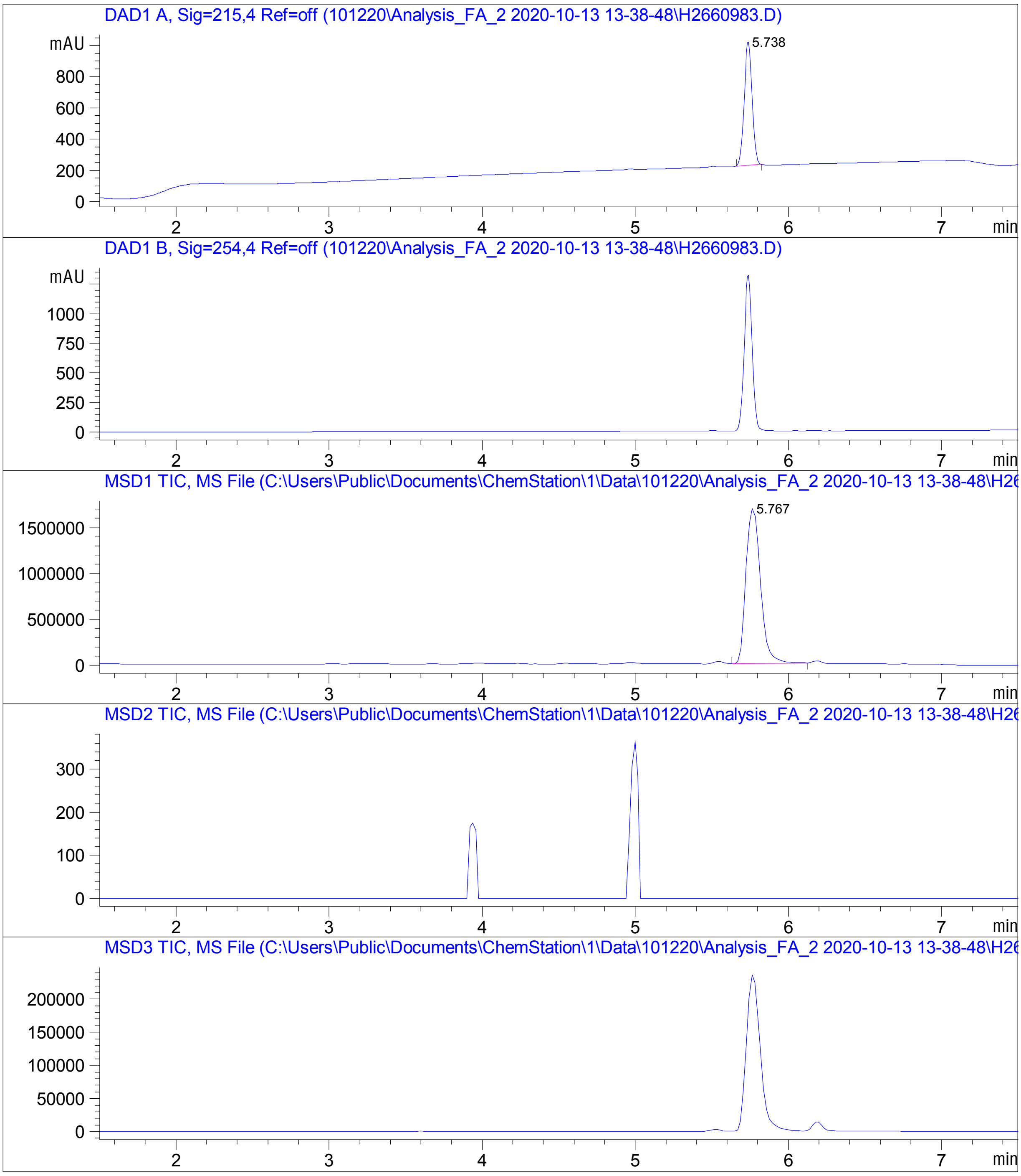

RT 5.767

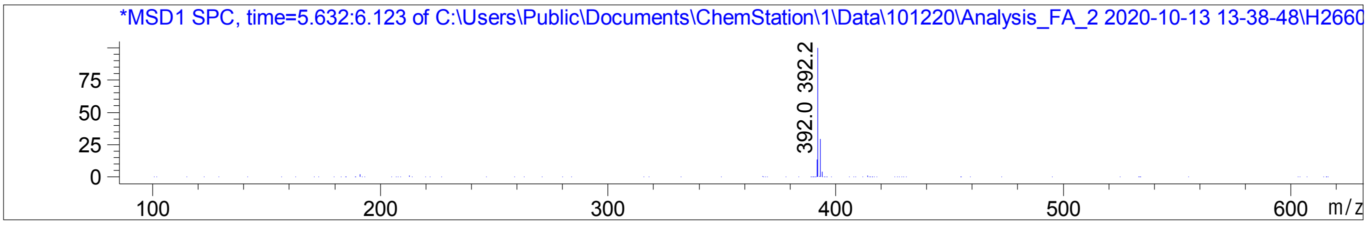

Tue, 13.->13.10.2020 S-> E D1F-B5 Acq. Method C:\Users\ ->

H2660983

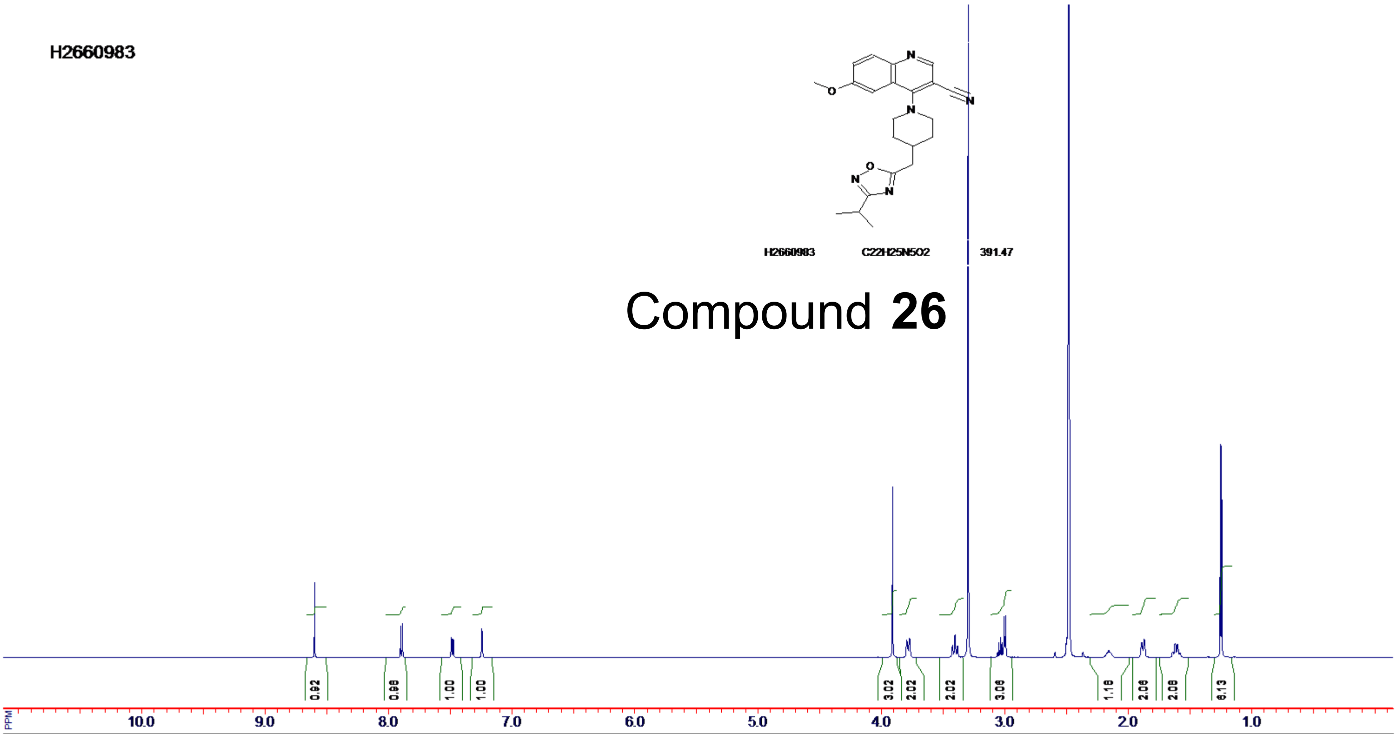

Compound **26**

File name: H2660983	Honchar	SF: 599.7811 MHz	NSC: 0	PW: 4.40 usec, RG: 44	SI: 131072
Date: 26-Nov-2020	Solvent: dmso	SW: 11364 Hz	TE: 298 K	AQ: 2.82 sec, RD: 0.00 sec	H2660983

Compound **27**

Sample H2663777

0.1%FA-ACN 5-95%ACN 6 min. Zorbax-Eclipse-Plus C18 4,6 x 100mm, 3.5 mkm.

#	Time	Area%
1	6.171	100.00

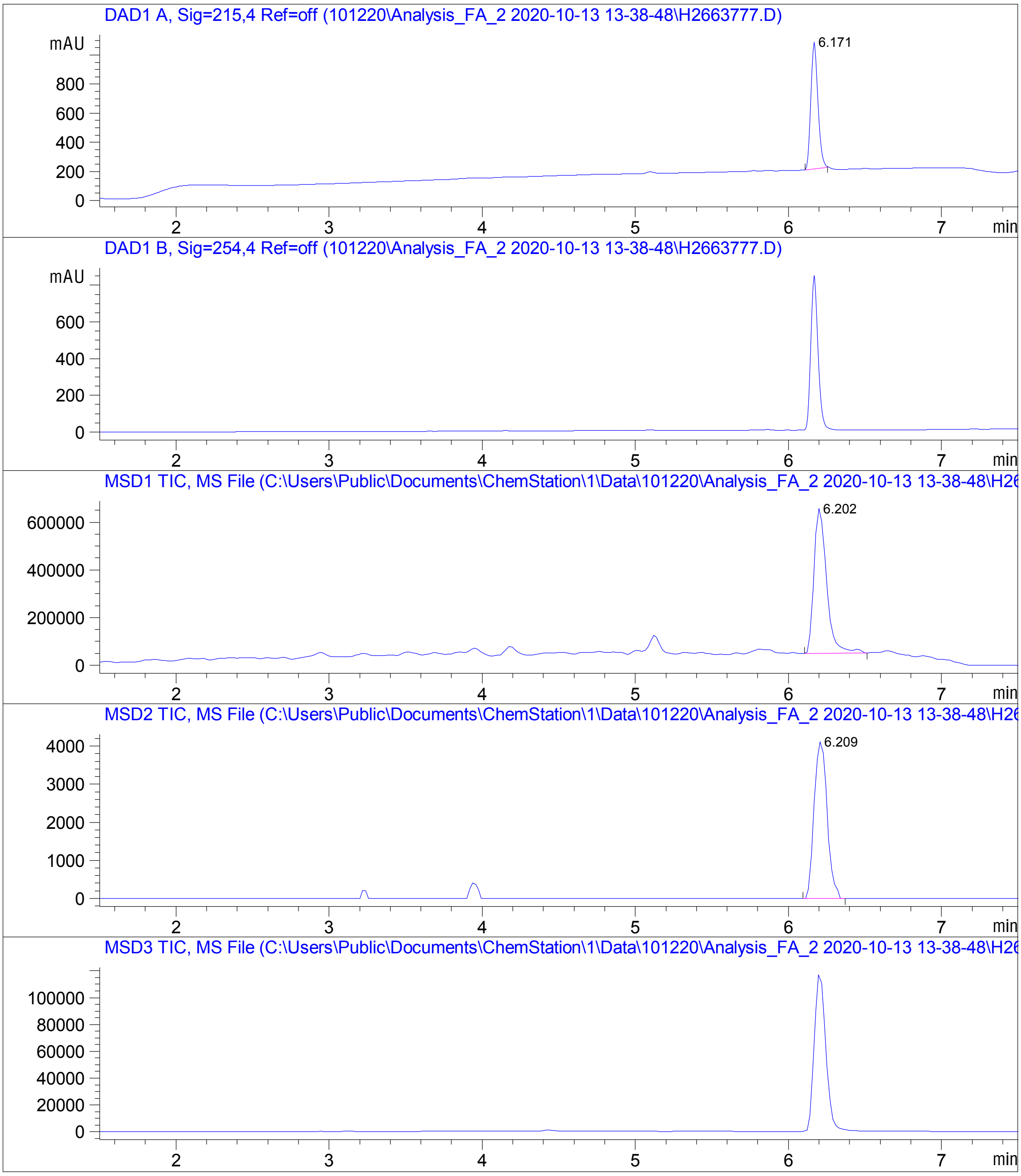

RT 6.202

RT 6.209

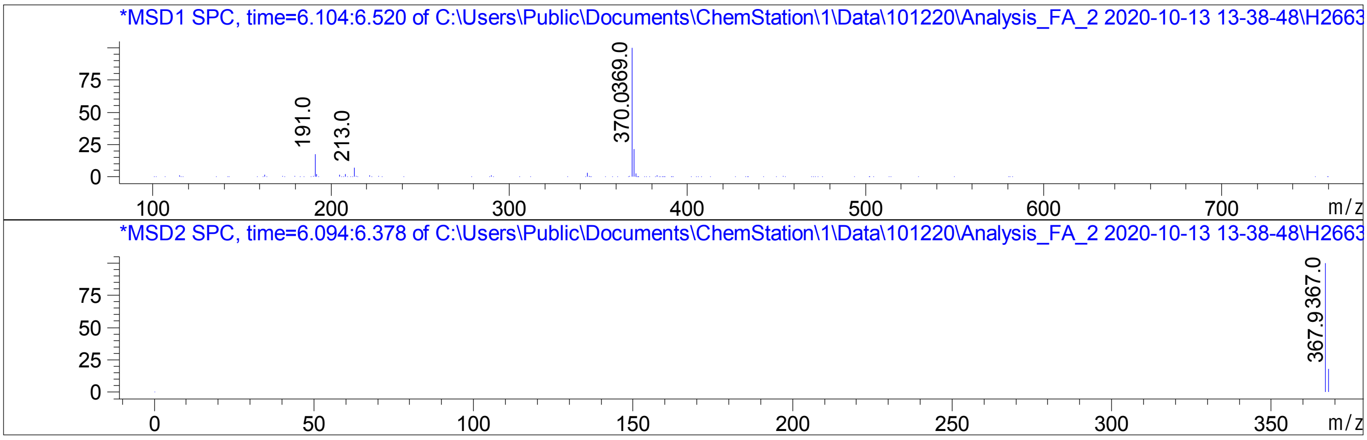

Tue, 13.->13.10.2020 S-> E D1F-B1 Acq. Method C:\Users\ ->

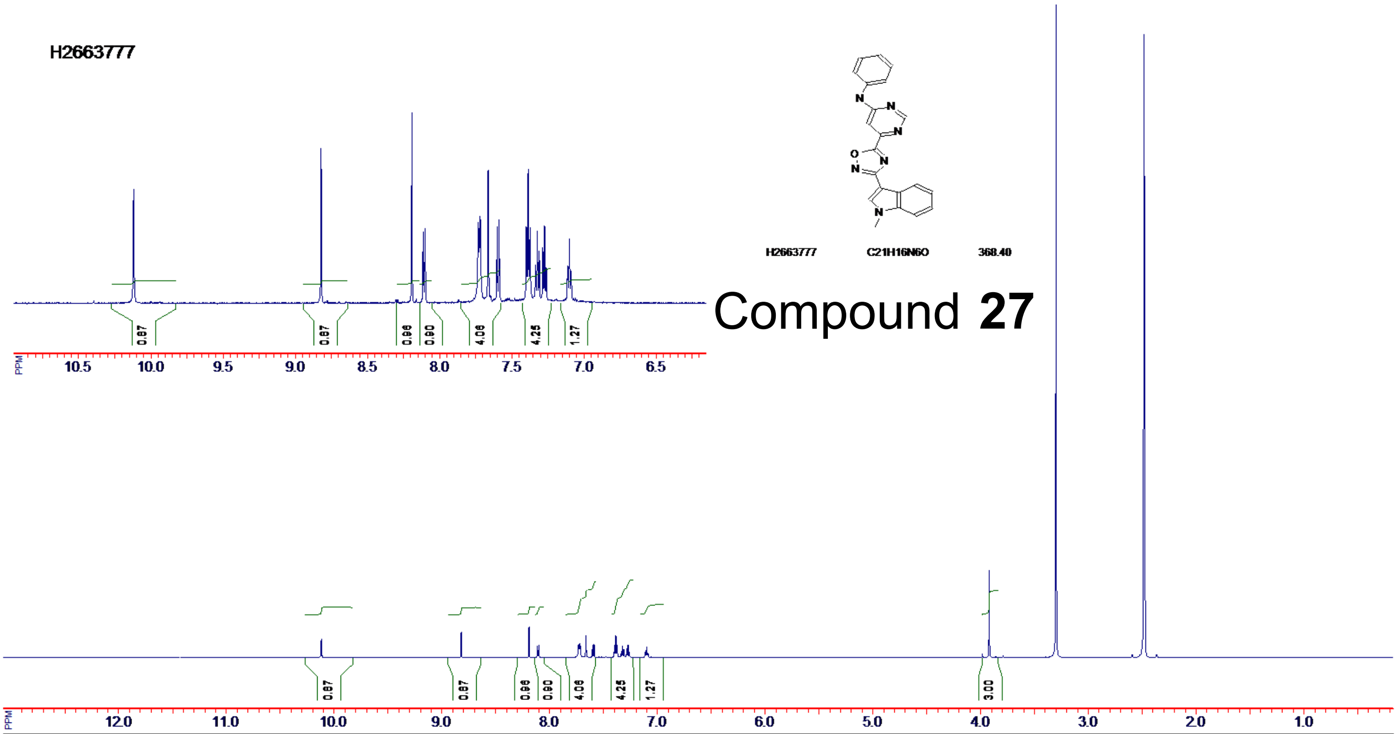

Compound **27**

File name: H2663777	Honchar	SF: 599.7811 MHz	NSC: 0	PW: 4.40 usec, RG: 44	St: 131072
Date: 26-Nov-2020	Solvent: dmso	SW: 11364 Hz	TE: 298 K	AQ: 2.82 sec, RD: 0.00 sec	H2663777

Compound **28**

Sample H2670384

0.1%FA-ACN 5-95%ACN 6 min. Zorbax-Eclipse-Plus C18 4,6 x 100mm, 3.5 mkm.

#	Time	Area%
1	2.648	0.35
2	3.153	95.66
3	4.237	2.50
4	4.694	1.49

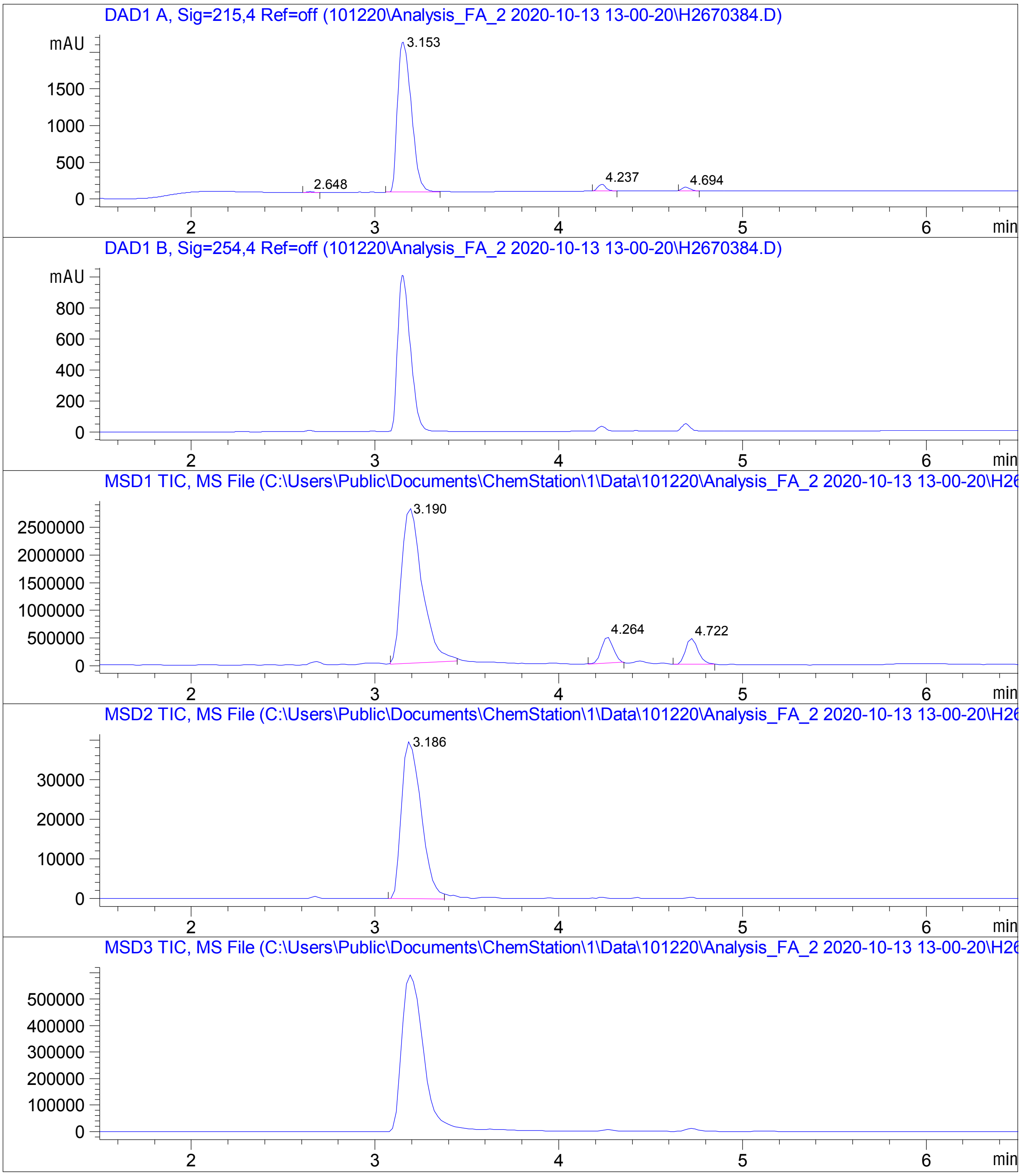

RT 3.190

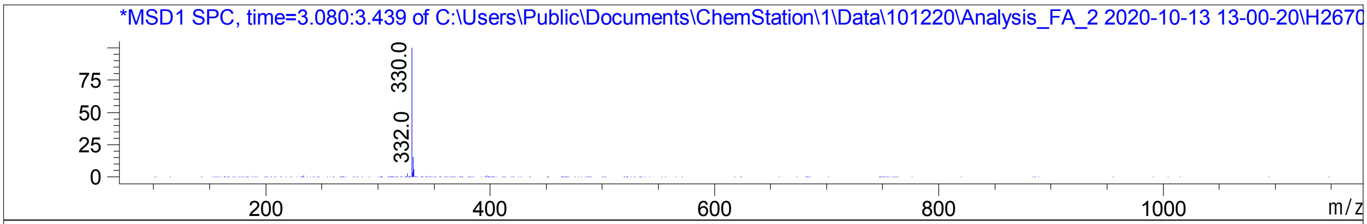

RT 4.264

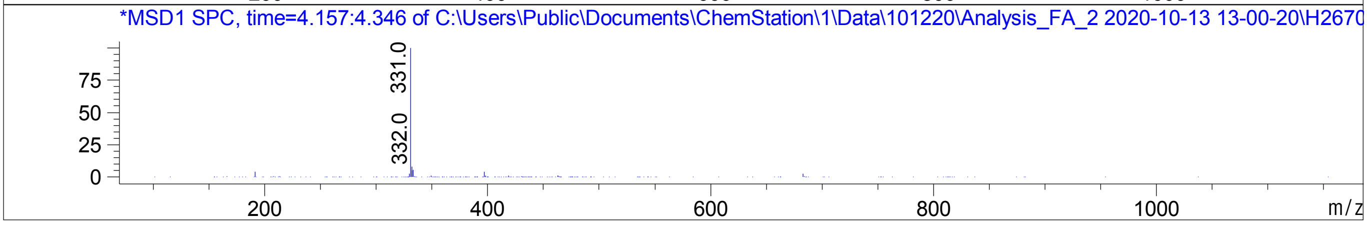

RT 4.722

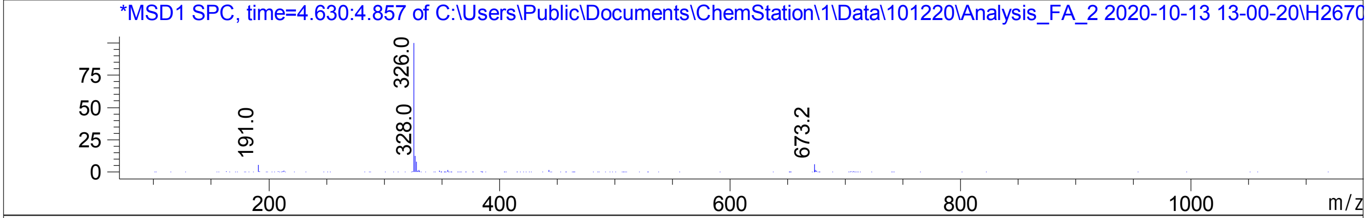

RT 3.186

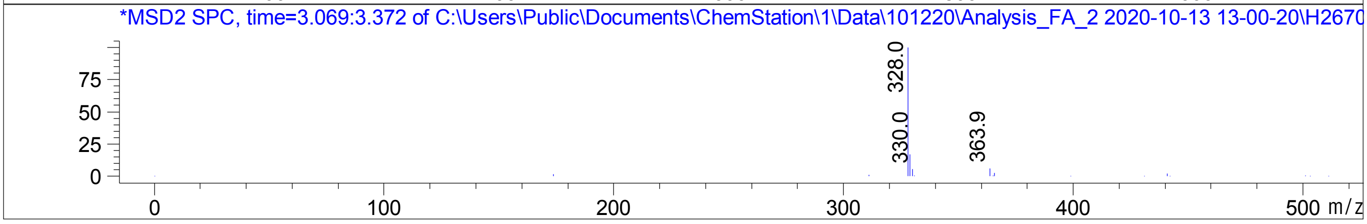

Compound **28**

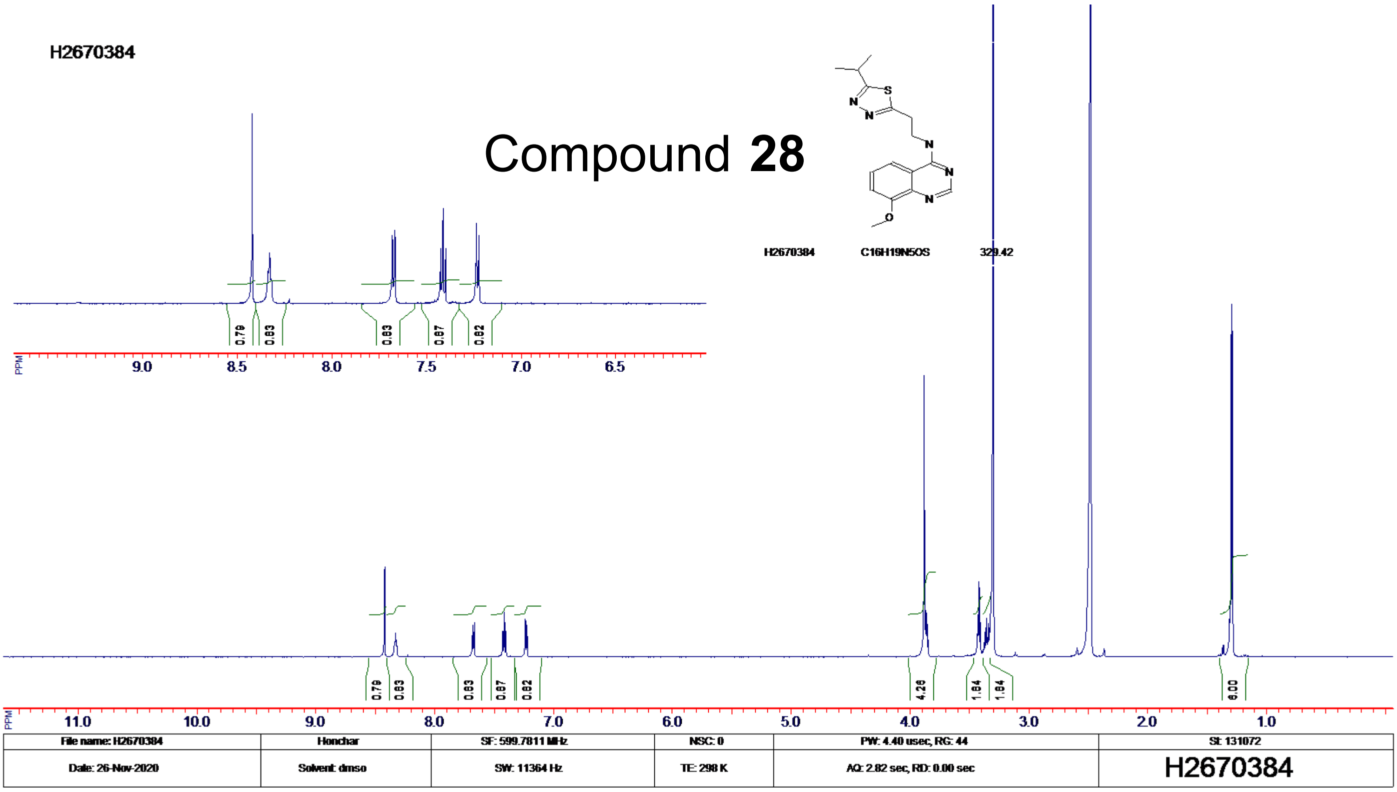
H2670384
H2670384 C16H19N5OS 329.42
0.79 0.83 0.83 0.87 0.82
PPM 9.0 8.5 8.0 7.5 7.0 6.5
0.79 0.83 0.83 0.87 0.82 4.26 1.84 1.84 6.00
PPM 11.0 10.0 9.0 8.0 7.0 6.0 5.0 4.0 3.0 2.0 1.0
File name: H2670384
Honchar
SF: 599.7811 MHz
NSC: 0
PW: 4.40 usec, RG: 44
St: 131072
Date: 26-Nov-2020
Solvent: dmso
SW: 11364 Hz
TE: 298 K
AQ: 2.82 sec, RD: 0.00 sec
H2670384

```
MaxPeak:  100.00%
Ret_Time: 1.245 min
```

S089971$6

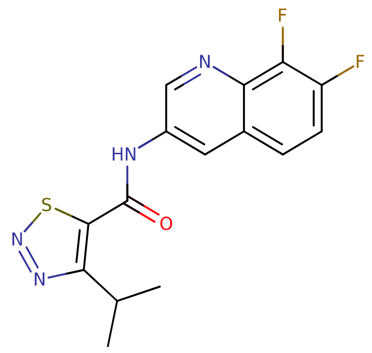

Mol Wt 334.34

Exact Mass 334.08

```
#  Time   Area%
-----------------
1  1.245  100.00
```

Compound **29**

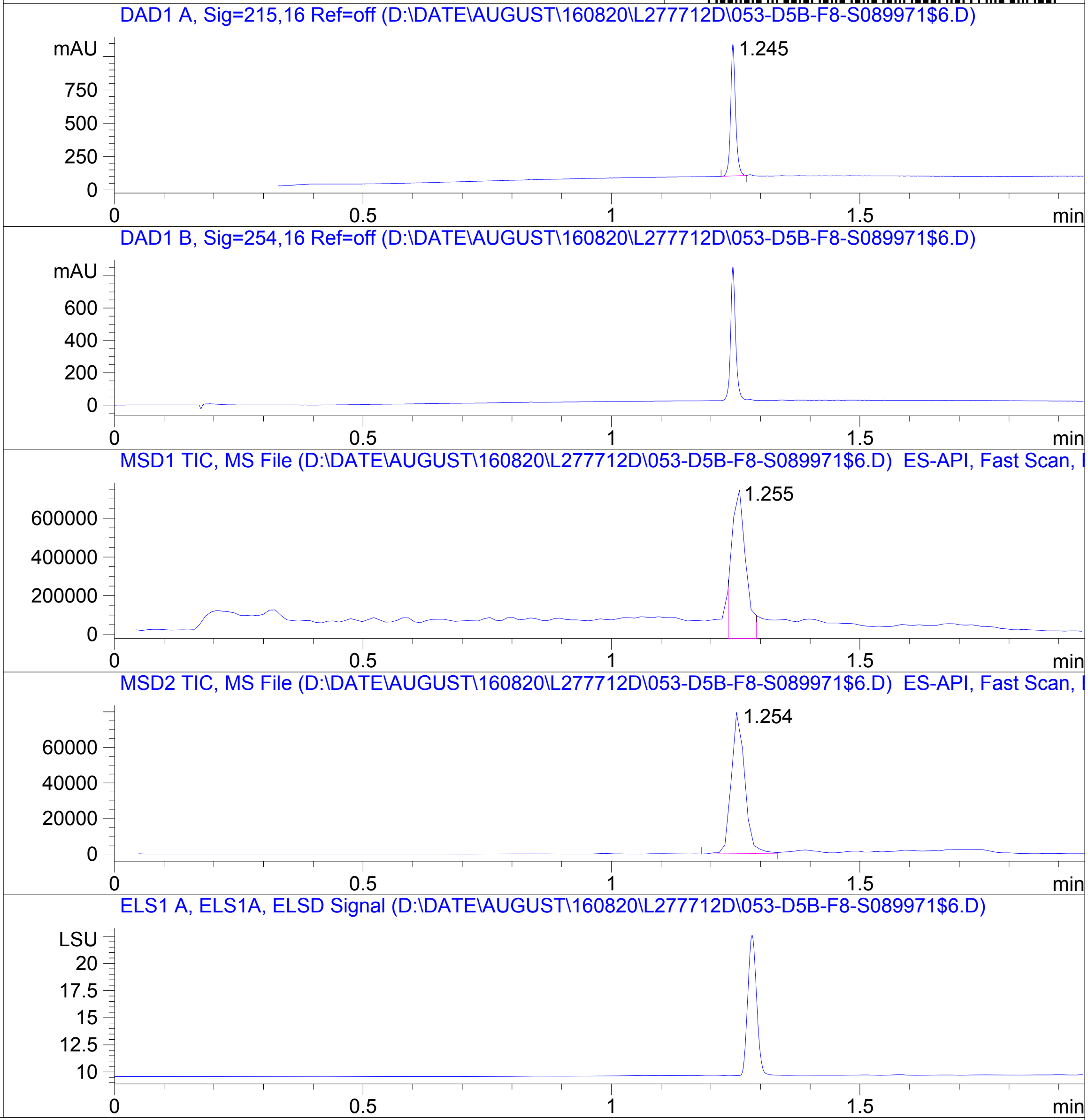

RT 1.255

RT 1.254

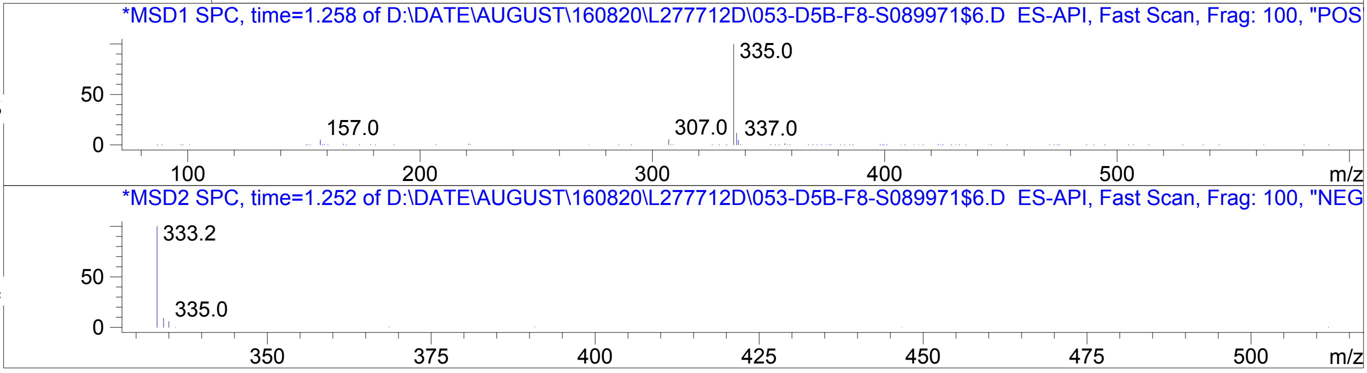


```
Inj.Date 8/15/2020          T      &lt;invalid&gt;          Acq. Method C:\Users\ ->    ->
```

H2563045

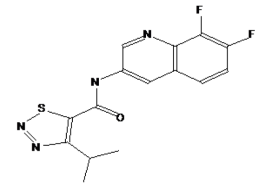

H2563045 C15H12F2N4OS 334.34

Compound **29**

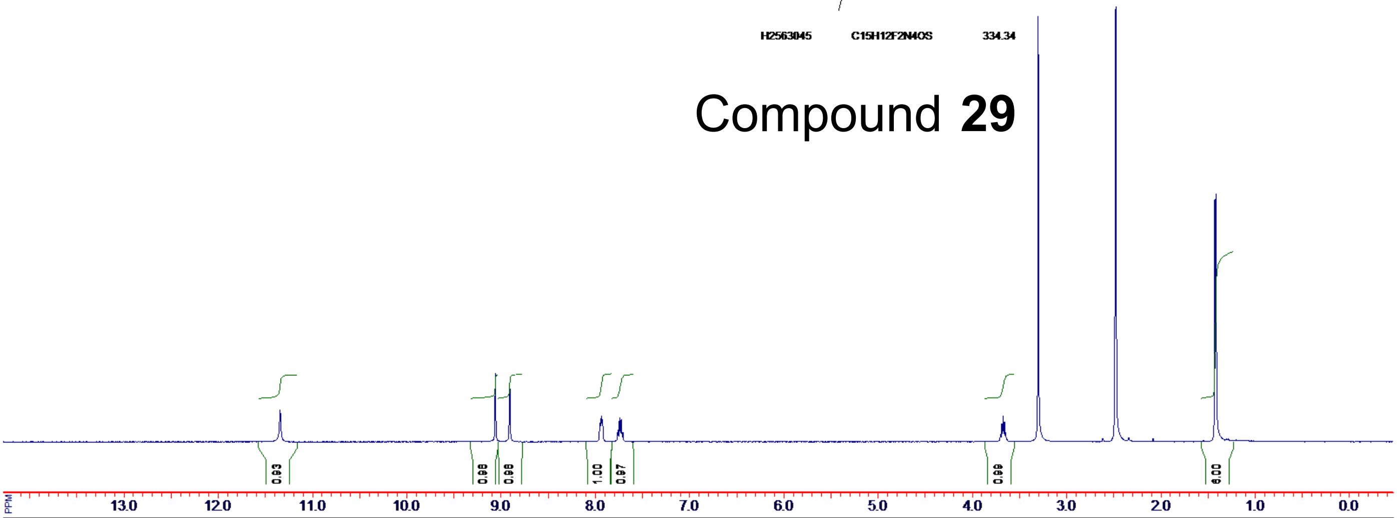

File name: H2563045	Honchar	SF: 499.8203 MHz	NSC: 0	PW: 12.40 usec, RG: 14	SI: 65536
Date: 01-Dec-2020	Solvent: dmso	SW: 9542 Hz	TE: 298 K	AQ: 1.68 sec, RD: 0.00 sec	H2563045

Compound **30**

MaxPeak: 100.00%
Ret_Time: 1.273 min

S089954$3

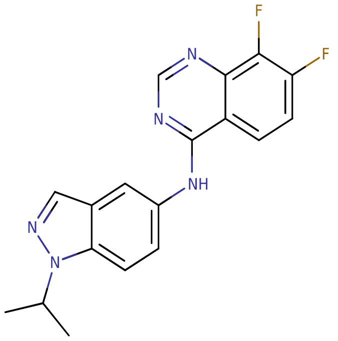

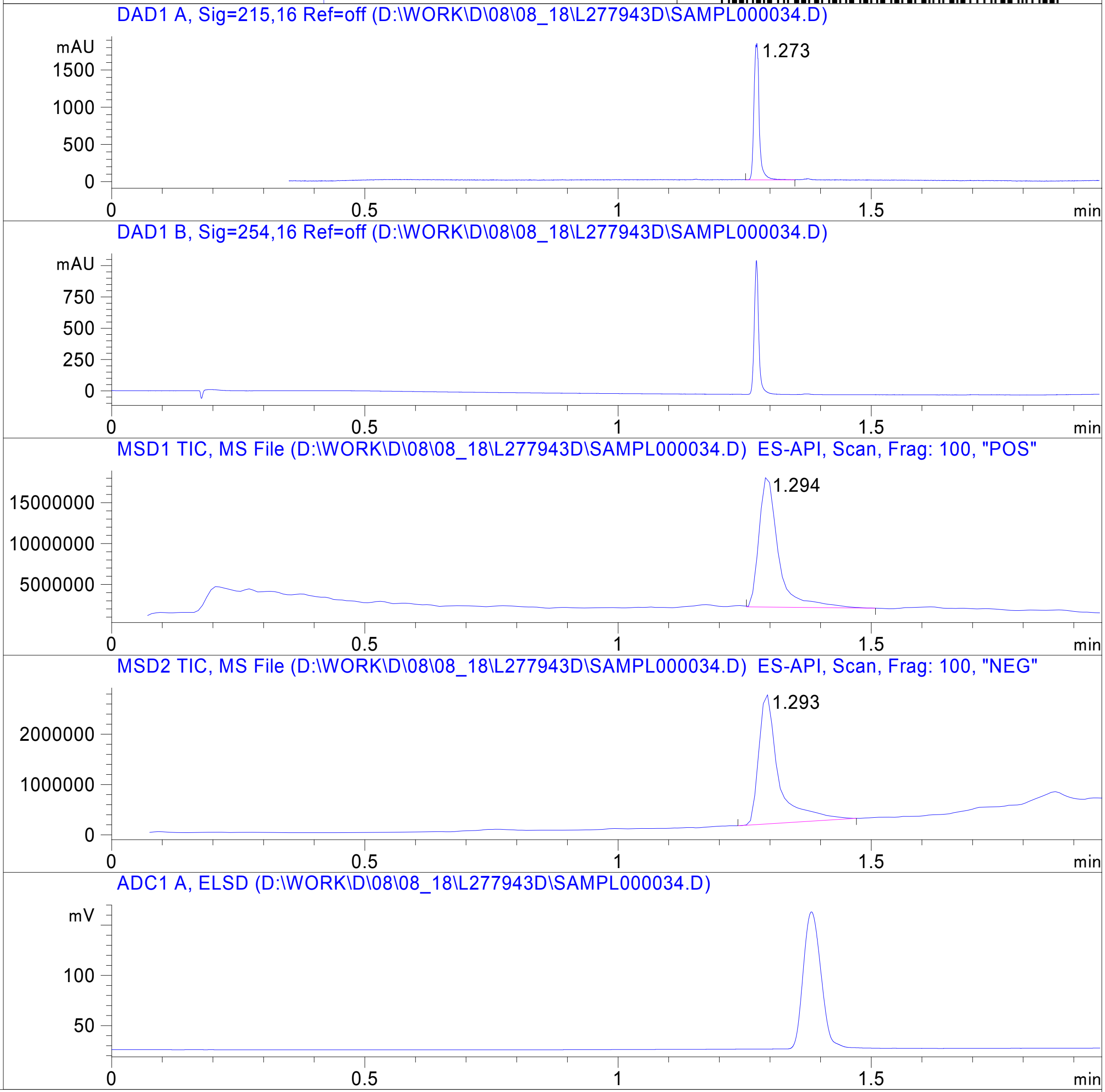

Mol Wt 339.34

Exact Mass 339.15

#	Time	Area%
1	1.273	100.00

RT 1.294

RT 1.293

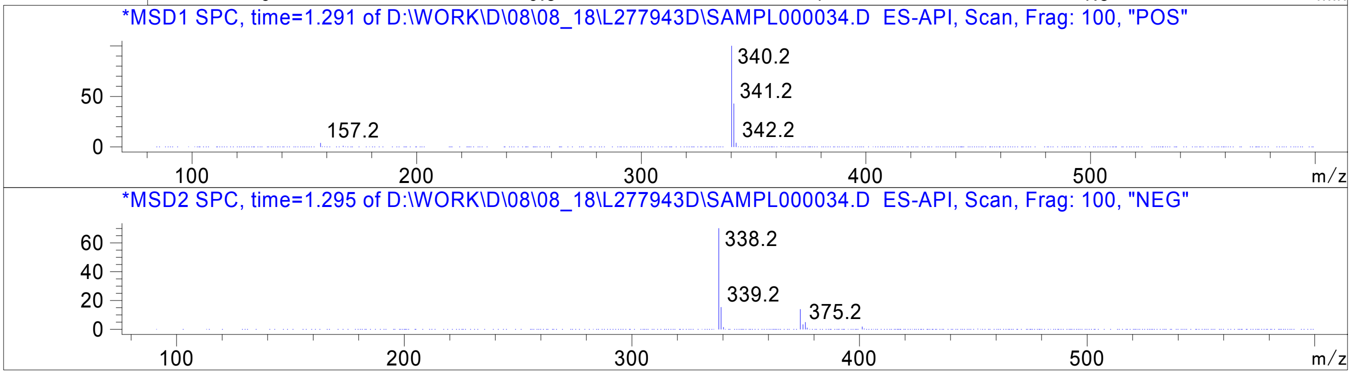

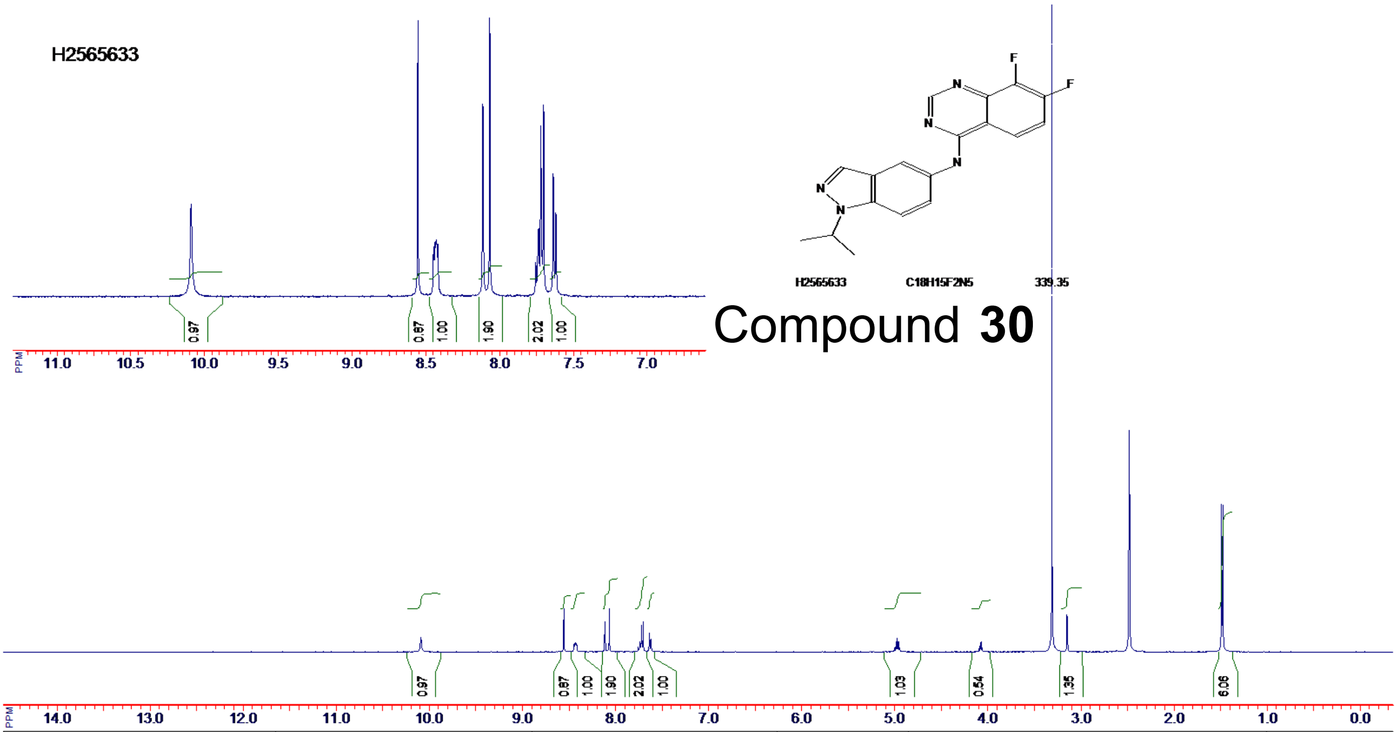

Compound **30**

File name: H2565633	Operator: Victorova	SF: 499.8203 MHz	NSC: 0	PW: 12.40 usec, RG: 28	SI: 65536
Date: 01-Dec-2020	Solvent: dmso	SW: 9542 Hz	TE: 298 K	AQ: 1.68 sec, RD: 0.00 sec	

Compound **31**

Sample H2674289 — 0.1%FA-ACN 5-95%ACN 6 min. Zorbax-Eclipse-Plus C18 4,6 x 100mm, 3.5 mkm.

#	Time	Area%
1	2.259	2.14
2	3.763	0.89
3	6.448	0.80
4	6.849	96.18

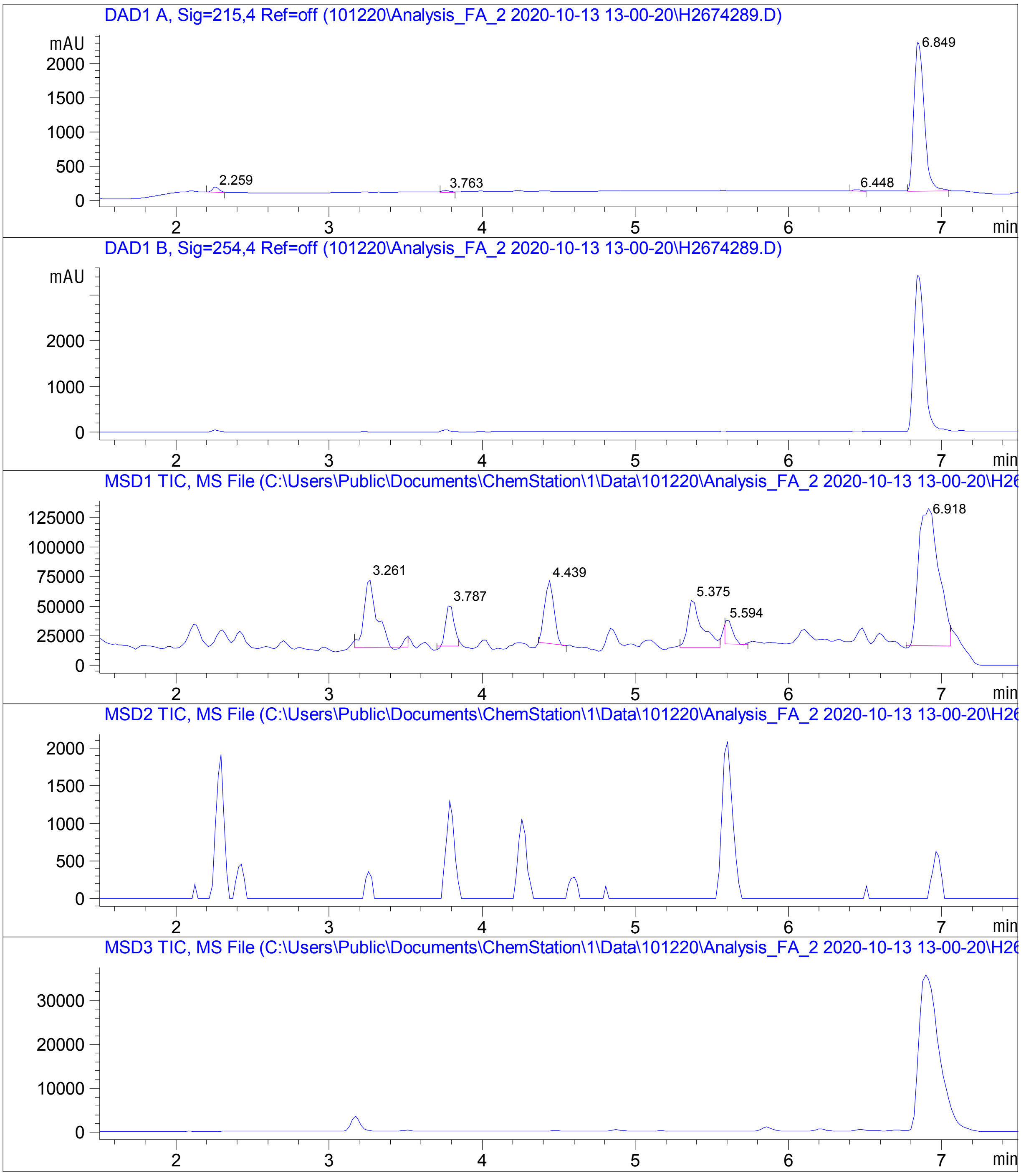

RT 3.261

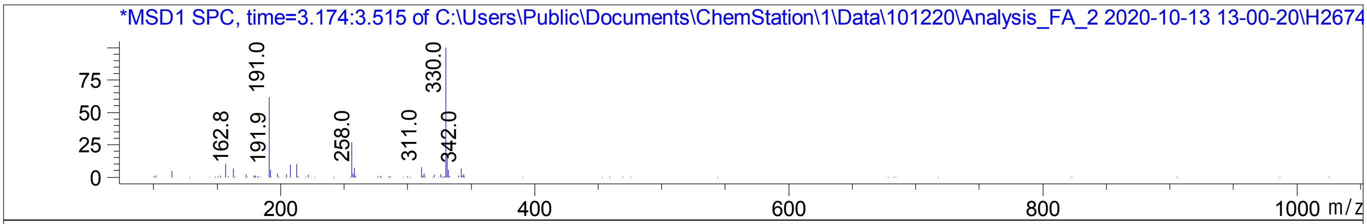

RT 3.787

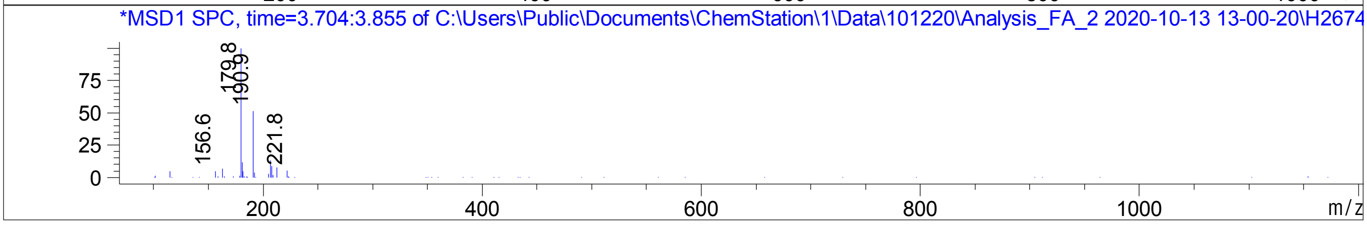

RT 4.439

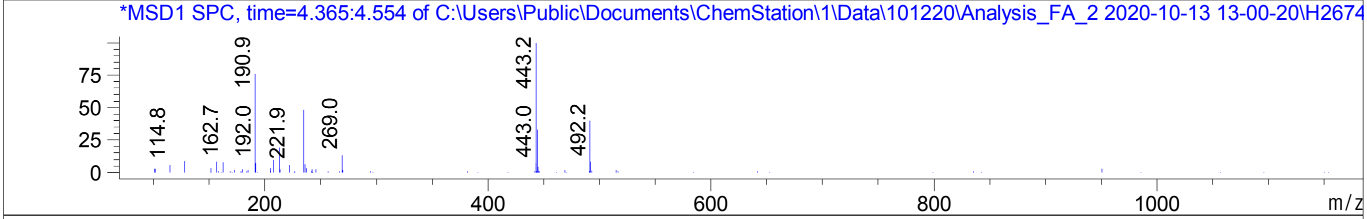

RT 5.375

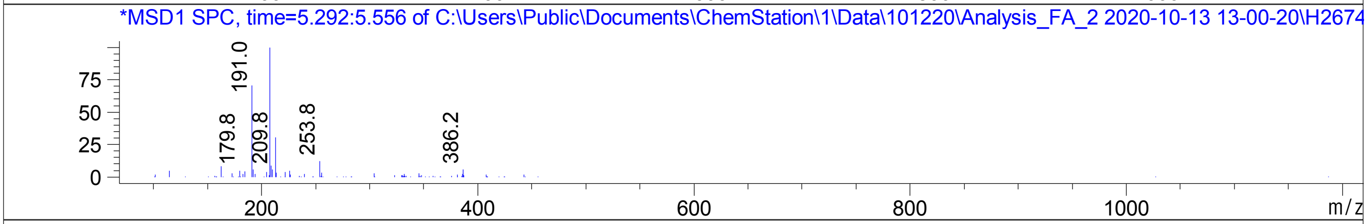

RT 5.594

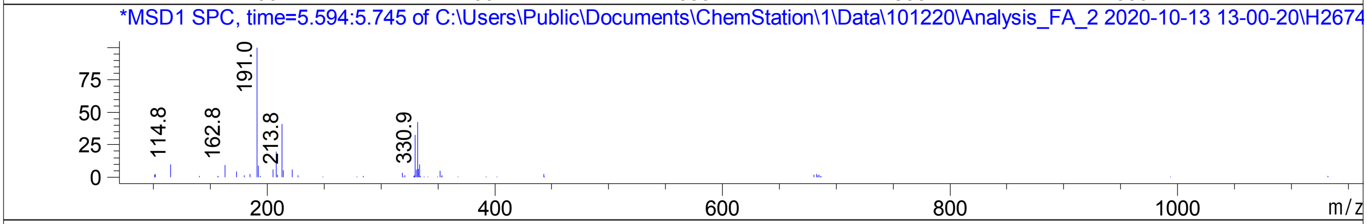

RT 6.918

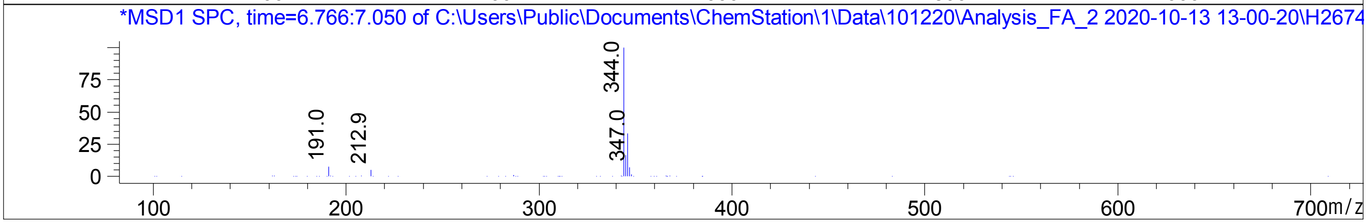

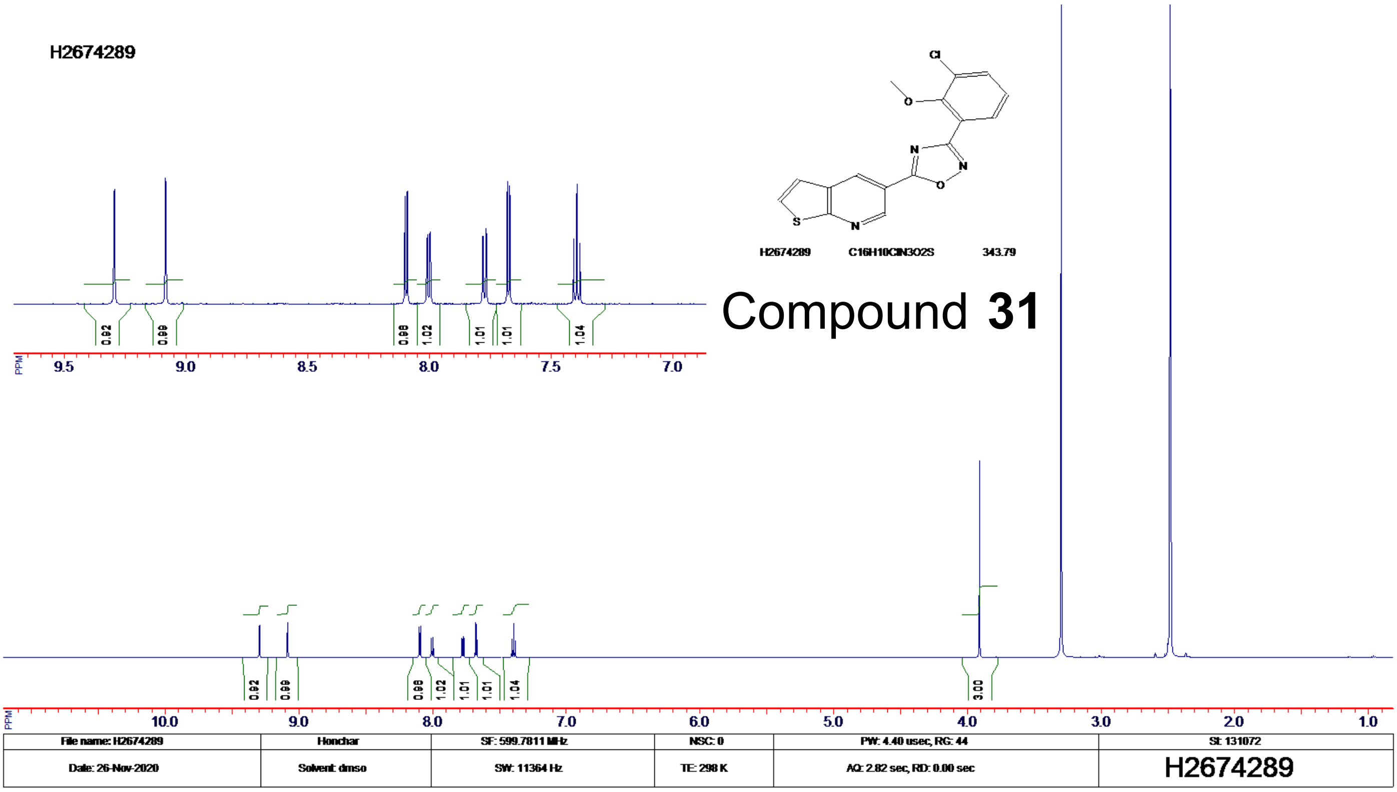

H2674289
Cl
O
N
N
O
S
N
H2674289 C16H10ClN3O2S 343.79
0.92
0.99
0.98
1.02
1.01
1.01
1.04
3.00
PPM
9.5 9.0 8.5 8.0 7.5 7.0
10.0 9.0 8.0 7.0 6.0 5.0 4.0 3.0 2.0 1.0
File name: H2674289
Honchar
SF: 599.7811 MHz
NSC: 0
PW: 4.40 usec, RG: 44
SI: 131072
Date: 26-Nov-2020
Solvent: dmso
SW: 11364 Hz
TE: 298 K
AQ: 2.82 sec, RD: 0.00 sec
H2674289

Compound **31**

Compound **32**

MaxPeak: 100.00%
Ret_Time: 1.254 min

S491339$5

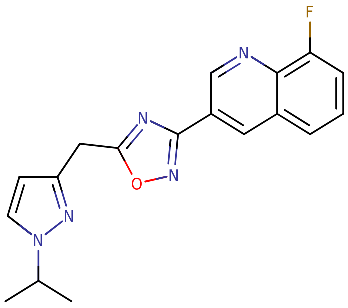

Mol Wt 337.35

Exact Mass 337.15

#	Time	Area%
1	1.254	100.00

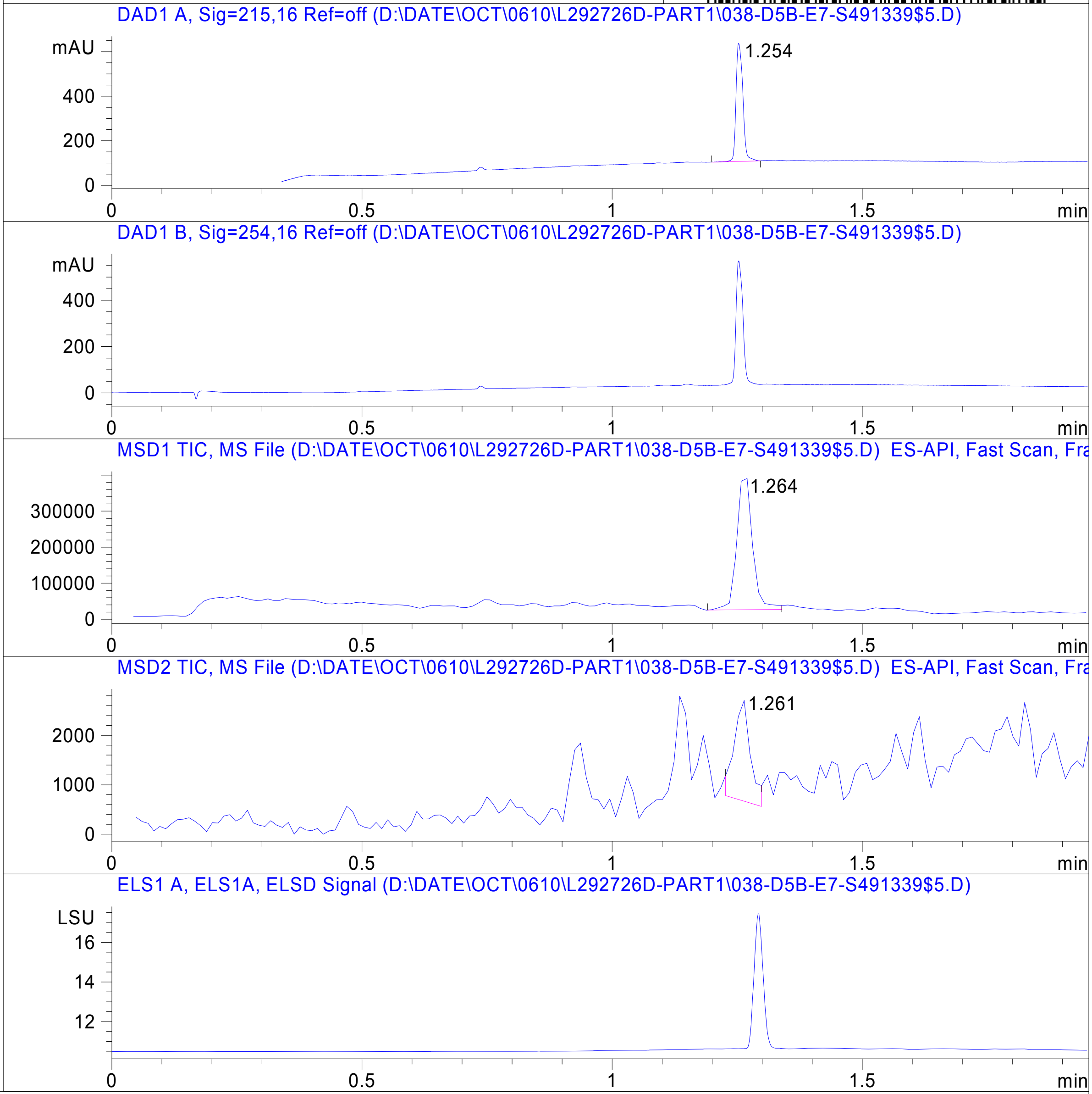

RT 1.264

RT 1.261

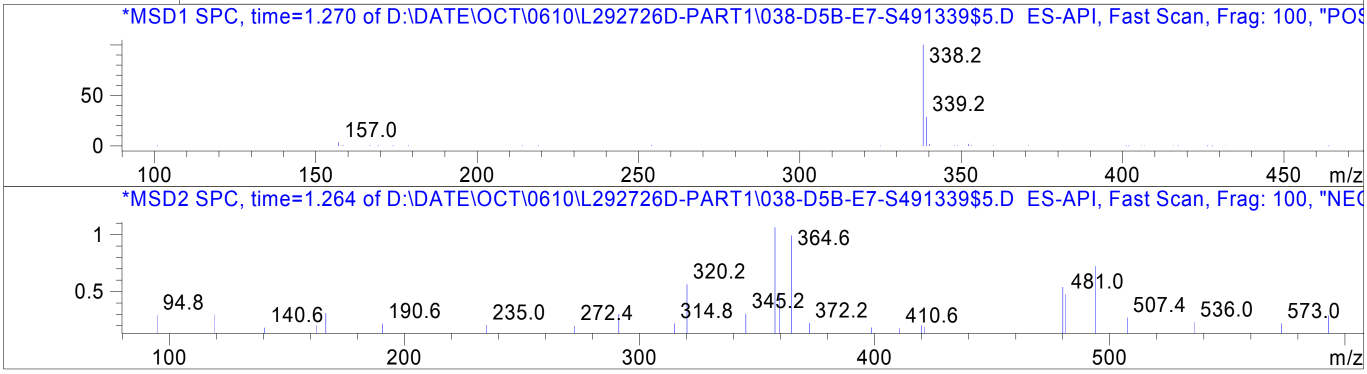

Inj.Date 10/6/2020 Y Acq. Method C:\Users\ -> ->

Compound **32**

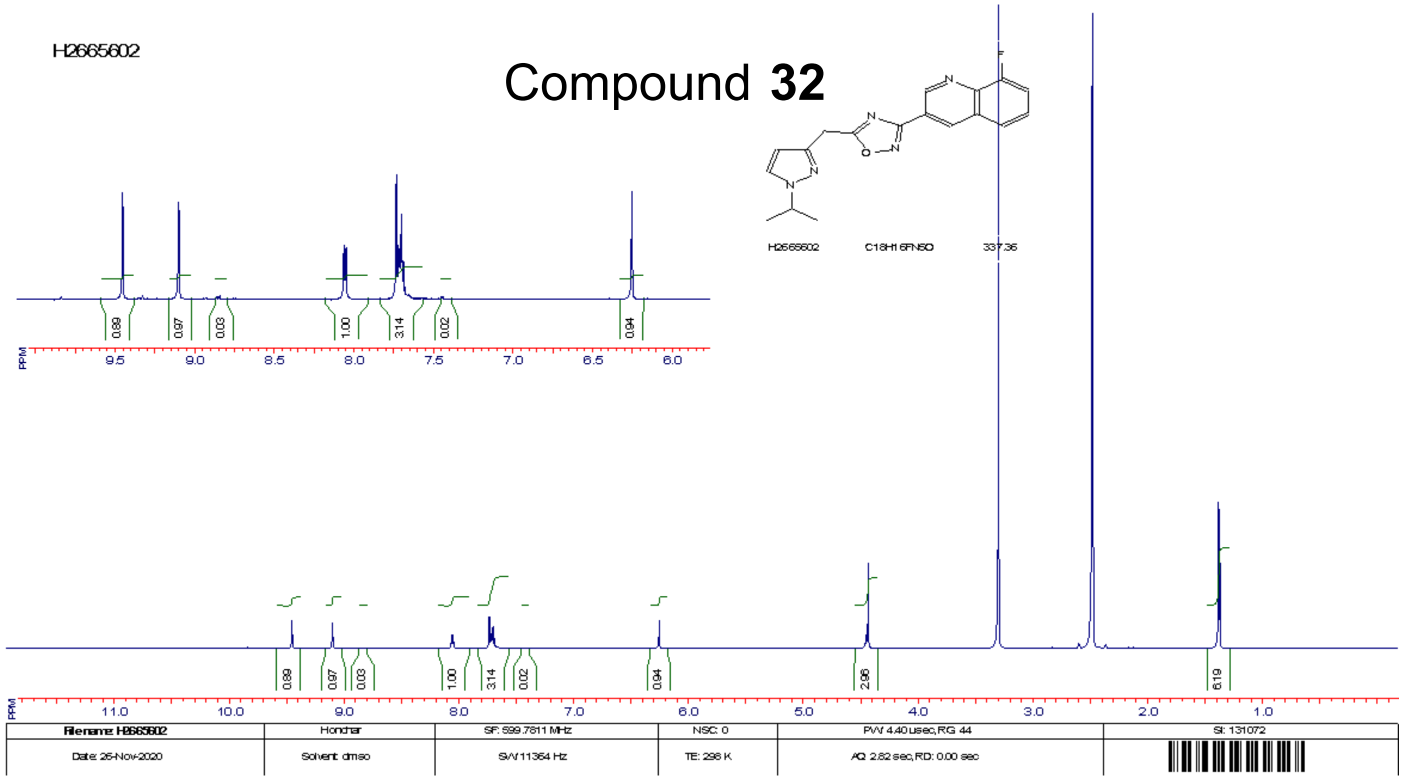

H2665602
H2665602 C18H16FN5O 337.36
N
F
O
PPM
9.5 9.0 8.5 8.0 7.5 7.0 6.5 6.0
0.89 0.97 0.03 1.00 3.14 0.02 0.94 2.96 6.19
11.0 10.0 9.0 8.0 7.0 6.0 5.0 4.0 3.0 2.0 1.0
Filename: H2665602
Honchar
SF: 599.7811 MHz
NSC: 0
PW 4.40 usec, RG 44
SI: 131072
Date: 25-Nov-2020
Solvent: dmso
SW 11364 Hz
TE: 298 K
AQ: 2.82 sec, RD: 0.00 sec

Compound **33**

```
MaxPeak:  100.00%
Ret_Time: 1.449 min
```

S089995$3

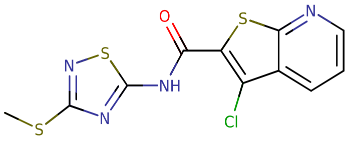

Mol Wt 342.85

Exact Mass 341.94

```
#  Time   Area%
-----------------
1  1.449  100.00
```

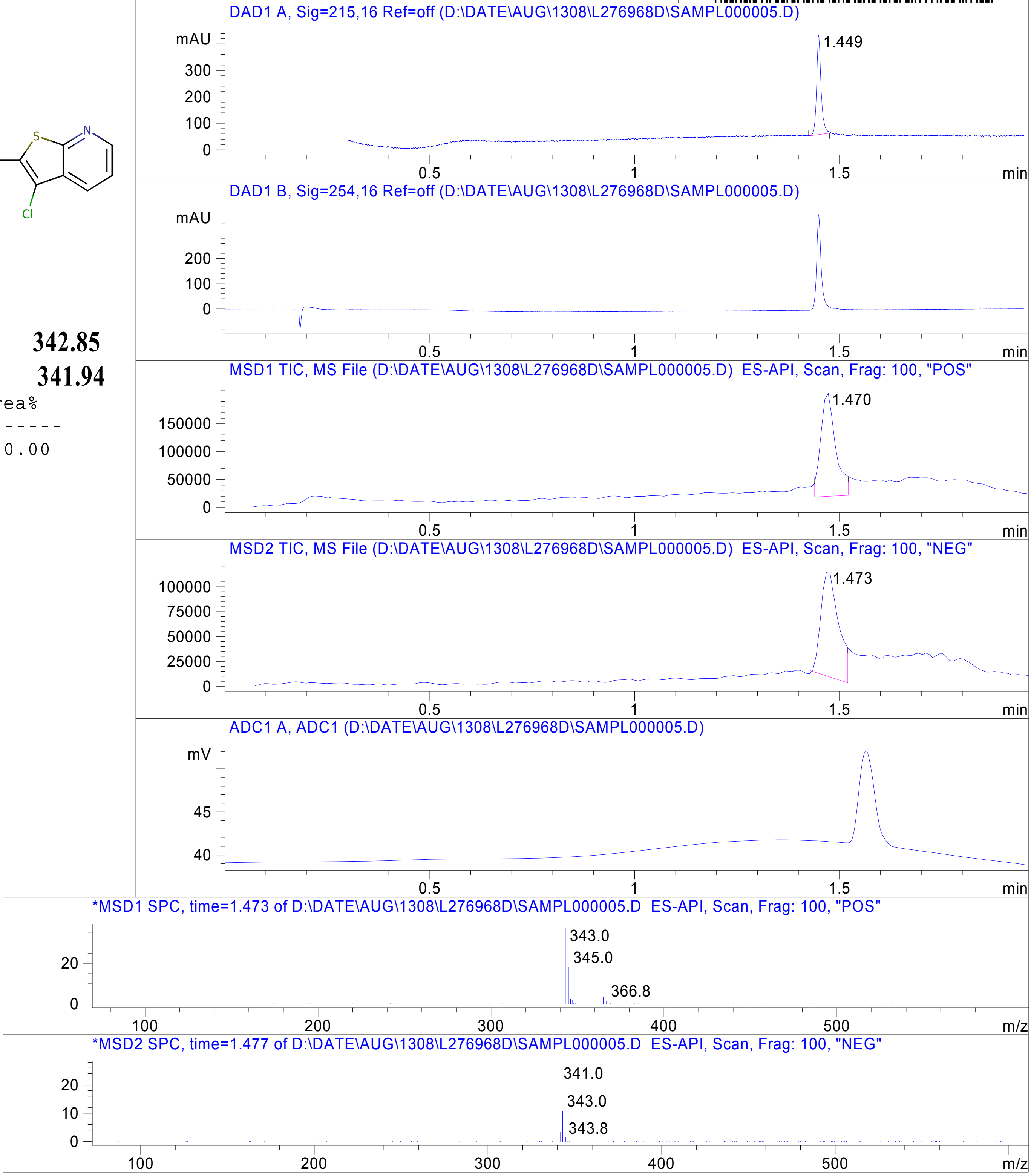

RT 1.470

RT 1.473

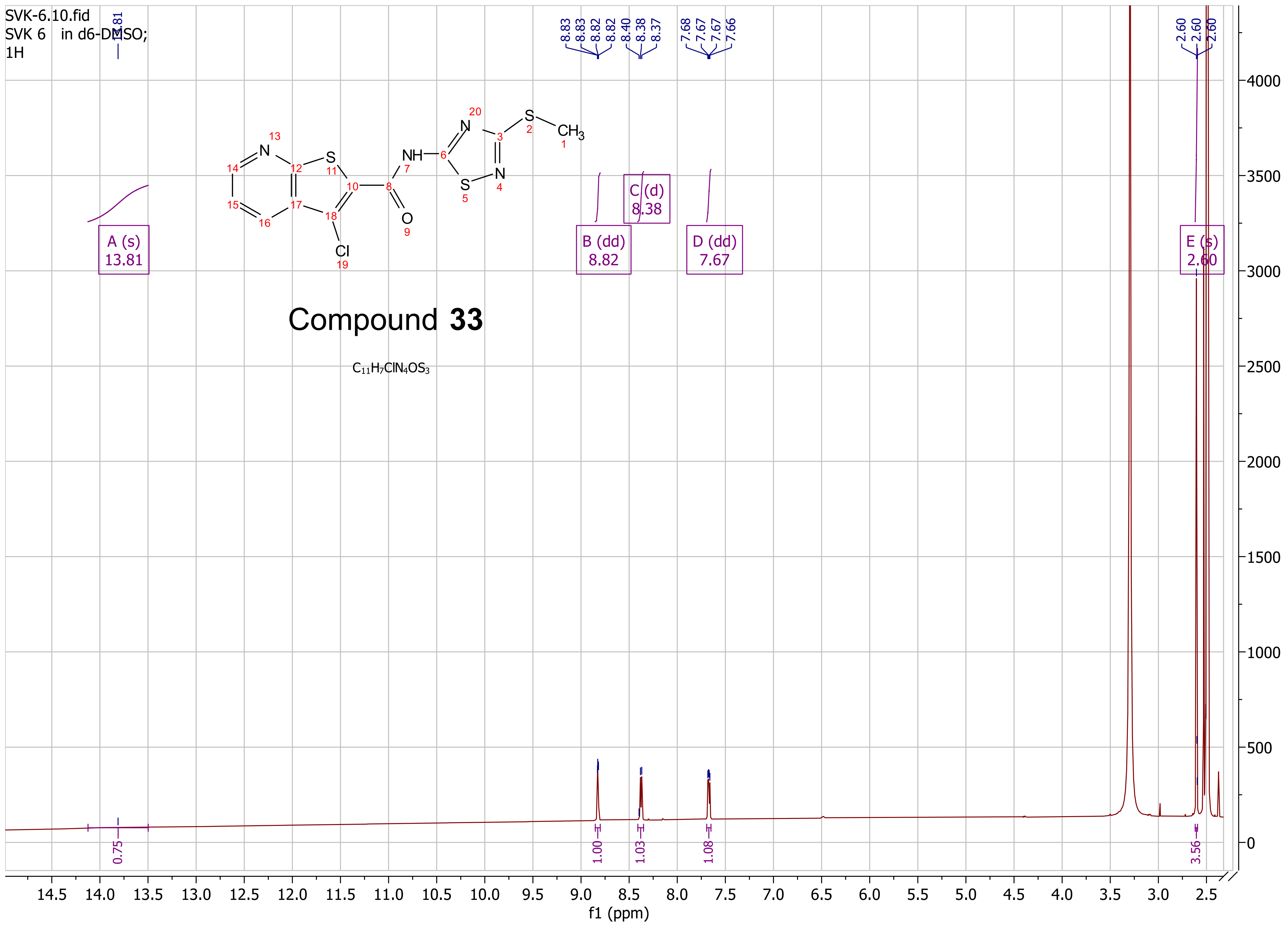

SVK-6.10.fid
SVK 6 in d6-DMSO;
1H
13.81
8.83
8.83
8.82
8.82
8.40
8.38
8.37
7.68
7.67
7.67
7.66
2.60
2.60
2.60
A (s)
13.81
B (dd)
8.82
C (d)
8.38
D (dd)
7.67
E (s)
2.60
Compound **33**
$C_{11}H_7ClN_4OS_3$
0.75
1.00
1.03
1.08
3.56
f1 (ppm)
